# Synthetically derived BiAux modulates auxin co-receptor activity to stimulate lateral root formation

**DOI:** 10.1093/plphys/kiae090

**Published:** 2024-02-20

**Authors:** Mary Paz González-García, Angela Sáez, Mónica Lanza, Pilar Hoyos, Estefano Bustillo-Avendaño, Luis F Pacios, Ana Gradillas, Miguel A Moreno-Risueno, María José Hernaiz, Juan C del Pozo

**Affiliations:** Centro de Biotecnología y Genómica de Plantas (UPM-INIA/CSIC), Universidad Politécnica de Madrid (UPM)—Instituto Nacional de Investigación y Tecnología Agraria y Alimentaria-CSIC (INIA/CSIC), Campus Montegancedo, 28223 Pozuelo de Alarcón, Madrid, Spain; Departamento de Biotecnología-Biología Vegetal, Escuela Técnica Superior de Ingeniería Agronómica, Alimentaria y de Biosistemas, Universidad Politécnica de Madrid (UPM), 28040 Madrid, Spain; Centro de Biotecnología y Genómica de Plantas (UPM-INIA/CSIC), Universidad Politécnica de Madrid (UPM)—Instituto Nacional de Investigación y Tecnología Agraria y Alimentaria-CSIC (INIA/CSIC), Campus Montegancedo, 28223 Pozuelo de Alarcón, Madrid, Spain; Universidad Francisco de Vitoria, Facultad de Ciencias Experimentales, Edificio E., 28223 Pozuelo de Alarcón, Madrid, Spain; Centro de Biotecnología y Genómica de Plantas (UPM-INIA/CSIC), Universidad Politécnica de Madrid (UPM)—Instituto Nacional de Investigación y Tecnología Agraria y Alimentaria-CSIC (INIA/CSIC), Campus Montegancedo, 28223 Pozuelo de Alarcón, Madrid, Spain; Departamento de Química en Ciencias Farmacéuticas, Facultad de Farmacia, Universidad Complutense de Madrid, Plaza Ramón y Cajal s/n, 28040 Madrid, Spain; Centro de Biotecnología y Genómica de Plantas (UPM-INIA/CSIC), Universidad Politécnica de Madrid (UPM)—Instituto Nacional de Investigación y Tecnología Agraria y Alimentaria-CSIC (INIA/CSIC), Campus Montegancedo, 28223 Pozuelo de Alarcón, Madrid, Spain; Centro de Biotecnología y Genómica de Plantas (UPM-INIA/CSIC), Universidad Politécnica de Madrid (UPM)—Instituto Nacional de Investigación y Tecnología Agraria y Alimentaria-CSIC (INIA/CSIC), Campus Montegancedo, 28223 Pozuelo de Alarcón, Madrid, Spain; Centro de Metabolómica y Bioanálisis (CEMBIO), Facultad de Farmacia, Universidad San Pablo-CEU, CEU Universities, Urbanización Montepríncipe, 28660 Boadilla del Monte, Madrid, Spain; Centro de Biotecnología y Genómica de Plantas (UPM-INIA/CSIC), Universidad Politécnica de Madrid (UPM)—Instituto Nacional de Investigación y Tecnología Agraria y Alimentaria-CSIC (INIA/CSIC), Campus Montegancedo, 28223 Pozuelo de Alarcón, Madrid, Spain; Departamento de Biotecnología-Biología Vegetal, Escuela Técnica Superior de Ingeniería Agronómica, Alimentaria y de Biosistemas, Universidad Politécnica de Madrid (UPM), 28040 Madrid, Spain; Departamento de Química en Ciencias Farmacéuticas, Facultad de Farmacia, Universidad Complutense de Madrid, Plaza Ramón y Cajal s/n, 28040 Madrid, Spain; Centro de Biotecnología y Genómica de Plantas (UPM-INIA/CSIC), Universidad Politécnica de Madrid (UPM)—Instituto Nacional de Investigación y Tecnología Agraria y Alimentaria-CSIC (INIA/CSIC), Campus Montegancedo, 28223 Pozuelo de Alarcón, Madrid, Spain

## Abstract

The root system plays an essential role in plant growth and adaptation to the surrounding environment. The root clock periodically specifies lateral root prebranch sites (PBS), where a group of pericycle founder cells (FC) is primed to become lateral root founder cells and eventually give rise to lateral root primordia or lateral roots (LRs). This clock-driven organ formation process is tightly controlled by modulation of auxin content and signaling. Auxin perception entails the physical interaction of TRANSPORT INHIBITOR RESPONSE 1 (TIR1) or AUXIN SIGNALING F-BOX (AFBs) proteins with AUXIN/INDOLE-3-ACETIC ACID (Aux/IAA) repressors to form a co-receptor system. Despite the apparent simplicity, the understanding of how specific auxin co-receptors are assembled remains unclear. We identified the compound bis-methyl auxin conjugated with *N*-glucoside, or BiAux, in Arabidopsis (*Arabidopsis thaliana*) that specifically induces the formation of PBS and the emergence of LR, with a slight effect on root elongation. Docking analyses indicated that BiAux binds to F-box proteins, and we showed that BiAux function depends on TIR1 and AFB2 F-box proteins and AUXIN RESPONSE FACTOR 7 activity, which is involved in FC specification and LR formation. Finally, using a yeast (*Saccharomyces cerevisiae*) heterologous expression system, we showed that BiAux favors the assemblage of specific co-receptors subunits involved in LR formation and enhances AUXIN/INDOLE-3-ACETIC ACID 28 protein degradation. These results indicate that BiAux acts as an allosteric modulator of specific auxin co-receptors. Therefore, BiAux exerts a fine-tune regulation of auxin signaling aimed to the specific formation of LR among the many development processes regulated by auxin.

## Teaser

A synthetic version of a bis-methyl auxin conjugated with *N*-glucoside, or BiAux, specifically regulates the activity of auxin co-receptors involved in lateral root formation.

## Introduction

Auxins are a class of phytohormone involved in a large number of processes related to growth and development, such as cell division, cell growth, root development, leaf formation, apical dominance, vasculature differentiation, or fruit development among other processes (Gallei et al. 2020). In Arabidopsis (*Arabidopsis thaliana*) plants, auxin plays an essential role in lateral root (LR) formation and emergence (Xuan et al. 2015; Du and Scheres 2018). The root clock in Arabidopsis integrates an oscillating gene expression and auxin signaling to, approximately every 6 h (h), specify a new prebranch site (PBS), which marks the region competent for the future formation of a lateral root primordia (LRP) (Moreno-Risueno et al. 2010; Kircher and Schopfer 2018; Xuan et al. 2020). This oscillatory gene expression generates two opposite tendencies, one is in phase with the expression of the auxin-response marker that contains a synthetic auxin-responsive promoter called DR5 fused to the *LUCIFERASE* (DR5::LUC) and the other one is in antiphase (Moreno-Risueno et al. 2010). The *AUXIN RESPONSE FACTOR 7* (*ARF7*) and *INDOLE-3-ACETIC ACID 18* (*IAA18*)*/POTENT* transcription factors control this oscillatory circuit to determine PBS (Perianez-Rodriguez et al. 2021). Furthermore, based on the epistatic effect of *iaa28-1* mutation, a mutation in the DII domain causing auxin insensitivity, on IAA18/ARF7-mediated PBS spacing, it has been proposed that IAA28 signaling might control the amplitude of the oscillations by altering auxin responses necessary for the formation of PBS (Perianez-Rodriguez et al. 2021). IAA28 also controls founder cell (FC) specification through the IAA28-GATA23 module (De Rybel et al. 2010). Later, and upwards from the basal meristem, FC at the PBS are activated to initiate nuclei migration and anticlinal cell division, forming LRP. This initiation process requires the activity of *IAA14/SOLITARY-ROOT* (*SLR*), which is degraded in an auxin depended manner (Guseman et al. 2015). As the primordium develops, it eventually breaks through the overlaying tissues, as an emerged LR (eLR) (Casimiro et al. 2003; De Rybel et al. 2010; Manzano et al. 2014).

Auxin facilitates and stabilizes the interaction between the TRANSPORT INHIBITOR RESPONSE 1 (TIR1) or AUXIN SIGNALING F-BOX (AFBs) family proteins and the Auxin/Indole-3-Acetic Acid (Aux/IAA) proteins to form an auxin co-receptor system (Tan et al. 2007). The crystal structure of the co-receptor revealed that auxin binds to a pocket in TIR1 to extend the Aux/IAA-contact surface, increasing and stabilizing their interaction (Tan et al. 2007). Once the co-receptor is assembled, Aux/IAA proteins are ubiquitinylated and subsequently degraded through the proteasome 26S (Gray et al. 2001; Dos Santos Maraschin et al. 2009). Aux/IAA degradation releases the ARF transcription factors to induce the auxin-depend signaling (Parry and Estelle 2006). During land plant evolution, the TIR1/AFBs and Aux/IAA gene family has expanded, with 6 and 29 members, respectively, in Arabidopsis (Parry et al. 2009; Luo et al. 2018). It has been proposed that the different ubiquitin-ligase (E3) complex (Cullin, F-box containing complex, SCF) SCF^TIR1/AFB^-Aux/IAA co-receptors, which can be simultaneously co-expressed in specific cell types or organs, might read a broad range of auxin concentrations to give specific auxin responses (Calderon Villalobos et al. 2012). Recent works have shed light on the diverse molecular mechanisms used by plant to trigger auxin responses. Auxin-trigged transcriptional responses mainly rely on the canonical SCF-TIR1/AFB-Aux/IAA-ARF signaling in the nucleus; while those rapid responses are usually regulated by nontranscriptional pathways, for instance the cytosolic SCFTIR/AFB-CNGC14 (the plasma-membrane-localized Ca^2+^ channel) pathway to mediate calcium flux and the cell surface AUXIN-BINDING PROTEIN 1 (ABP1)-TRANSMEMBRANE KINASE 1 (TMK1) signaling to induce ROP GTPase activation, cell wall acidification, and protein phosphorylation (Dindas et al. 2018; Powers and Strader 2020; Friml et al. 2022). In addition, TIR1/AFB proteins also have an adenylate cyclase (AC) activity that generates cAMP, a secondary metabolite that seems to participate in the auxin signaling (Qi et al. 2022). This AC activity is needed for root growth inhibition in response to auxin or gravitropism, but not for triggering calcium transient peaks needed for the rapid auxin-induced apoplast alkalinization and membrane depolarization. Recently, two proteins, ABP1-LIKE PROTEIN 1 (ABL1) and ABL2, have been shown to interact with TMKs in an auxin-dependent manner and seems to act as extracellular auxin co-receptors, showing overlapping and distinct functions with ABP1 (Yu et al. 2023). Despite these advances made in auxin perception some questions are still open, as for example how the specific recognition between TIR1/AFBs and the Aux/IAA is regulated.

Plants synthesized many secondary metabolites that regulate plant growth or responses to external environmental changes. The indole scaffold serves as a building block for a multitude of natural compounds and is considered as one of the most used heterocyclic motifs (Cna’ani et al. 2018), including the phytohormone auxin (Zhao 2010; Kitajima and Takayama 2016). Bioactive 1-indole 3-acetic acid (IAA) levels can be regulated by conjugation with amino acids, sugars or by methylation (Bajguz and Piotrowska 2009). Another important IAA-conjugate is the methyl 1*H*-indole 3-acetate (MeIAA), which was thought to be an inactive form that needs an esterase to release the active IAA (Yang et al. 2008). However, MeIAA seems to have a more specific role in inhibiting hypocotyl elongation or altering the root system architecture (Li et al. 2008; Ludwig-Müller 2011; Abbas et al. 2018). Different agonists and antagonists have been used to elucidate auxin signaling components and to understand how the interaction between TIR1 and a specific subset of Aux/IAA proteins is promoted (Hayashi et al. 2008a, 2008b; Vain et al. 2019).

In this work, using ultrahigh liquid chromatography–electrospray ionization mass spectrometry (UHPLC–ESI–QTOF–MS), we found a peak that accumulates in illuminated roots of Arabidopsis and inferred a possible formula that might correspond to bis-methyl auxin conjugated with a *N*-glucoside, called here BiAux. Following a previously published protocol (Chisholm and Van Vranken 1995), we synthesized this compound and demonstrated that it has bioactivity in plants. Treatment of Arabidopsis seedlings with BiAux specifically increases the number of PBS and eLR without substantially modifying the primary root growth or shoot development. Transcriptomic analyses show that BiAux induces the expression of a set of genes that are also regulated by auxin, and a set of specific genes, which includes genes related to root development. Remarkably, this BiAux regulation seems to be depended on *ARF7*, but not *ARF19* activity. BiAux modifies the auxin signaling by enhancing the formation of specific auxin co-receptor systems related to LR formation. Molecular docking analyses show that BiAux binds into the TIR1 solenoid, between IAA and inositol-6-phosphate (IP6) and generates different electrostatic potential on TIR1 and AFB2 from those on AFB1 or AFB3. In addition, genetic functional analyses show that TIR1 and AFB2 activities, but not AFB1 or AFB3, are needed for BiAux function. In agreement with these observations, BiAux enhances the interaction of TIR1 and AFB2 with IAA28 and IAA18/POTENT, two regulators of FC specification. Using confocal microscopy, we show that BiAux, when combined with auxin, enhances IAA28 protein degradation in the root meristem. Taken together our data support that BiAux enhances, in an auxin-dependent manner, the formation of specific auxin co-receptor systems to activate downstream signaling and exert a fine-tune regulation on a set of auxin-related genes involved in LR formation.

## Results

### BiAux regulates LR formation

**Figure 1.**
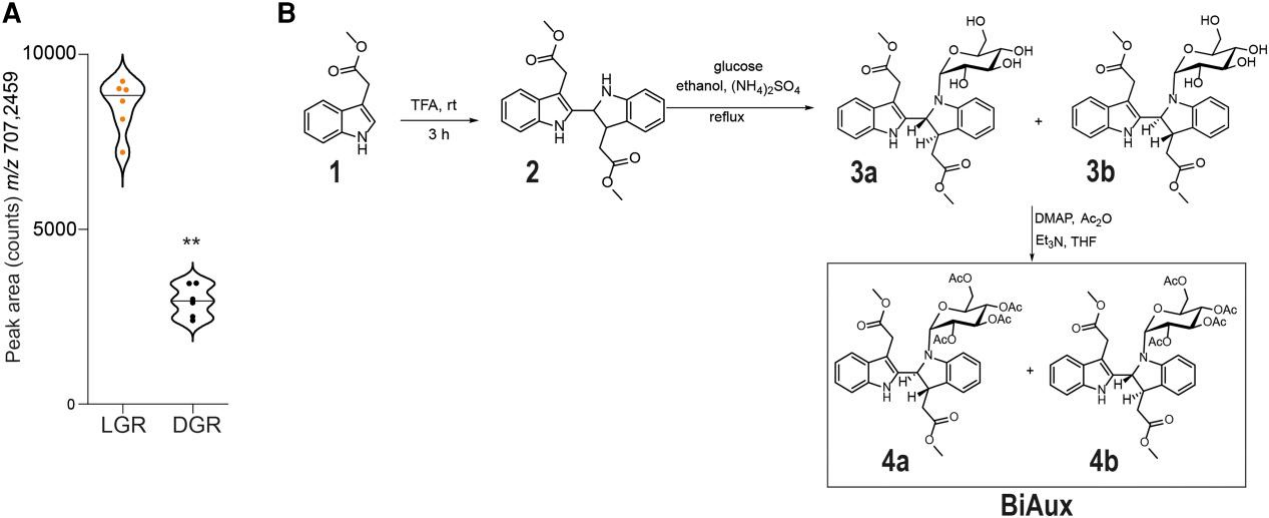
Light grown roots accumulate higher level of BiAux. **A)** Area (counts) of the peak corresponding to the metabolite *m/z* 707.2451 in light-grown roots (LGR) or dark-grown roots (DGR). Asterisks indicate the statistical significance by a *t*-test **P-value < 0.01. *n* = 6. **B)** Schematic procedure for BiAux synthesis process (for additional details see Supplementary Materials and methods). Notice that two diasteroisomers were obtained: 3a, 3b, which render the final BiAux diasteroisomers 4a and 4b.

Previously, we have shown that root illumination promotes LR formation while affecting gene expression and metabolite accumulation, such as flavonoids (Silva-Navas et al. 2015, 2016). Here, we wanted to study the accumulation of unknown metabolites in light-grown roots (LGR). Among several peaks with increased levels (Silva-Navas et al. 2016), we focused on the one corresponding to the metabolite of LC-ESI(+)-MS *m/z* of 707.2459 because it was significant and consistently accumulated by the effect of root illumination in all analyzed replicates (Fig. 1A). As we only had the exact mass, we searched for molecules that matched this mass using the chemical software ChemCalc (https://www.chemcalc.org/). All the molecular formulas retrieved (Supplementary Table S1) were analyzed in ChemSpider web site (http://www.chemspider.com/), through which we found that only four out of them matched with already known molecules. Among these four candidates, we noticed a compound that contained two auxin molecules (C_36_H_38_N_2_O_13,_ 3,3′-bis(2-methoxy-2-oxoethyl)-1-(2,3,4,6-tetra-*O*-acetyl-β-D-glucopyranosyl)-1*H*,1′*H*-2,2′-bisindole) and this compound was previously synthesized in vitro (Chisholm and Van Vranken 1995). As auxin regulates root growth and LR formation, the processes modified by root illumination, we decided to focus on this compound. We synthesized this compound (C707.24) following four steps: (i) dimerization; (ii) glycosylation; (iii) oxidation of the β-glucoside; (iv) finally acetylation of the sugar (Supplementary Fig. S1A and Materials and methods). To simplify its biosynthesis, we carried out a more sustainable process using dimethylaminopyridine instead of 2,3-dichloro-5,6-dicyano-1,4-benzoquinone (Fig. 1B; Supplementary Fig. S1A and Materials and methods). Using this approach, we obtained higher yield of a final product that was reduced in position C2 and C3 of the bisindol and showed a LC–ESI(+)–MS *m/z* of 709.2686 (Supplementary Fig. S1B and Materials and methods). We, therefore, termed this product as BiAux. The purity of the compound was of 99% and insubstantial traces of the precursors, except for the dimer, were detected. The presence of the product was confirmed by ^1^H NMR and ^13^C NMR and LC–MS/MS (see data in Supplementary Materials and methods). Finally, it is worth mentioning that two diasteroisomers were obtained: 3a, 3b, 4a, and 4b (Fig. 1B and Supplementary Materials and methods). This was confirmed by LC–ESI–QTOF–MS analyses and the results showed two peaks that correspond to diasteroisomers 4a and 4b, (Supplementary Fig. S1B).

Next, we tested whether the C707.24 and BiAux have similar biological activities. We treated SKP2Bp::GUS Arabidopsis seedlings, a maker line that labels LRFC, LRP, and eLRs with C707.24 or BiAux. Both compounds significantly increased the number of SKP2Bp::GUS expression sites (SKP2BES) (Supplementary Fig. S1, C and D), without reducing the length of the primary root as auxin did (Supplementary Fig. S1E). We also found that BiAux synthesis intermediates did not increase the number of SKP2BES or eLR nor the SKP2Bp::GUS expression (Supplementary Fig. S2, A to C). As BiAux synthesis was simpler than C707.24, we decided to use this compound for further experiments. Furthermore, BiAux treatment specifically increased *SKP2B* expression mainly inside of FC and LRP, while auxin increased its expression along the entire pericycle and vasculature (Supplementary Fig. S2D). Next, seedlings harboring DR5::LUC, a marker used to reflect lateral root oscillatory activities, were cultivated in LGR or dark-grown root (DGR) conditions (Silva-Navas et al. 2015) for 4 d and then they were treated with mock or BiAux. We found that BiAux-treated DGR seedlings showed more DR5::LUC expression sites (DR5ES) along the main root than the BiAux-treated LGR seedlings (Supplementary Fig. S2E), although we cannot discard that root illumination favor LRP arrest that do not express DR5.

It is possible that BiAux-associated phenotype could be due to free IAA originated from BiAux degradation. To answer this, we compared the capacity of inducing PBS after treatment with different IAA concentrations or BiAux. First, we found that BiAux increased the number of DR5ES at 0.25 *µ*M, and this effect increased in a dose-dependent manner (Supplementary Fig. S3A). Next, to analyze whether auxin produces the same effect, we treated DR5::LUC seedlings with increasing concentrations of IAA. As shown in Supplementary Fig. S3, B and C, none of the IAA concentrations mimicked the BiAux-treated phenotype in terms of root length or the spacing of DR5ES. Although we cannot rule out the possibility that BiAux can be modified inside of a specific subset of plants cells, our data suggest that the entire BiAux molecule might be specifically required to promote the formation of PBSs and eLRs, possibly by modulating the root clock activity.

### BiAux regulates lateral root formation

**Figure 2.**
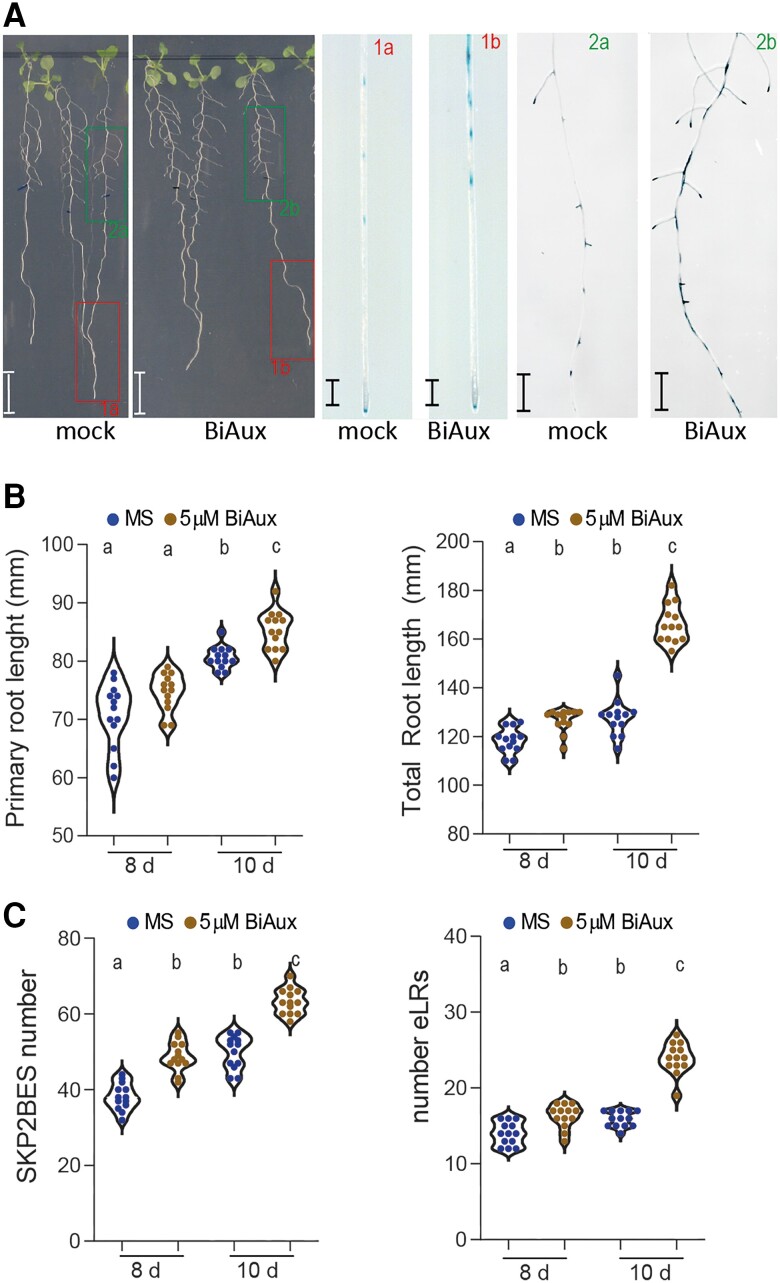
BiAux induces lateral root formation. **A)** Arabidopsis SKP2Bp::GUS seedlings grown in ½MS medium for 4 d and then transferred to new plates containing mock or 5 *µ*M of BiAux during 6 d. Right panels show the GUS-stained roots labeled as 1a and 1b (apical region of primary root) or 2a and 2b (shootwards part of the root). Numbers indicate the position of the pictures in the GUS-stained roots. Scale bar corresponds to 0.5 cm. **B)** Primary root length and total root length (root length of the primary root plus eLRs) of SKP2Bp::GUS seedlings grown or 4 d and then transferred to new plates containing mock or 5 *µ*M of BiAux during 4 or 6 d, *n* ≥ 20. **C)** Number of SKP2BES (left) or eLR (right) in SKP2Bp::GUS seedlings grown as in B. *n* ≥ 20 (two biological replicates). Significance was analyzed by ANOVA and Tukey HSD post-test. Different letters indicate statistical differences.

Arabidopsis seedlings treated with BiAux showed a slightly longer primary root and a significant longer root system (total root length of the primary root plus eLRs) (Fig. 2, A and B). We observed that BiAux treatment increased the number of cells in the root meristem but reduced the length of meristematic cells. Therefore, the meristem size between the BiAux- and mock-treated roots was similar (Supplementary Fig. S4). In addition, we found that BiAux-treated seedlings developed a significantly higher number of SKP2BES and eLR than the number in nontreated seedlings (Fig. 2C).

### BiAux alters the root clock and auxin signaling

**Figure 3.**
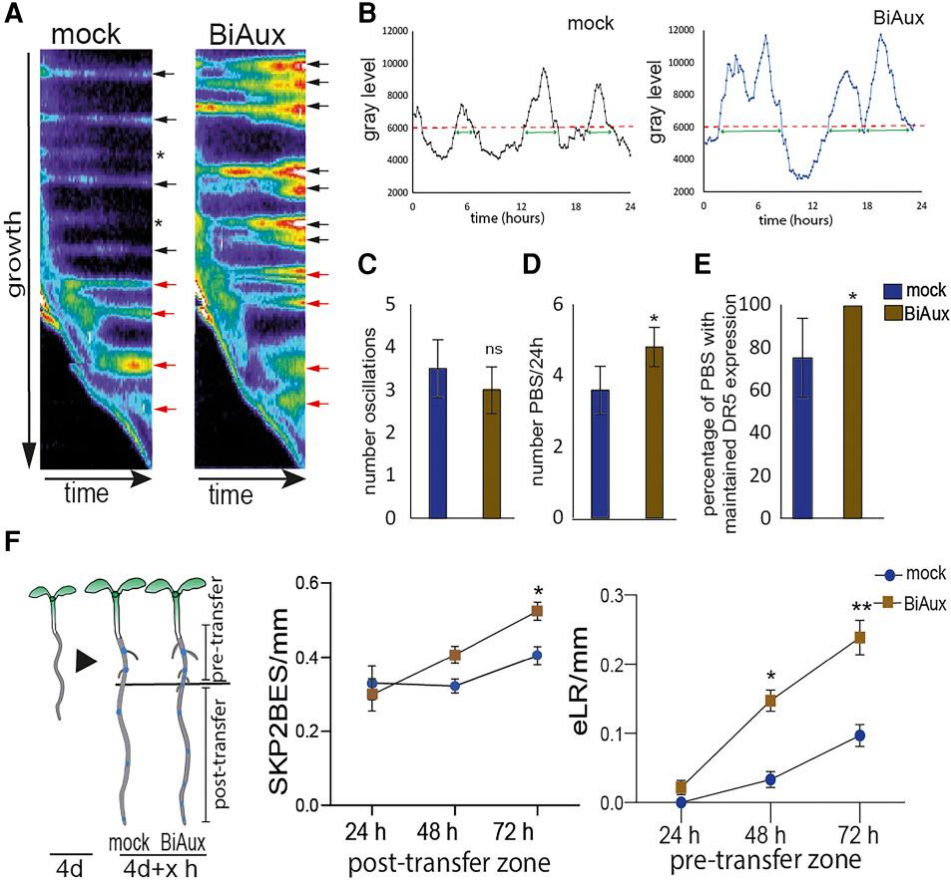
BiAux modifies the root clock. **A)** Kymographs of DR5::LUC luminescence showing PBS production and DR5:LUC signal intensity in mock- and BiAux-treated seedlings. Red arrows indicate priming events in a 24-h period. Black arrows indicate PBS already generated. Note that the DR5::LUC expression is maintained in all PBS over time in BiAux treated roots, while its expression is not maintained in all PBS (asterisks) over time in mock-treated roots. **B)** LUC activity measurements at the root tip registering the oscillations during 24 h. Dash lines indicate the threshold of the oscillation. Arrow lines indicate the length of the oscillations. **C)** Average of DR5::LUC oscillations during 24 h in mock- or BiAux-treated seedlings. ns, statistical significance by a *t*-test *P*-value > 0.05. **D)** Number of PBS specified in mock- or BiAux-treated roots per 24 h. **E)** Percentage of PBS that maintained the DR5::LUC signal over the time. *, significant differences by *t*-test *P* < 0.05. Number of roots analyzed in **(C)** to **(E)** = 12 for mock and 5 for BiAux. **F)** SKP2Bp::GUS seedlings were grown in ½MS for 4 d and then, transferred to fresh medium containing mock or 5 *µ*M BiAux for the indicated hours. Afterwards they were stained for GUS activity in the plate and SKP2BES in the post-transfer root section or number of eLRs in the pre-transfer section were quantified. *n* > 200 seedlings in each point (one biological replicate). Asterisks indicate the statistical significance by a *t*-test *, *P*-value < 0.05, ***, *P*-value < 0.001. In all cases, error bars correspond to standard deviation (Sd).

As BiAux specifically increased the number of DR5ES, we decided to analyze whether it affects the root clock, which regulates PBS formation (Moreno-Risueno et al. 2010). Five-day-old DR5::LUC seedlings were transferred to fresh plates containing mock or 5 *µ*M of BiAux for 48 h. Afterwards, the luminescence in the root tip was recorded for 24 h. Kymograph analyses showed that in both mock- and BiAux-treated roots, DR5::LUC oscillated about four times per every 24 h while in BiAux-treated the number of oscillations was not significantly different (Fig. 3, A to C). However, in BiAux-treated seedlings, LUC activities in the oscillations were generally higher than the activities detected in mock-treated seedlings (Fig. 3, A and B), suggesting an increased amplitude of the oscillations. Corresponding with these alterations of the LUC activities in the oscillation zone (OZ), more and closer PBS were observed in BiAux-treated roots (Fig. 3, A and D). It should be noted that all PBS specified in BiAux-treated seedlings maintained higher expression of DR5::LUC over the time while in mock-treated roots about 25% of the PBS lost the DR5::LUC expression (Fig. 3, A and E). Taken together, these data indicate that BiAux increased the amplitude of the oscillations. As a result, more pericycle cells might be primed. Furthermore, BiAux maintains the expression of DR5 at high levels, favoring the formation of FC and LR. Conversely, in mock-treated roots some PBS loss DR5 expression and become “PBS-reservoirs” or “arrested-PBSs”, from which no LR are develop under normal conditions.

Next, we tested whether BiAux enhances the formation of FC and the emergence of LR. We analyzed 4-d-old SKP2Bp::GUS seedlings that were transferred to a medium containing mock or 5 *µ*M of BiAux for 24, 48, or 72 h. Afterwards, roots were directly stained for GUS activity in the cultivation plate to trace the transference point. We found that the number of SKP2BES in the root portion growing in presence of BiAux (post-transfer zone) was significantly higher than in the mock treatment after 72 h (Fig. 3F), indicating that BiAux favors FC specification. We also observed that BiAux treatment significantly increased the number of eLR in the pretransfer root zone (Fig. 3F) after 48 h, suggesting that it promotes the development of previously specified but arrested FCs and/or LRP (Manzano et al. 2014). Taken together, these data indicate that BiAux promotes FC specification and LR development.

**Figure 4.**
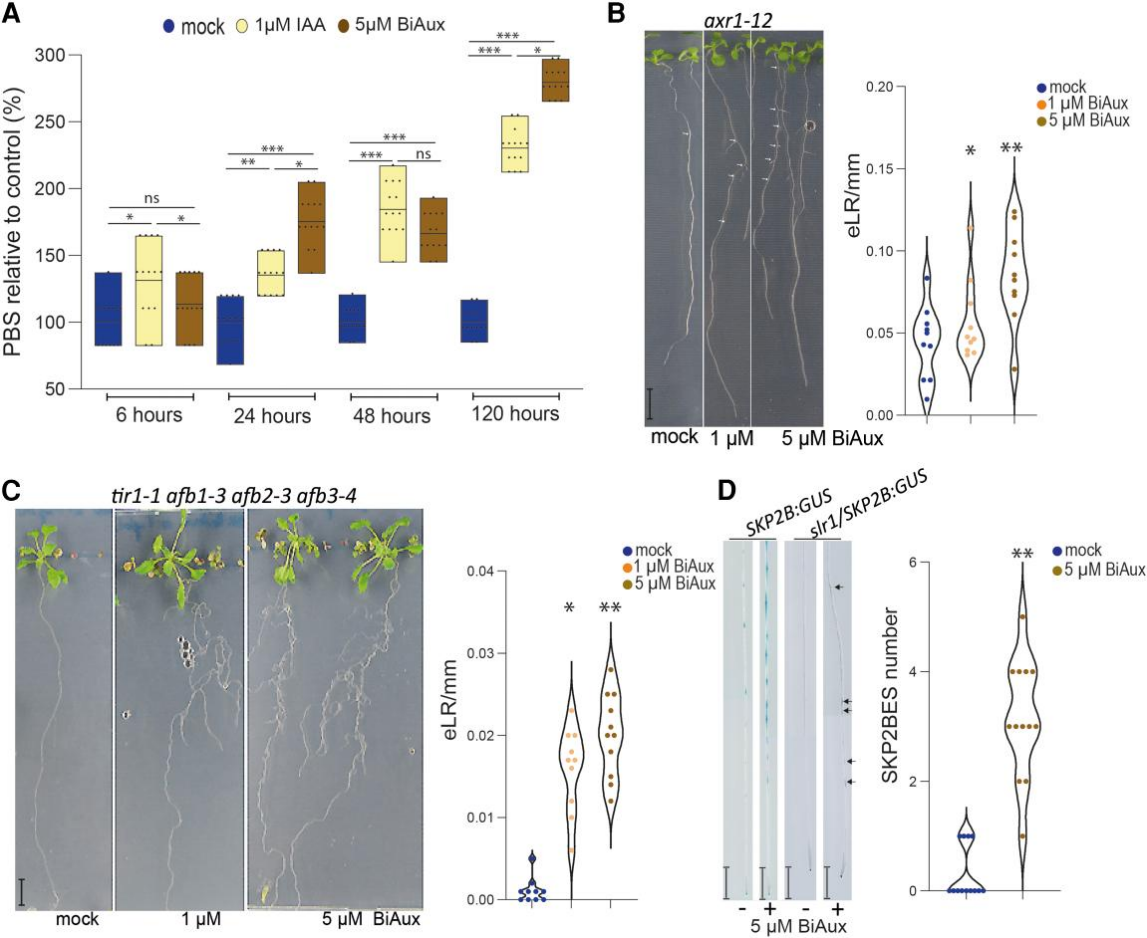
BiAux affects auxin responses. **A)** Relative number of DR5ES in seedlings grown 4 d in ½MS and the indicated hours in ½MS with DMSO (mock), 1 *µ*M of IAA or 5 *µ*M BiAux for the indicated time. Values represent the percentage relative to the mock average. Asterisks indicate significant differences by *t*-test in a genotype comparing mock and BiAux treatment. *, *P* < 0.05; **, *P* < 0.01; ***, *P* < 0.001 by *t*-test, *n* ≥ 15 (one biological replicate). Limits in the boxplot correspond to maximum and minimum values. Line inside of boxplot indicates the main. **B)** Phenotype of *axr1-12* mutant seedlings grown in ½MS with mock or with 1 *µ*M or 5 *µ*M of BiAux for 10 d. Scale corresponds to bar: 1 cm. Right graph shows the density (number/mm) of eLR. *n* = 10 (one biological replicate). Asterisks indicate significant differences in the *axr1-12* by BiAux treatment by *t*-test. *, *P* < 0.05; **, *P* < 0.01. **C)** Phenotype of *tir1 afb1 afb2 afb3* quadruple mutant, which developed the main root, grown in ½MS or with mock or 1 or 5 *µ*M BiAux for 21 d. Scale corresponds to bar: 1 cm. Right graph shows the density (number/mm) of eLR. *n* ≥ 8 (two biological replicates). Asterisks indicate significant differences in the quadruple mutant by BiAux treatment by *t*-test. *P < 0.05; **P < 0.01. **D)** GUS staining of SKP2Bp::GUS and *slr-1/*SKP2Bp::GUS roots grown in ½MS with mock (−) or 5 *µ*M of BiAux (+) during 12 d. Scale bar corresponds to 0.5 cm, *n* ≥ 12. Arrows indicate SKP2BES in BiAux-treated *slr* roots. Note that each root is a composite figure. Asterisks indicate significant differences in *slr1* by BiAux treatment by *t*-test. **P < 0.01.

As auxin participates in LR formation, we decided to analyze the effect of BiAux in auxin signaling mutants. First, we found that, compared to the mock treatment, BiAux treatment increased the number of DR5ES in wild-type roots to a similar level observed in IAA-treated roots up to 48 h after the treatment, and to a higher level than the IAA treatment at 120 h (Fig. 4A). Next, we analyzed the effect of BiAux in auxin-response mutants defective in LR formation, such as *auxin-resistant 1* (*axr1-12)*, has fewer eLRs (Timpte et al. 1995), the quadruple auxin receptor mutant *tir1/afb1/afb2/afb3*, which barely develops the primary root in those developed ones, no eLRs are observed (Prigge et al. 2020), and *solitary root-1* (*slr-1*), which does not produce LRP (Fukaki et al. 2002). We found that BiAux treatment increased the density of eLR in *axr1-12* and in *tir1/afb1/afb2/afb3* seedlings in a dose-dependent manner (Fig. 4, B and C). In the case of *slr-1*, BiAux did not lead to formation of emerged LRs, but significantly increased the number of SKP2BES (Fig. 4D), indicating that this compound promotes the formation of FC in this mutant.

### BiAux alters gene transcription

**Figure 5.**
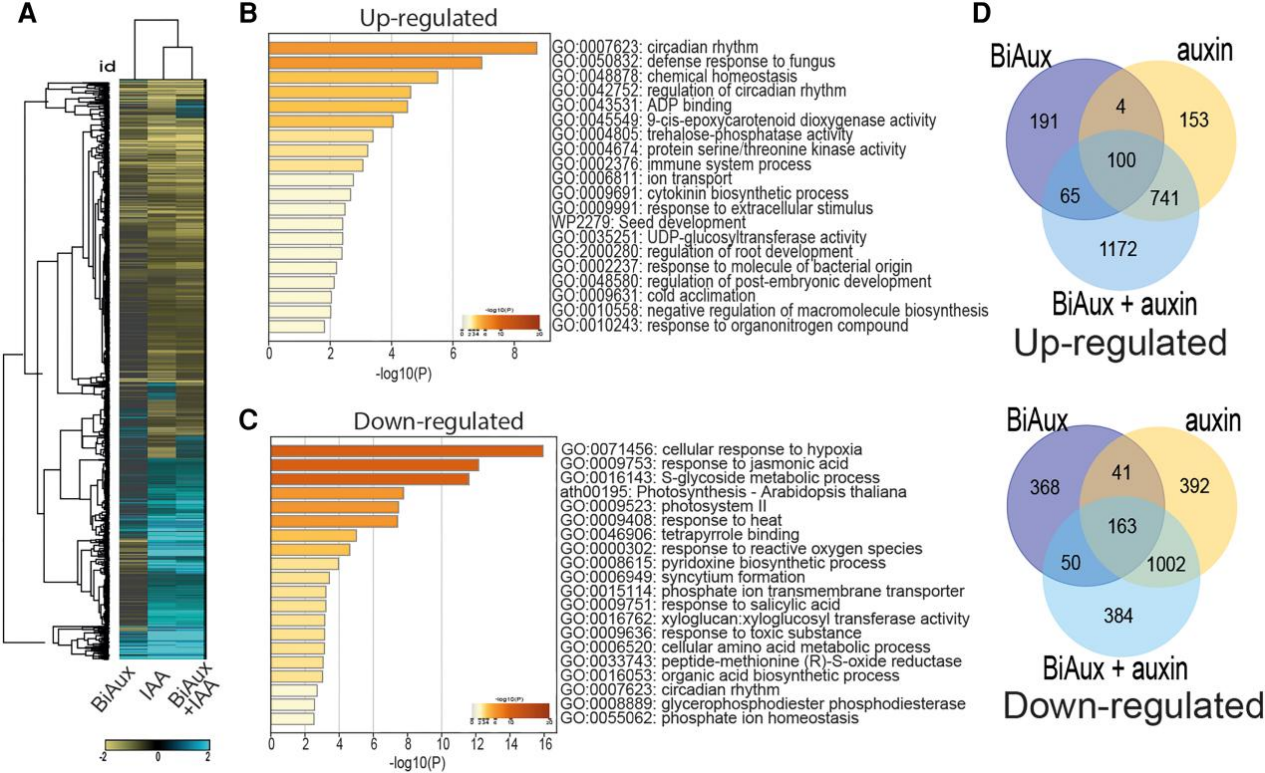
BiAux alters gene expression. **A)** Hierarchical clustering of genes deregulated by BiAux, IAA or the combination of both (IAA + BiAux). Scale bar indicates gene expression level compared with mock (log_2_). **B, C)** GO of upregulated **(B)** or downregulated **(C)** genes in Arabidopsis roots treated with mock or BiAux during 3 d. Numbers indicate the probability (log_10_ of the FDR). **D)** Venn diagrams showing the common genes between upregulated or downregulated genes in BiAux-, IAA- or BiAux + IAA-treated seedlings. Number of common genes between BiAux and IAA (both up- and downregulated) were statistically significant (binomial test, *P*-value < 0.01).

Our data indicate that BiAux might promote the auxin signaling related to PBS formation, FC specification, and LR development. To understand the role of BiAux at the molecular level, we carried out comparative transcriptomic analyses. In comparison with IAA supplementation, BiAux treatment modified the expression of a lower number of genes in roots. BiAux increased the expression of 360 genes and reduced the expression of 622 genes (Fig. 5A; Supplementary Table S2, sheet 1). Gene ontology (GO) analyses of upregulated genes showed an enrichment of genes belonging to regulation of root development or ion transport among others (Fig. 5B), while downregulated genes showed a significant enrichment in transcripts related to cellular response to hypoxia, glucosinolate biosynthesis, jasmonic acid, response to heat, or phosphate ion transport among others (Fig. 5C). Next, we compared the transcript accumulation in the roots in response to the supplementation with BiAux, auxin (IAA) or both simultaneously. We found that although BiAux and IAA deregulated a significant set of common genes, there was still a group of genes solely respond to the BiAux treatment (Fig. 5D). Interestingly, GO category of root development was found by analyzing the upregulated gene list form this group (Fig. 5B; Supplementary Fig. S5A), suggesting that Arabidopsis root may differentially respond to the BiAux treatment from the IAA treatment. This may explain the distinct phenotypes observed between the BiAux- and IAA-treated roots (Supplementary Fig. S3). Next, we treated Arabidopsis seedlings with BiAux plus a low amount of IAA (10 nM). This double treatment deregulated a set of genes almost similar to the one from the IAA treatment alone, but the addition of BiAux altered (increased or decreased) their expression levels (Fig. 5A; Supplementary Table S2). Furthermore, clustering analyses identified two clusters of genes (number 4 and number 8), the upregulation of which was enhanced in the BiAux + IAA treatment, compared to the level in the IAA treatment alone (Supplementary Fig. S5B). In both clusters, an enrichment in genes related to hormone signaling and isoprenoid biosynthesis was found, suggesting that BiAux may boost the endogenous IAA response. Taken together, these results show that BiAux regulates a specific set of genes that are not controlled by IAA and, in addition, enhances the response of IAA-regulated genes, likely through activation of auxin-dependent pathways.

### BiAux activity is mediated by TIR1 and AFB2

**Figure 6.**
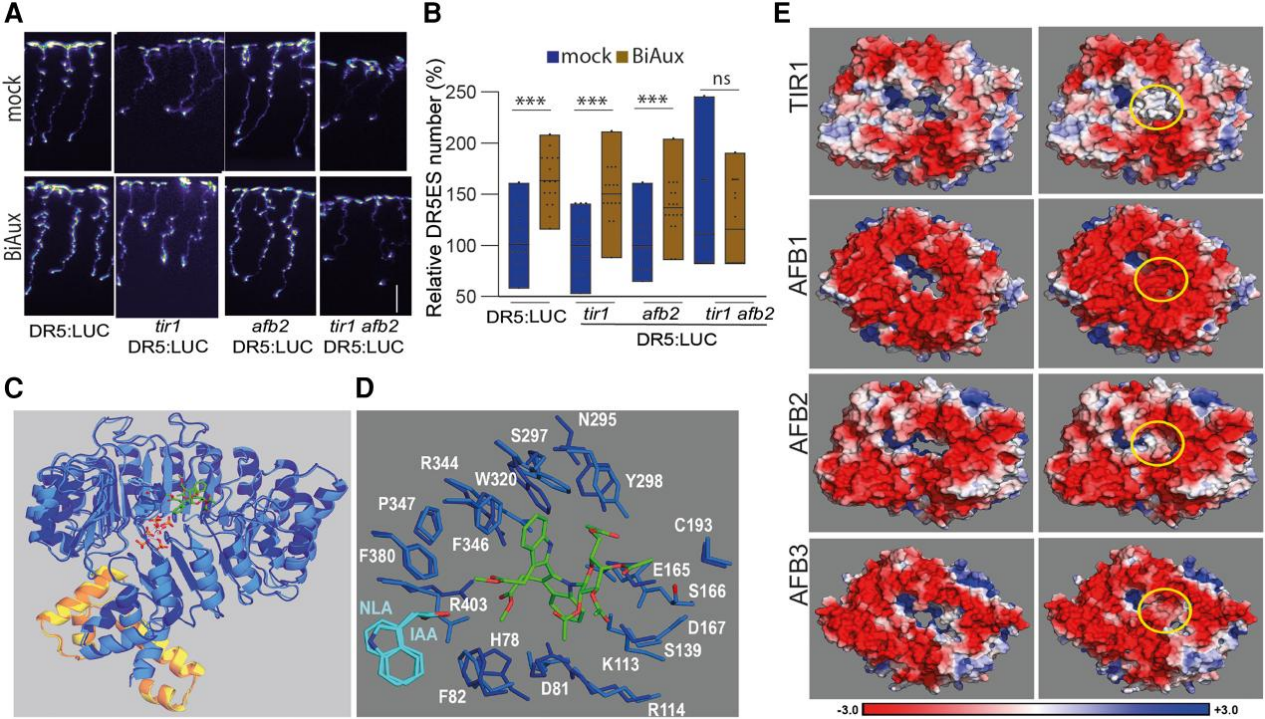
BiAux signaling requires TIR1 and AFB2. **A)** Representative pictures of luciferase signal from DR5::LUC reporter in control seedlings, *tir1*, *afb2 or* double mutant *tir1 afb2* grown for 4 d in ½MS and then transferred to fresh medium containing DMSO (mock) or 5 *µ*M of BiAux for 4 d. Scale bar = 1 cm. **B)** Relative number of DR5ES (BiAux/mock) measured in the whole roots of control and in *tir1*, *afb2*, and double *tir1/afb2*. Values represent the percentage relative to the mock average, *n* ≥ 15 (three biological replicates). Asterisks indicate significant differences by *t*-test in a genotype comparing mock and BiAux treatment. ***, *P*-value < 0.01. ns, no significant. Limits in the boxplot correspond to maximum and minimum values. Line inside of boxplot indicates the main value. **C)** Superposition of the crystal structure of the TIR1 (dark blue ribbon)–ASK1 (orange ribbon)–IP6 (sticks with carbons in white) complex (PDB id. 2P1M) and the optimized structure of the TIR1 (light blue ribbon)–ASK1 (yellow ribbon)–BiAux (sticks with carbons in green) complex. The location of auxin (sticks with carbons in cyan) and IP6 (orange and red) from the crystal structures of 2P1P (PDB id.) are also shown for reference. **D)** Binding site defined by a neighborhood of 4 Å around BiAux (sticks with carbons in green) in the superposition of crystal (sticks with carbons in dark blue) and optimized (sticks with carbons in light blue) structures displayed in **(C)**. The location of auxin (IAA) and naphthalene (NLA, naphthalen-1-yl-acetic acid) are also included for reference. **E)** PB-EP mapped onto the molecular surface of TIR1, AFB1, AFB2, and AFB3 proteins viewed from the protein side of the BiAux-binding site. Left images show the proteins in the absence of BiAux and right images show the BiAux complexes with the PB-EP mapped onto the surface of BiAux, which is marked with yellow circles. The scale bar on the bottom of this panel indicates the range of PB-EP values (in *kT*/*e* units) used in these images.

Based on the phenotype of BiAux-treated plants and the fact that BiAux contains an auxin structure, it would be possible that BiAux affects to auxin signaling by modifying the activity of the auxin receptors. To investigate this, we analyzed the response of DR5::LUC marker in wild type, *tir1*, *afb1*, *afb2*, and *afb3* mutants. We found that BiAux treatment significantly increased the number of DR5ES in wild type and the single mutants (Fig. 6, A and B). We only found a significant reduction of DR5ES number in response to BiAux treatment in the double *tir1 afb2* mutant or higher order mutants that include this combination (Fig. 6, A and B; Supplementary Fig. S6). These data indicate that TIR1 and AFB2 activities are needed for BiAux function.

To study whether BiAux can bind to TIR1 or AFBs we used molecular modeling and docking calculations to obtain local geometries and estimate the binding energies (Supplementary Fig. S7, A and B). These docking analyses showed that BiAux bounds with low energy in the center of the TIR1-Lucine Rich Repeats solenoid, near the binding sites of IAA and IP6 molecules but far from the cAMP catalytic site (Qi et al. 2022) (Fig. 6C). We also identified a set of residues in the auxin binding site that were closer than 4 Å from BiAux (Fig. 6D). Some of these residues have been implicated in auxin and InsP6 binding (His78, Arg403) or in supporting the floor of the auxin-binding pocket being located around the InsP6 binding pocket (Lys113, Arg114) (Tan et al. 2007). Mutations affecting these residues reduced the interaction between TIR1 and Aux/IAA7 (Calderon Villalobos et al. 2012). Therefore, we hypothesized that BiAux stabilizes TIR1/AFB–Aux/IAA interaction to increase auxin perception and signaling. Based on docking models, BiAux binding generated a neutral electrostatic potential around the binding site in TIR1 and AFB2 (Fig. 6E). However, this potential was negative in the case of AFB1 or AFB3 (Fig. 6E). To analyze whether BiAux acts on other plant species, we decided to analyze its effect on tomato, and found that BiAux treatment increased root growth (Supplementary Fig. S7C), suggesting that this compound is functional in other species. By blast analyses, we found tomato sequences with high similarity to Arabidopsis TIR1 and AFB proteins (Supplementary Fig. S7D) and also structural similitudes (Supplementary Fig. S7E). Docking calculations identified a BiAux-binding site in the tomato proteins similar to the one found in Arabidopsis TIR and AFBs proteins (Supplementary Fig. S7F). Furthermore, the estimated affinity energies Δ*G* were almost identical for tomato TIR1 and slightly higher for the TIR1-like compared with the Arabidopsis proteins (−7.2, −6.9, −7.2, and −7.4 for TIR1, AFB1, AFB2, and AFB3, respectively, in Arabidopsis, and −7.3, −6.5, −6.9, and −6.9 in tomato TIR1 and TIR1-like; Supplementary Fig. S7A). Interestingly, similarly to the Arabidopsis proteins, we found that BiAux generated a neutral surface electrostatic potential in the binding site of tomato TIR1 and TIR1-like_2 while this potential is highly negative in TIR1-like_1 and TIR1-like_3 (Supplementary Fig. S7F). Taken together, these data open the possibility that BiAux might regulate the activity of auxin receptors in other plant species.

### BiAux function requires *ARF7* but not *ARF19*

**Figure 7.**
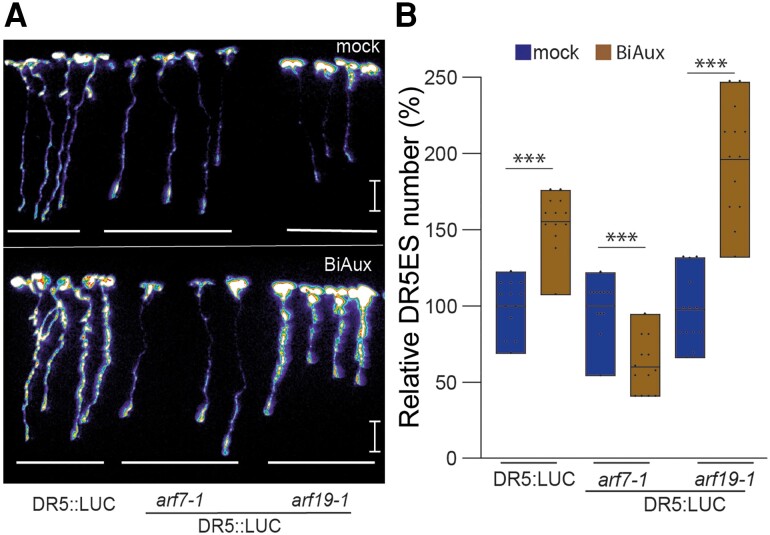
BiAux function requires *ARF7* activity. **A)** Representative pictures of luciferase signal in control seedlings, *arf7-1*, *arf19-1* grown for 4 d in ½MS and then transferred to ½MS containing DMSO (mock) or 5 *µ*M of BiAux for 4 d. Scale bar = 1 cm. **B)** Relative number of DR5ES (BiAux/mock) measured in the whole roots of control and in DR5::LUC, *arf7-1* DR5::LUC, or *arf19-1* DR5::LUC seedlings. *n* = 8 (two biological replicates). Values represent the percentage relative to the mock average. Asterisks indicate significant differences by *t*-test in a genotype comparing mock and BiAux treatment. ***, *P*-value < 0.01. Limits in the boxplot correspond to maximum and minimum values. Line inside of boxplot indicates the main value.

As BiAux regulates LR formation, we decided to analyze the BiAux upregulated genes using the Visual LRTC tool to identify genes that could be involved in LR formation (Parizot et al. 2010). We found that a large number of BiAux upregulated genes were also induced by auxin during LR initiation (Vanneste et al. 2005) or in the whole plant (Okushima et al. 2005) (Supplementary Table S3). Interestingly, many of these genes were not induced, or to a lower extend, in the *arf7* mutant, while their expression in *arf19* was similar to wild-type seedlings (Supplementary Fig. S8A, Supplementary Table S3). GO analysis of these BiAux upregulated and ARF7-dependent genes showed an overrepresentation of genes related to root morphogenesis among other categories (Supplementary Fig. S8B). These data suggest that BiAux function might be mediated through *ARF7*. To corroborate this, we analyzed the expression of DR5::LUC in *arf7* and *arf19* mutants after BiAux treatment. We found that *ARF7*, but not *ARF19*, was required to increase the number of DR5ES in response to BiAux (Fig. 7, A and B). Furthermore, we found that BiAux treatment seemed to increase the expression of *ARF7*, mainly in the mature region of the root (Supplementary Fig. S8, C to E), suggesting a possible function for BiAux in the *ARF7* pathway in the LR emergence (Okushima et al. 2007).

### BiAux regulates genes associated with root clock oscillation and FC formation

DR5::LUC expression oscillates in phase with a set of genes involved in PBS specification while *ARF7*, whose function drives PBS formation, accumulates in antiphase during the oscillations (Moreno-Risueno et al. 2010; Perianez-Rodriguez et al. 2021). Furthermore, we found a significant overlap between BiAux-regulated and in-phase expressed transcripts (Supplementary Fig. S9A), suggesting a connection between BiAux function and the root clock. It has been shown that IAA18/POTENT is required for FC specification and root clock regulation by interacting and sequestering ARF7 (Perianez-Rodriguez et al. 2021). Furthermore, *potent* mutant, an Aux/IAA domain mutant, and loss-of-function *arf7-1* mutant showed higher levels of DR5::LUC signals in the OZ and PBS number (Perianez-Rodriguez et al. 2021). The overlap between BiAux-regulated genes with *potent*- and *arf7*-regulated genes identified several common genes (Supplementary Fig. S9B). Interestingly, among these common genes, we identified *LATERAL ROOT BOUNDARY 29* (*LBD29*), which regulates cell division during LR development (Feng et al. 2012), or *PERICYCLE FACTOR TYPE-A 6* (*PFA6*), which is also involved in LR formation (Zhang et al. 2021). We found that BiAux treatment substantially increased the DR5::LUC signal and augmented the OZ in *potent* mutant, suggesting that the BiAux has the capacity of promoting auxin signaling in this mutant (Supplementary Fig. S9C). This would be in agreement with a role of IAA18/POTENT as a repressor of the oscillations to generate periodic responses (Perianez-Rodriguez et al. 2021).

IAA28-dependent auxin signaling regulates *GATA23* expression to specify LRFC (De Rybel et al. 2010). The *iaa28-1* mutant, which contains a mutation in the DII domain, generates a nondegradable version of the IAA28 protein (Rogg et al. 2001). We observed that both wild type and *iaa28-1* mutant increased the number of DR5ES in response to BiAux treatment (Supplementary Fig. S10, A and B), although it was significantly smaller in the mutant (2.3-fold versus 1.7-fold), suggesting that proper degradation of IAA28 is needed for BiAux function. In addition, we used the SKP2Bp::GUS marker to analyze the formation of LRFCs in the *iaa28-1* mutant roots in response to the BiAux treatment (Supplementary Fig. S10, C and D). Although *iaa28-1* mutant still responded to BiAux, the *iaa28-1* strongly reduced the number of SKP2BES compared with wild-type plants (Supplementary Fig. S10E). This data suggest that the formation of LRFCs promoted by BiAux depends on IAA28 degradation.

### BiAux regulates auxin co-receptor activity

Auxin is perceived by a co-receptor system involving the interaction between the TIR1/AFBs and Aux/IAA proteins. Thus, we decided to analyze whether BiAux affects auxin perception by using the auxin–receptor system developed in yeast (Calderon Villalobos et al. 2012) or by pull-down assays (Parry et al. 2009). We found that, opposite to auxin, BiAux alone did not promote the formation of auxin co-receptors (Supplementary Fig. S11, A to C). However, combined with IAA, BiAux increased the interaction between TIR1 and IAA1/AXR5 or IAA3/SHY2, but not between TIR1 and IAA14/SLR or Aux/IAA7 (Supplementary Fig. S11, A and B). These data indicate that BiAux might enhance the formation of specific auxin co-receptor systems.

**Figure 8.**
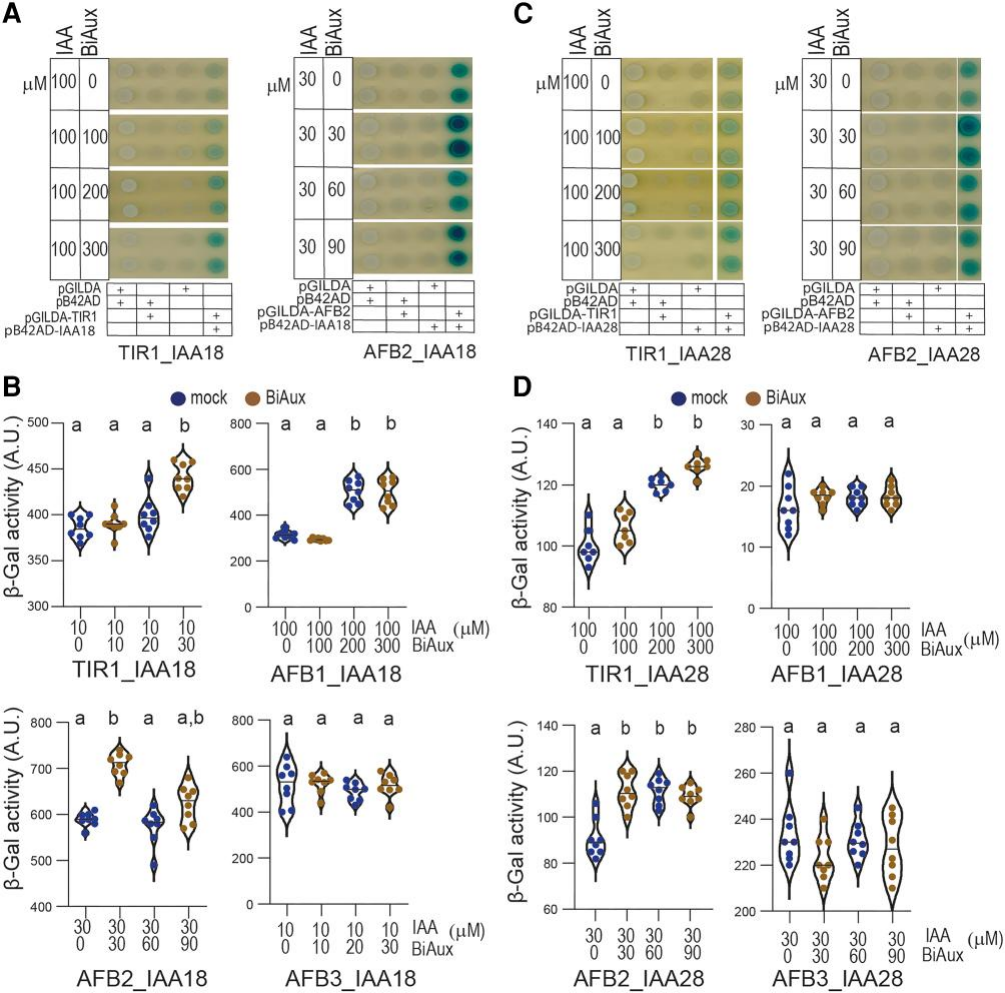
BiAux increases the interaction between TIR1/AFB2 and IAA18 or IAA28. **A)** Y2H interaction experiments between TIR1 and AFB2 with Aux/IAA18 adding different concentration of auxin (IAA) and BiAux as indicated. **B)** Quantification of β-galactosidase in arbitrary units. *n* = 12 (two biological replicates). **C)** Y2H interaction experiments between TIR1 or AFB2 with Aux/IAA28 adding different concentration of auxin and BiAux as indicated. **D)** Quantification of β-galactosidase in arbitrary units. *n* = 12 (two biological replicates). Significance was analyzed by ANOVA and Tukey HSD post-test. *P* < 0.05. Different letters indicate statistical differences.

As BiAux affects TIR1/AFBs interaction with Aux/IAA proteins, we decided to analyze if it regulates IAA18/POTENT and IAA28, two proteins involved in PBS and LR formation and their stability is regulated by auxin (Moss et al. 2015). We found that BiAux enhanced IAA18/POTENT interaction with TIR1, AFB2, and AFB1 in the yeast two hybrid (Y2H) system (Fig. 8, A and B; Supplementary Fig. S12). We also found that the addition of BiAux in combination with IAA significantly increased the interaction between IAA28 and TIR1 or AFB2, but not with AFB1 neither AFB3 (Fig. 8, C and D; Supplementary Fig. S12).

**Figure 9.**
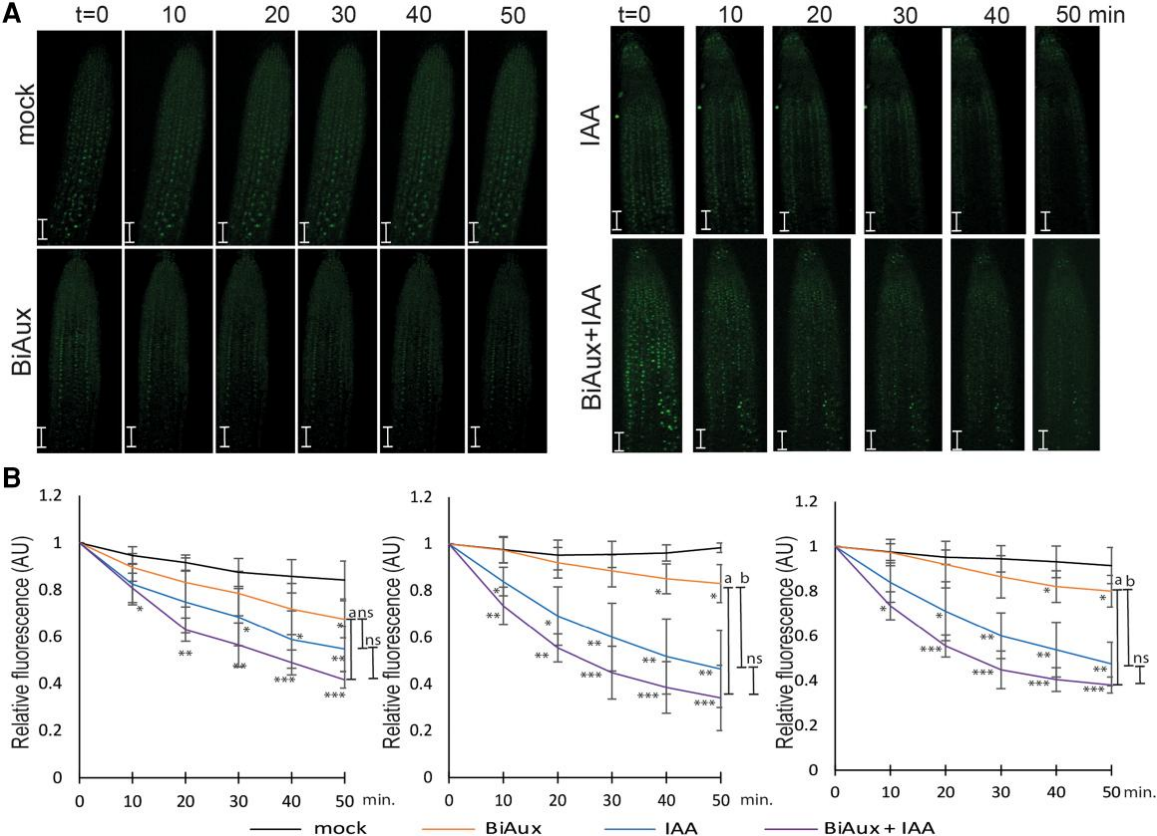
BiAux increases the degradation of IAA28. **A)** IAA28-VENUS signal in root meristem of pHS::IAA28-VENUS seedlings treated with mock (DMSO), 10 *µ*M BiAux, 1 *µ*M of IAA, or 10 *µ*M BiAux plus 1 *µ*M of IAA. Pictures were taken every 10 min. Scale bars correspond to 50 *µ*m. Note that each root is a composite figure. **B)** IAA28-VENUS signal quantification in three different experiments. Asterisks indicate statistical differences between the treatment and mock by *t*-test. *P < 0.05; **P < 0.01; ***P < 0.001. “a” indicates statistical differences between BiAux + IAA and BiAux treatments (*P* < 0.001) in at least three time points. “b” indicates statistical differences between IAA treatment and BiAux (*P* < 0.01) in at least three time points. ns indicates no statistical difference between BiAux + IAA and IAA treatments. Error bars correspond to standard deviation (Sd).

We analyze the role of BiAux in the degradation of IAA28. Seven-day-old pHS::IAA28-VENUS seedlings were incubated with mock, BiAux, IAA or BiAux + IAA and confocal images of the root meristems were taken every 10 min (min). BiAux alone slightly increased IAA28-VENUS degradation, likely due to the presence of endogenous auxin in the root meristem, while IAA addition led to a faster degradation rate (Fig. 9, A and B). Although not significantly different, the degradation rate in BiAux + IAA-treated seedlings tended to be higher than IAA-treated (Fig. 9, A and B), suggesting that both IAA and BiAux act synergistically.

## Discussion

Auxin, an indol-based phytohormone, plays an essential role in the specification and development of LR (Xuan et al. 2015; Du and Scheres 2018). In a nontargeted metabolomic analyses we identified a compound that accumulates in illuminated roots, a condition that induces LR emergence (Silva-Navas et al. 2015). Based on the monoisotopic mass we infer a putative chemical formula that contains two methylated auxins and modified with a *N*-glucoside, we called BiAux. Modification of chemical compounds to improve their activities has been done in many works (Yao et al. 2017). Here, we reported a more effective and sustainable protocol to synthesize a reduced form of BiAux, by using lower-toxic reagents. Remarkably, treatment of Arabidopsis seedlings with BiAux increases the number of PBS and LRP and promotes the emergence of LRs in Arabidopsis plants without affecting root length. This effect indicates a specific function of BiAux in LR formation, one of the multiple auxin-regulated processes.

In nature, most of the IAA is presented in conjugated forms, which are usually classified based on the distinct linked formed via the carboxy group into ester or amide-type conjugates (Casanova-Sáez et al. 2021). In addition, the indole ring of IAA undergoes modifications such as *O*- and *N*-glycosylation (Kai et al. 2007). Many works have reported IAA-conjugates forms, although very few containing an active *N_1_*-β-D-glucopyranosyl-1-*H*-indole-3-acetic acid (IAA-*N*-Glc) modification (Kai et al. 2007; Teichert et al. 2008). However, the identification of such conjugates in plant extracts suggests that *N*-glucosylation of indole can be the common metabolic pathway of IAA in plants (Kai et al. 2007). In the last years, the bisindole natural products, which consist of two monomeric indole units, are gaining interest because of their function as antitumoral molecules (Andreani et al. 2008). Furthermore, there are several examples where bisindoles compounds show higher biological activity than their corresponding monomeric units (Ryan and Drennan 2009; Pandey et al. 2021).

Auxin perception involves the formation of TIR1/AFBs-Aux/IAA co-receptors. Auxin binds to the F-box TIR1/AFBs proteins to stabilize their interaction with Aux/IAA repressors. This stabilization facilitates Aux/IAA ubiquitylation and subsequent degradation (Pierre-Jerome et al. 2013; Salehin et al. 2015). The Arabidopsis genome contains 6 TIR1/AFBs and 29 Aux/IAA proteins, so their combination can generate a large number of co-receptors to control the different auxin responses. Thus to fully understand how auxin regulates multiple processes in planta, we need to identify the mechanisms that control the interaction between the different TIR1/AFBs and Aux/IAA proteins. To explain this control, several possibilities have been proposed, such as common spatial-temporal expression of both co-receptors proteins (or cell-type specific co-expression), intracellular auxin levels (accumulation, conjugation, or transport), or protein modification (Wang and Estelle 2014). To explore the role of auxin levels into this intriguing combinatorial question, an elegant heterologous interaction system based on a Y2H and different auxin concentrations in medium was designed. Using this system, it was shown that the formation of different co-receptors involving TIR1 and Aux/IAA proteins requires a wide range of auxin concentration, suggesting different affinities (Calderon Villalobos et al. 2012). Here, we provide strong evidences that BiAux enhances the interaction between TIR1 or AFB2 with specific members of the Aux/IAA family that are involved in LR formation, conferring, therefore, specificity to auxin signaling during these developmental processes.

In Arabidopsis, regular oscillations of gene expression regulated by the root clock, both in phase or antiphase with the auxin marker DR5::LUC, and fluctuation of the levels of derived auxin from LR cap controls the specification of FC (Moreno-Risueno et al. 2010; Kircher and Schopfer 2018; Xuan et al. 2020). Our data show that BiAux enhances PBS formation by altering the root clock. BiAux treatment increased the amplitude of the DR5::LUC signal without substantially perturbing the frequency, and leading to the formation of more than one PBS/oscillation that were normally in the close vicinity (Fig. 3 A). Interestingly, some PBS loss DR5::LUC expression in the WT, suggesting that they will not develop LRs later. This is in agreement with the idea that several PBS might enter in a dormant stage (Manzano et al. 2012) acting as “PBS reservoirs”. Later, if the environmental conditions (stress, nutritional status, etc.) required more LRs, these dormant PBS could be re-activated to form LRs. The process involves the degradation of Aux/IAA repressors and the activity of *ARF7*, a member of the ARF family involved in PBS formation (Moreno-Risueno et al. 2010) and LR initiation (Okushima et al. 2007). The GATA23–Aux/IAA28 module also controls FC specification (De Rybel et al. 2010). Aux/IAA proteins interact with ARF to repress their transcriptional activity and, to release this repression, Aux/IAA proteins are targeted for degradation in an auxin-dependent manner through the SCF–TIR1/AFB complexes. Structural data demonstrate that auxin binds to TIR1/AFBs to increase the Aux/IAA interaction surface and to stabilize the interaction (Tan et al. 2007). Several gain-of-function mutations affecting *Aux/IAA* genes generated dominant negative mutants that, among other effects, reduced the number of LR. One of these mutant, *iaa28-1*, develops fewer LR that WT seedlings and addition of exogenous IAA or IBA slightly increases the number of LR, indicating that Aux/IAA28 transduces the auxin signaling to form LR (Rogg et al. 2001). In our case, BiAux enhances the TIR1/AFB2 interaction with Aux/IAA28 and enhances its degradation. However, BiAux does not completely overcome the reduction of LR formation in the gain-of-function *iaa28-1* mutant, suggesting that the proper degradation of Aux/IAA28 is indeed required for BiAux function.

*ARF7* accumulates in antiphase during the oscillating gene expression that corresponds with FC specification (Moreno-Risueno et al. 2010). *ARF7* loss-of-function blocks DR5::LUC oscillations (Perianez-Rodriguez et al. 2021), indicating that its function is required for specification of prebranching sites. Interestingly, BiAux regulates a significantly higher number of genes expressed in phase than in antiphase, suggesting that this compound affects the oscillation process. Furthermore, BiAux increases *ARF7* expression in the mature zone, where *ARF7* is required to activate the emergence of the LRP (Okushima et al. 2007). ARF7 activity is negatively controlled by Aux/IAA repressors. One of them, IAA18/POTENT, acts as regulator of the clock oscillations since a dominant mutation that prevent the degradation of IAA18/POTENT protein leads to the formation of massive PBS priming (Perianez-Rodriguez et al. 2021). Interestingly, BiAux treatment of *potent* mutant substantially increases the DR5::LUC expression in the OZ, suggesting a connection between BiAux and IAA18/POTENT signaling. Our analyses do not show active degradation of IAA18/POTENT in roots upon BiAux, although further experiments need to be done to clarify this point.

In the last decade, chemical designed has developed auxin agonist and antagonist to investigate auxin-regulated processes. However, many of these compounds bind in the auxin binding pocket, altering the accessibility of endogenous auxins (Ma et al. 2017). Here, we show that BiAux alone does not allow TIR1–Aux/IAA interaction, but in contrast, it enhances such interaction in presence of auxin. Docking analyses show that BiAux binds to TIR1 in a different site than IAA, becoming an allosteric regulator of auxin signaling. Genetic studies corroborate that BiAux function is dependent on TIR1 and AFB2 activities, but not on AFB1 or AFB3. Furthermore, BiAux increases the interaction between TIR1 or AFB2 with Aux/IAA28 and, to a lesser extent, with IAA18/POTENT. These data, together with the fact that BiAux enhances the interaction of specific Aux/IAA, suggest that BiAux might provide certain specificity to form TIR1/AFBs–Aux/IAA co-receptor systems.

In conclusion, our data support that BiAux acts as positive allosteric regulator of TIR1/AFB2, promoting their interaction with Aux/IAA28 and IAA18/POTENT. Based on our data, we proposed that BiAux promotes FC specification and LR formation by enhancing the auxin-dependent degradation of IAA28 by TIR1 and AFB2. In addition, BiAux might also increase the level of *ARF7* in the differentiation zone to promote the emergence of LR. However, there are several questions that need to be answered: Is BiAux metabolized into the root cells to render a different compound responsible for the PBS and LRs formation? what is the exact glucoside that is attached to the indol? Is really BiAux produced in roots cells? and if yes, what are the enzymes involved? Nevertheless, independently of these questions, our data clearly show that BiAux is able to specifically induce PBS formation, FC activation and LR emergence in Arabidopsis and can be used to better understand these processes in plants. In addition, we show that BiAux increases the number of root meristematic cells that will transit through the OZ. In the other hand, in combination with IAA, BiAux regulates components of the root clock (IAA18 and ARF7). Thus, it is possible that the higher number of cells transiting the OZ, combined with the modulation of the oscillation amplitude, promote the formation of PBS. It would be interesting to include BiAux in the different models that consider cell proliferation, auxin pulses, and root clock (Perianez-Rodriguez et al. 2021; van den Berg et al. 2021).

## Materials and methods

### Plant material

In this work, we used *A. thaliana* (Arabidopsis) accessions plants (ecotype Columbia (Col-0) and Wassilewskija (Ws)). SKP2Bp::GUS expression sites (SKP2BES) in roots (Manzano et al. 2012) was used as a marker for FC and LRP in all stages. The DR5::LUC reporter line was used to monitor the oscillations leading to PBS specification by the root clock in the OZ (Moreno-Risueno et al. 2010). This line was also used to quantify the total number of DR5ES, representing all the DR5::LUC sites along the root and gathers PBS and LRP that express DR5::LUC. The Arabidopsis mutants used were *slr1* (Fukaki et al. 2002), *axr1-12* (Lincoln et al. 1990), *tir1-1*, *afb1-3 afb2-3 afb3-4* (Parry et al. 2009), and their combination generated by cross and selection of the progeny through genotyping by PCR, *iaa28* (Rogg et al. 2001), *arf7* (SALK_040394), and *arf19* (SALK_009879), which were crossed with DR5::LUC and *iaa18/potent* (Perianez-Rodriguez et al. 2021). DR5::LUC reporter or SKP2Bp::GUS were introgressed as indicated in mutant backgrounds by cross and the progeny genotyped by PCR to obtain homozygous stable alleles. To avoid additional stresses generated by light illumination, Arabidopsis seedlings were grown in the D-Root system (Silva-Navas et al. 2015; González-García et al. 2022) to maintain the root system in darkness, unless otherwise indicated.

### Generation of IAA28-VENUS lines and IAA28-VENUS degradation

To generate the pHS::IAA28-VENUS-NLS transgenic lines, we used the plasmid provided by Dra. J. Nemhauser (Moss et al. 2015). We isolated 12 different lines and isolated T3 containing only one insertion. IAA28-VENUS expression was induced by cultivating 7-d-old seedlings at 37 °C in darkness for 2 h. Afterwards, they were mounted on glass slides and covered with a HybriWell sealing system cover (Sigma GBL611202). This sealing cover was filled with 200 *µ*L of half-strength MS (½MS) containing mock (DMSO), 10 *µ*M BiAux, 1 *µ*M of IAA, or 10 *µ*M BiAux plus 1 *µ*M of IAA. Stack confocal images were taken every 5 min in an automatized mode using the Leica SP8 microscope in a quantum mode (scale 15/255). The Argon laser was utilized at an intensity of 19.84%, and 488 nm was employed for excitation, with the intensity set to 63%. The collection bandwidth for the HYD channel was adjusted to range between 500 and 545 nm. Additionally, no gain or offset values were selected; both remained at zero. Quantification of the IAA28-VENUS level in the root meristems were done using the LasX software using the maximum protection of all stacks. All values were relativized to the value at time 0 for each treatment. We carried out three independent experiment and analyzed three root meristems in each experiment.

### UHPLC/ESI–QTOF–MS

The root samples as well as the synthesized BiAux were analyzed using ultra-high performance liquid chromatography with electrospray ionization, couple to quadrupole-time-of-flight-mass spectrometry (UHPLC/ESI–QTOF–MS). Briefly, 500 *μ*L of methanol was added to grounded root samples. The mixture was vortexed for 2 min, sonicated for 5 min, and centrifuged at 10,000 *× g* for 10 min at 4 °C. The supernatant was liophylizated and then resuspended in 300 *μ*L of methanol, vortexed for 2 min, sonicated for 5 min, and centrifuged at 10,000 *× g* for 10 min at 4 °C. The supernatant was transferred to a Chromacol vial (Thermo Fisher Scientific, Madrid, Spain) for LC–MS analysis. The procedure was performed in duplicate. The samples were analyzed on a 1,290 Infinity series UHPLC system equipped with an electrospray ionization source (ESI) with Jet Stream technology coupled to a 6,545 iFunnel QTOF-MS system (Agilent Technologies, Waldbronn, Germany). The separation was performed in the reverse phase column Zorbax Eclipse XDB-C18 4.6 × 50 mm, 1.8 *µ*m (Agilent Technologies), being maintained at 40 °C. The flow rate was 0.5 mL/min with a mobile phase consisting of solvent A: 0.1% (v/v) formic acid, and solvent B: methanol. The elution was carried out in gradient mode and included 2% B (0 to 6 min), 2% to 50% B (6 to 10 min), 50% to 95% B (11 to 18 min), 95% B for 2 min (18 to 20 min), and returned to starting conditions 2% B in 1 min (20 to 21 min) to finally keep the re-equilibration with a total analysis time of 25 min. The flow rate was 0.5 mL/min, and the injection volume was 2 *μ*L. Detector was operated in full scan mode (*m/z* 50 to 1,500), at a scan rate of 1 scan/s both in positive and negative ESI modes. Accurate mass measurement was assured through an automated calibrator delivery system that continuously introduced a reference solution, containing masses of *m/z* 121.0509 (protonated purine) and *m/z* 922.0098 (protonated HP-921) in positive ESI mode; whereas *m/z* 119.0363 (proton abstracted purine) and *m/z* 966.0007 (formate adduct of HP-921) in negative ESI mode. The capillary voltage was ±4,000 V for negative and positive ionization modes. The source temperature was 225 °C. The nebulizer and gas flow rate were 35 psig and 11 L/min, respectively, fragmentor voltage to 175 V, and a radiofrequency voltage in the octopole (OCT RF Vpp) of 750 V. The Mass Hunter Workstation Software LC/MS Data Acquisition Version B.07.00 (Agilent Technologies) was used for control and data acquisition. LC–QTOF–MS data processing was performed in MassHunter Qualitative Analysis (Agilent Technologies) Software version B.08.00.

### BiAux characterization

From previous studies, we focused mainly on an unknown compound detected in a LC–MS analysis and found to significantly increase its level in illuminated roots (Silva-Navas et al. 2019). The only data extracted from the analysis of this compound was the protonated ion peak at *m/z* 707.2451 as deprotonated ion. A search was done using ChemCal software (https://www.chemcalc.org/) using the following parameters: mass: 707.2451; accuracy: 10; range: C0 to 50 H0 to 50 N0 to 10 O0 to 20; ionization: H^−^; unsaturation filter: 0 to 20. Afterwards, ChemSpider database (http://www.chemspider.com/Search.aspx) was used to obtain candidate structures for a target molecular formula.

### BiAux synthesis

BiAux compound was synthesized as described in Supplementary Fig. S1A and Materials and methods.

### Root morphological analyses

Seedlings were grown in ½MS medium during the indicated days containing the amount of IAA, BiAux as indicated. Seedlings were cultivated with the roots in presence of light or in darkness using the D-Root device (Silva-Navas et al. 2015). Afterwards, they were scanned to high resolution with an Epson 600V scanner and root length were quantified using Fiji software. To quantified LRP, wild type of mutant crossed with SKP2Bp::GUS seedlings were stained for GUS activity as described by Silva-Navas et al. (2019) and LRP-GUS stained were quantified in a stereomicroscope Leica Z9.

### Luciferase imaging and expression analysis

DR5::LUC line in wild type or mutant backgrounds were cultivated in ½MS medium containing different concentration of auxin or BiAux. After the specified days of incubation, plates containing seedlings were sprayed with 1 mL of 2.5 mM potassium luciferine (Gold Biotechnology, St. Louis, Mo., Goldbio.com, cat. no: LUCK-1). Luminescence was acquired using a NightOwl II (Berthold), or a Flumazone (Leica M205FA adapted with Hamamatsu EMCCD × 2 camera). Expression was measured by selecting the region of interest and quantifying the analog–digital units per pixel using the MetaMorph Image Analysis Software. The intensity profile along the roots was measured with MetaMorph Image Analysis Software. The luciferase measurements were referred to as the percent change with respect to its own control. The number of DR5ES with high expression relative to the adjacent regions along the primary root was used to approximately reflect PBS spacing. The values were represented as relative (percentage) to the number of DR5ES in the control, by dividing each value by the mean of the control plant treated with mock.

For time lapse experiment, DR5::LUC seedlings were grown in ½MS for 4 d and then they were transferred to fresh medium containing either mock (DMSO) or 5 *µ*M of BiAux for two more days. Afterwards, roots we transfer to fresh plates containing mock or BiAux and a time lapse record was made for vertical root growth during 24 h. A video clip was composed using images that were taken every 20 min with the flumazone camera and recorded with the MetaMorph software. To create the kymograph a line was drawn from the upper part of the OZ at time 0 until the end of the root at time 24 h (this line reflects the growth over 24 h). Subsequently, the Kymograph tool was selected and a line width of 5 was used for the previously generated line.

To profile expression of ARF7, the maxima intensity along the root of pARF7::LUC treated with mock or BiAux was analyzed with the Linescan tool. A line was draw along the root (middle section) in the growth zone from the meristem to about 3 cm shootward.

### Docking and molecular structures

The 3D geometry of BiAux was modeled and optimized with Chimera 1.13. For the TIR1–ASK1 complex, we used its crystal structure in the PDB id. 2P1M in our analyses. Since AFB1, AFB2, or AFB3 proteins have no experimental structures available, we modeled them from their amino acid sequences using the “User Template Mode” of the Swiss-Model service and giving the crystal structure of TIR1 in 2P1M as template. The 3D models of AFB1 and AFB3 had coverages of 98% and 97%, respectively, and Global Quality Model Estimator (GMQE) scores of 0.83 and 0.81, respectively. This score is defined in a (0.0 to 1.0) scale with higher values indicating 3D models of higher quality.

The geometries of BiAux bound to TIR1, AFB1, and AFB3 were obtained by means of protein–ligand docking calculations performed with AutoDock Vina, selecting the best solutions (i.e. those having the lowest protein–ligand affinity Δ*G* computed by Vina). Since docking methods include ligand flexibility but keep the protein fixed, we explored the conformational flexibility of the TIR1–ASK1–BiAux complex by optimizing its complete structure. To this end, we immersed the docking complex in water setting a periodic solvation box with 15 Å spacing in all dimensions and Na^+^ and Cl^−^ ions added to counter total charge and set 0.150 M salt concentration. The optimization was achieved through 5,000 minimization steps with the conjugate-gradient algorithm implemented in NAMD. As shown in Fig. 6, the optimized geometry of TIR1 and ASK1 proteins in the complex with BiAux, including the conformations of residue side chains in the binding site, are in very close agreement with the geometries of both backbone and side chains of TIR1 and ASK1 in the complex with IP6 in the crystal structure 2P1M. Therefore, we did not consider necessary to optimize the structures of the BiAux complexes with AFB1 and AFB3 proteins. All the molecular graphics were prepared and rendered with PyMOL 2.3.2.

### Electrostatic potentials

Poisson–Boltzmann electrostatic potentials (PB-EPs) were obtained by solving numerically the nonlinear PB equation with the APBS 1.5 program using the input interface implemented as a plug-in in PyMOL 2.3.2. These APBS calculations were performed in sequential-focusing multigrid mode with dielectric constants 4 for proteins and 78.54 for water at 0.150 M NaCl concentration. 3D grids defined by 193^3^ = 7,189,057 points for proteins and protein–ligand complexes were employed in the multigrid APBS calculations. PB-EPs were mapped onto the molecular surfaces and are given in *kT*/*e* units, *k* being the Boltzmann´s constant, *T* is the absolute temperature = 298 K, and *e*, the unit electron´s charge.

### Transcriptomic analyses

Wild type and *tir1-1* Arabidopsis seedling were grown in ½MS for 4 d and then they were transferred to fresh medium containing mock, 5 *µ*M of BiAux, 10 nM of IAA or 5 *µ*M of BiAux plus 10 nM of IAA for three more days. Afterwards, roots were harvested, and RNA extracted using the RNeasy kit (Qiagen) following manufacturer's instructions. RNAseq analyses (100 PE) were carried out by BGI company obtaining 30 M of reads/library (quality 96% as average). Library construction and RNA sequencing were performed by Beijing Genomics Institute (BGI-Shenzhen, Shenzhen, China). About 20 *µ*g of total RNA was subject to poly(A^+^) RNA isolation by oligo-dT chromatography, followed by RNA fragmentation. Fragmented RNAs were converted into double-stranded cDNA using random-hexamer primers followed by end repair, 3′ end adenylation and adapter ligation. cDNA fragments were selected by agarose gel extraction and enriched by PCR amplification. The library was loaded onto an Illumina HiSeq 2000 instrument for pair-end sequencing. The average read length of 100 bp was generated as raw data.

Prior to assembly, FastQC (Andrews 2010) (v0.11.9) was used to obtain information about the quality of the sequencing data. This information was used for the initial filtering of sequences by Trimmomatic (v0.36) (Bolger et al. 2014). For each sample, RNA-seq raw reads (pair-end, 100 bp) were trimmed to remove the potential Illumina adaptor contamination and conduct read trimming and clipping of the low-quality bases. The remaining reads were aligned to the *A. thaliana* (TAIR10 genome reference) using the Araport11 annotation (Cheng et al. 2017) by STAR aligner (v2.5.3a) (Dobin et al. 2013). Based on the RNA-seq mapped reads and the Araport11 annotation, HTSeq (v1.99.2) (Anders and Huber 2010) with the intersection “union” option was employed to generate the read counts per gene. Normalization and statistical analyses of differential gene expression was conducted with EdgR Bioconductor package in R (Anders and Huber 2010; Love et al. 2014). A multiple-test corrected *P*-value (Benjamini and Hochberg 1995) of 0.05 was employed. Differential expression analysis was calculated with EdgR and differentially expressed genes were considered for those genes that showed a *P-*value < 0.05 and a fold-change log_2_ > 0. 5 or <−0.5.

Venn diagrams were generated with the interactive Venn tool (http://www.interactivenn.net/). GO categorization was generated with metascape tool (https://metascape.org/) (Zhou et al. 2019), using the following custom enrichment parameters: (i) minimum overlap = 3; (ii) *P*-value cutoff = 0.05; (iii) minimum enrichment = 1.5.

### Y2H assays

TIR1, AFB1, AFB2, and AFB3 coding regions were cloned in the into the Y2H bait vector pGILDA vector. IAA1, IAA3, IAA7, IAA14, IAA18 (POTENT), and IAA28 were cloned in the pB42AD (Clontech) (Calderon Villalobos et al. 2012). Transformation and interaction were analyzed as described by Prigge et al. (2010). Homogenous colonies were spotted on SD-galactose/raffinose inducing medium containing—Ura/–His/–Trp drop out supplement, 80 *µ*g/mL X-Gal, and IAA or BiAux in the indicated concentration. Plates were incubated for 3 d at 30 °C and β-galactosidase staining reported the IAA-dependent protein–protein interaction. Plates were scanned in RGB-color and blue intensity of the colonies was quantified using the measurement tool within Fiji. First, we quantified the blue in mock and BiAux colonies, which were no-statistically different. Then, the average of blue from these colonies was subtracted from the values of those spots in the IAA or IAA + BiAux treatments.

### GO and statistical analyses

GO was done using the Metascape tool (https://metascape.org/gp/index.html#/main/step1). The analyses were done using the following parameters: minimum overlapping of 3, *P*-value cutoff of 0.05, and a minimum enrichment of 1.5. Statistical calculations were performed using PRISM8.1 (GraphPad, San Diego, CA, USA). Comparisons between two groups were performed with Student *t*-test, while multigroup comparisons were performed using one-way analysis of variance (ANOVA), followed by Turkey's test. The *P*-values of <0.05 were considered statistically significant and indicated by different letters.

To compare common expression of genes (Venn diagram) we used the interacting Venn web (http://www.interactivenn.net/) and significance of gene overlapping was analyzed by a Binomial test (*α* = 0.005 and *P*-value < 0.05).

### Accession numbers

RNA-seq data are deposited in the GEO Data Bank (GSE234606). Sequence data from this article can be found in the GenBank/EMBL data libraries under accession numbers: TIR1, 825473; AFB1, 828045; AFB2, 822296; AFB3, 837838; ARF7, 100191131; ARF19, 838505; IAA18/POTENT, 841623; IAA28, 832658; IAA3/SHY2, 839570; IAA1/AXR5, 827103; IAA7/AXR2, 821879, IAA14/SLR, 827102.

## Supplementary Material

kiae090_Supplementary_Data

## Data Availability

The data that support the findings of this study are available on request from the corresponding author, J.C.P. RNA-seq data are deposited in the GEO Data Bank (GSE234606).

## Supplementary data

The following materials are available in the online version of this article.

**Supplementary Figure S1.** LC–ESI analysis of synthesized BiAux.

**Supplementary Figure S2.** BiAux, but not the intermediate used for its synthesis, increases LR formation.

**Supplementary Figure S3.** BiAux and IAA do not produce similar effects.

**Supplementary Figure S4.** BiAux application does not reduce root meristem size but affects meristematic cell number and size.

**Supplementary Figure S5.** BiAux enhances the expression of IAA-regulated genes.

**Supplementary Figure S6.** AFB1 and AFB3 do not contribute to BiAux signaling.

**Supplementary Figure S7.** Docking analyses of BiAux binding in tomato TIR1 and TIR1-like proteins.

**Supplementary Figure S8.** BiAux signaling is mediated by ARF7.

**Supplementary Figure S9.** BiAux regulated genes that are preferentially expressed in phase with the DR5::LUC expression in the OZ.

**Supplementary Figure S10.** BiAux activity in *iaa28-1* mutant.

**Supplementary Figure S11.** BiAux increases the interaction between TIR1 and specific Aux/IAA proteins.

**Supplementary Figure S12.** Y2H interaction experiments between TIR1, AFB1, AFB2, or AFB3 with Aux/IAA18 or Aux/IAA28.

**Supplementary Table S1.** Identification of the molecular formula corresponding to a monoisotopic mass.

**Supplementary Table S2.** BiAux and IAA deregulated common and different set of genes.

**Supplementary Table S3.** BiAux gene induction is mediated by *ARF7*.

## Dive Curated Terms

The following phenotypic, genotypic, and functional terms are of significance to the work described in this paper:

AFB2 Gramene: AT3G26810
AFB2 Araport: AT3G26810

IAA28 Gramene: AT5G25890
IAA28 Araport: AT5G25890

AFB3 Gramene: AT1G12820
AFB3 Araport: AT1G12820

ABP1 Gramene: AT4G02980
ABP1 Araport: AT4G02980

IAA14 Gramene: AT4G14550
IAA14 Araport: AT4G14550

IAA7 Gramene: AT3G23050
IAA7 Araport: AT3G23050

TIR Gramene: AT1G72930
TIR Araport: AT1G72930

TMK1 Gramene: AT1G66150
TMK1 Araport: AT1G66150

ARF19 Gramene: AT1G19220
ARF19 Araport: AT1G19220

LBD29 Gramene: AT3G58190
LBD29 Araport: AT3G58190

TIR1 Gramene: AT3G62980
TIR1 Araport: AT3G62980

IAA1 Gramene: Os01g0178500
IAA1 Araport: Os01g0178500

root AmiGo: PO:0009005

axr1-12 Gramene: AT1G05180
axr1-12 Araport: AT1G05180
