# Supplementary material for: Synthetically derived BiAux modulates auxin co-receptor activity to stimulate lateral root formation
Source: Plant Physiol. 2024 Feb 20;195(2):1694–711. doi: 10.1093/plphys/kiae090 (PMC11142373; doi:10.1093/plphys/kiae090)
Supplement: kiae090_Supplementary_Data [file kiae090_supplementary_data.zip › Supplemental material_v2.pdf]

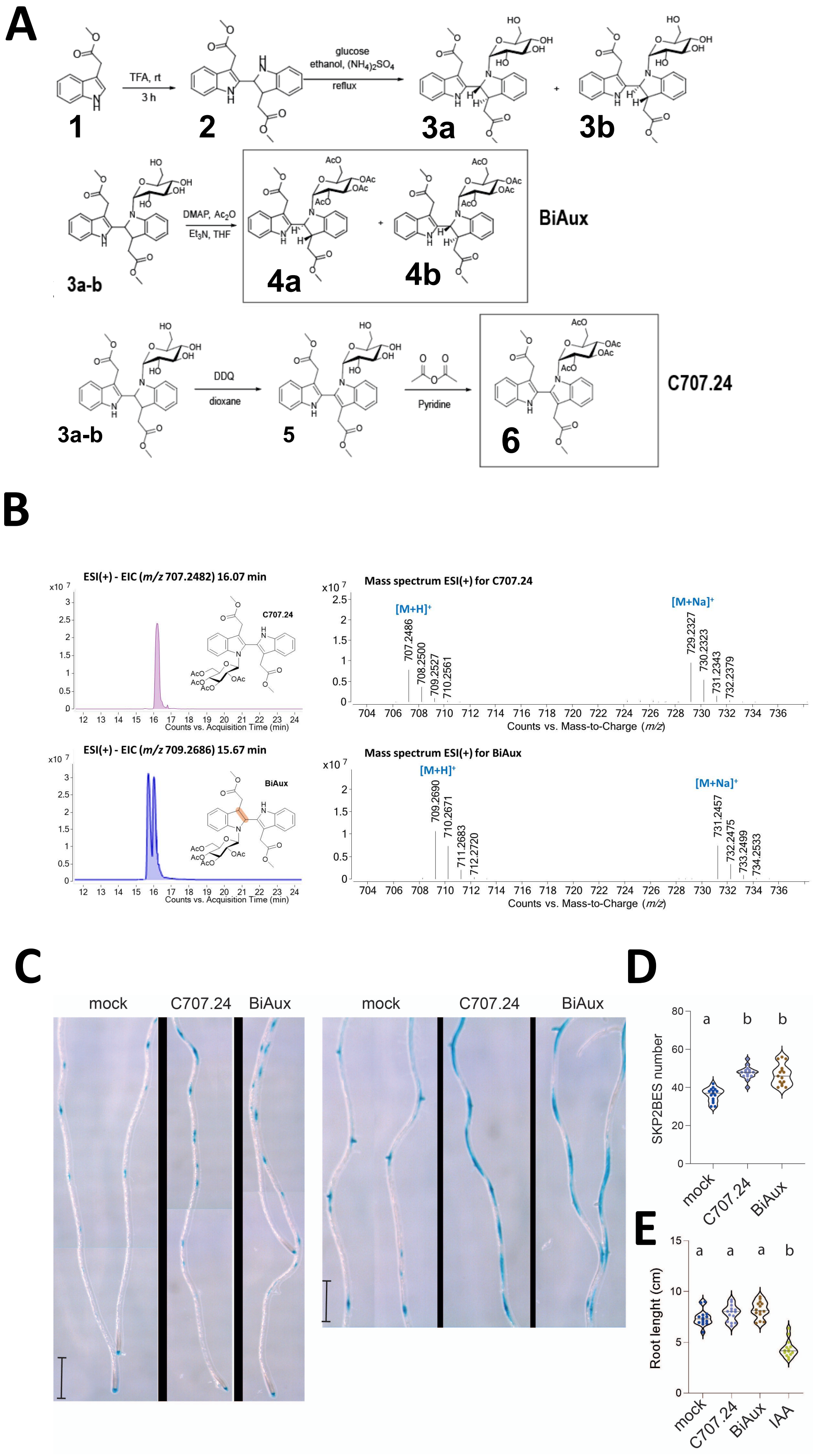

Supplemental Fig S1. LC-ESI-MS analysis of synthesized BiAux. A) Schematic procedure for the synthesis process of BiAux and C707.24 compound (for details see supplemental methods). **B)** Extracted ion chromatograms (EIC), in positive ionization mode, obtained from LC-ESI-QTOF-MS analysis and their corresponding mass spectra for the chemically synthesized BiAux or C707.24. EIC for BiAux of *m/z* 709.2686 yielded two isomeric peaks at 15.67 and 16 min that correspond to the diasteroisomers BiAux synthesized. Orange ellipse labels the reduced form in position C2 and C3. EIC for C707.24 of *m/z* 707.2488. **C)** GUS stained roots of SKP2Bp::GUS seedlings grown for 8 days in the medium containing mock (DMSO) or 5 µM of BiAux or 5 µM of C707.24. Representative pictures of the root tip (left) or a middle region of the roots were taken. Scale bars correspond to 0.5 cm. Note that some of the root images shown are composite figures made of multiple images. **D)** Quantification of the number of lateral root primordia (LRP), counted as positive GUS-stained spots. **E)** Root length of SKP2Bp::GUS seedlings germinated for 4 days in 1/2MS medium and then transferred to fresh medium containing DMSO (mock), 5 µM of BiAux, 5 µM of C707.24 or 0.5 µM of IAA for 4 extra days. Significance was analyzed by ANOVA and Tukey

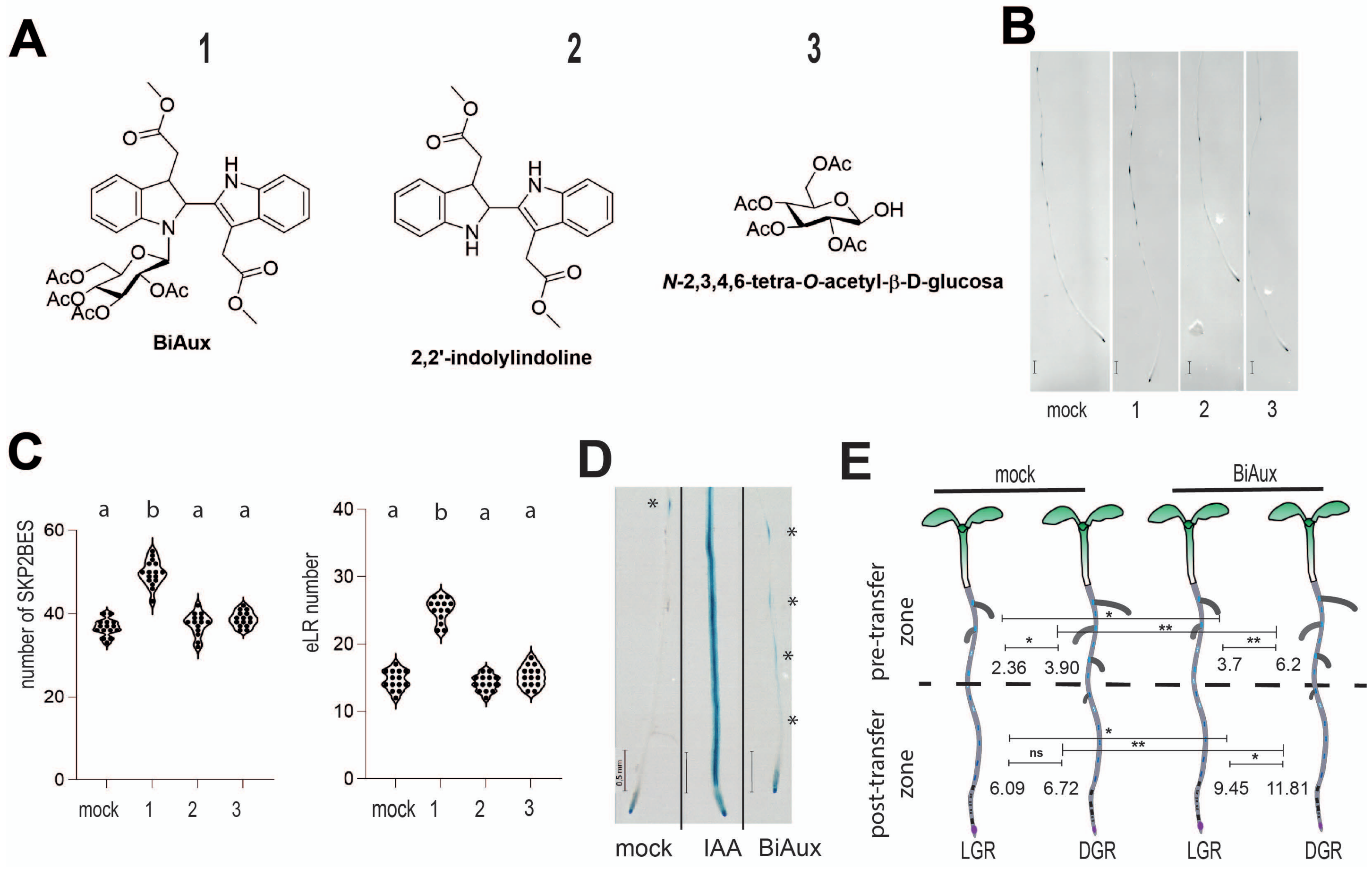

Supplemental Fig. S2. BiAux, but not the intermediate used for its synthesis, increases LR formation. A) Chemical structures of intermediate molecules used for BiAux syntheses. 1: BiAux; 2: 2,2´-indolylindoline; 3: *N*-2,3,4,6-tetra-*O*-acetyl-β-D-flucose. **B)** Representative pictures of GUS stained roots of SKP2Bp::GUS seedlings grown for 8 days in a 1/2MS medium supplemented with DMSO (mock), 5 µM of BiAux, 5 µM of 2,2´-indolylindoline or 5 µM of *N*-2,3,4,6-tetra-*O*-acetyl-β-D-glucose. Scale bar correspond to 0.5 cm. **C)** Number of SKP2Bp::GUS expression sites (SKP2BES) or emerged lateral root (eLR) in Arabidopsis seedlings grown as in (B). n≥12 (1 biological replicate). **D)** Representative pictures of SKP2Bp::GUS roots grown in 1/2MS for 4 days and then transferred to fresh medium containing DMSO (mock), 0.5 µM of IAA, or 5 µM of BiAux for 1 day and then stained for GUS activity. Scale bar correspond to 0.5 cm. **E)** Number of DR5::LUC expression sites (DR5ES) in Arabidopsis seedlings grown in light-grown root (LGR) or dark-grown root (DGR) (Silva et al. 2015) for 4 days and then transferred to fresh medium containing DMSO (mock) or 5 µM for BiAux for 3 days. Asterisks indicate significant differences by t-test. *, $p<0.05$; ** $p<0.01$. Dash line indicates the transference point. Pre-transfer zone: root portion from shoot-root junction until the line (grown in 1/2MS medium). Post-transfer zone: root portion from line to root tip, that has been grown in 1/2MS medium containing mock, IAA or BiAux. Blue spots in the root cartoons represent DR5::LUC expression sites and the dark-pink the oscillation zone.

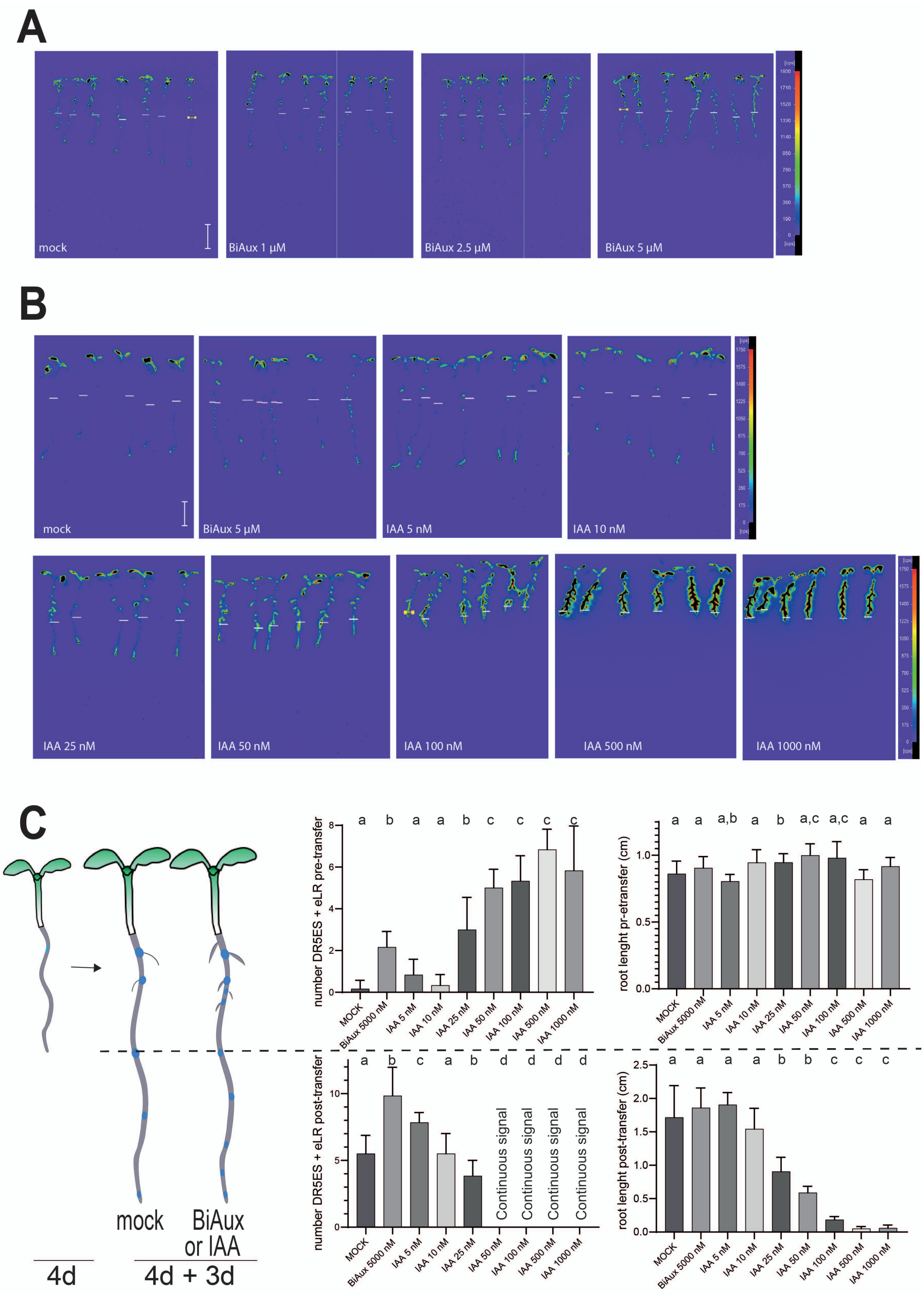

Supplemental Fig. S3. BiAux and IAA do not produce similar effects. A) Representative Luminescence pictures of DR5::LUC seedlings germinated for 4 days in MS1/2 medium and then transferred to fresh medium containing DMSO (mock) or 1, 2.5 or 5 µM of BiAux for 4 extra days. Scale bar correspond to 1 cm. **B)** Representative Luminescence pictures of DR5::LUC seedlings germinated for 4 days in 1/2MS medium and then transferred to fresh medium containing DMSO (mock), or 5 µM of BiAux or 5, 10, 25, 50 100, 500 or 1000 nM of IAA. Scale bar correspond to 1 cm. **C)** Root growth and number of DR5ES and eLRs of DR5::LUC seedlings grown as in (b) and quantified in the pretransfer and post-transfer growth root zones. n≥12 (1 biological replicate). Pre-transfer zone: root portion from shoot-root junction until the line (grown in 1/2MS medium). Post-transfer zone: root portion from line to root tip, that has been grown in 1/2MS medium containing mock, IAA or BiAux. Blue spots in the root cartoons represent DR5::LUC expression sites. Error bars correspond to standard deviation (SD).

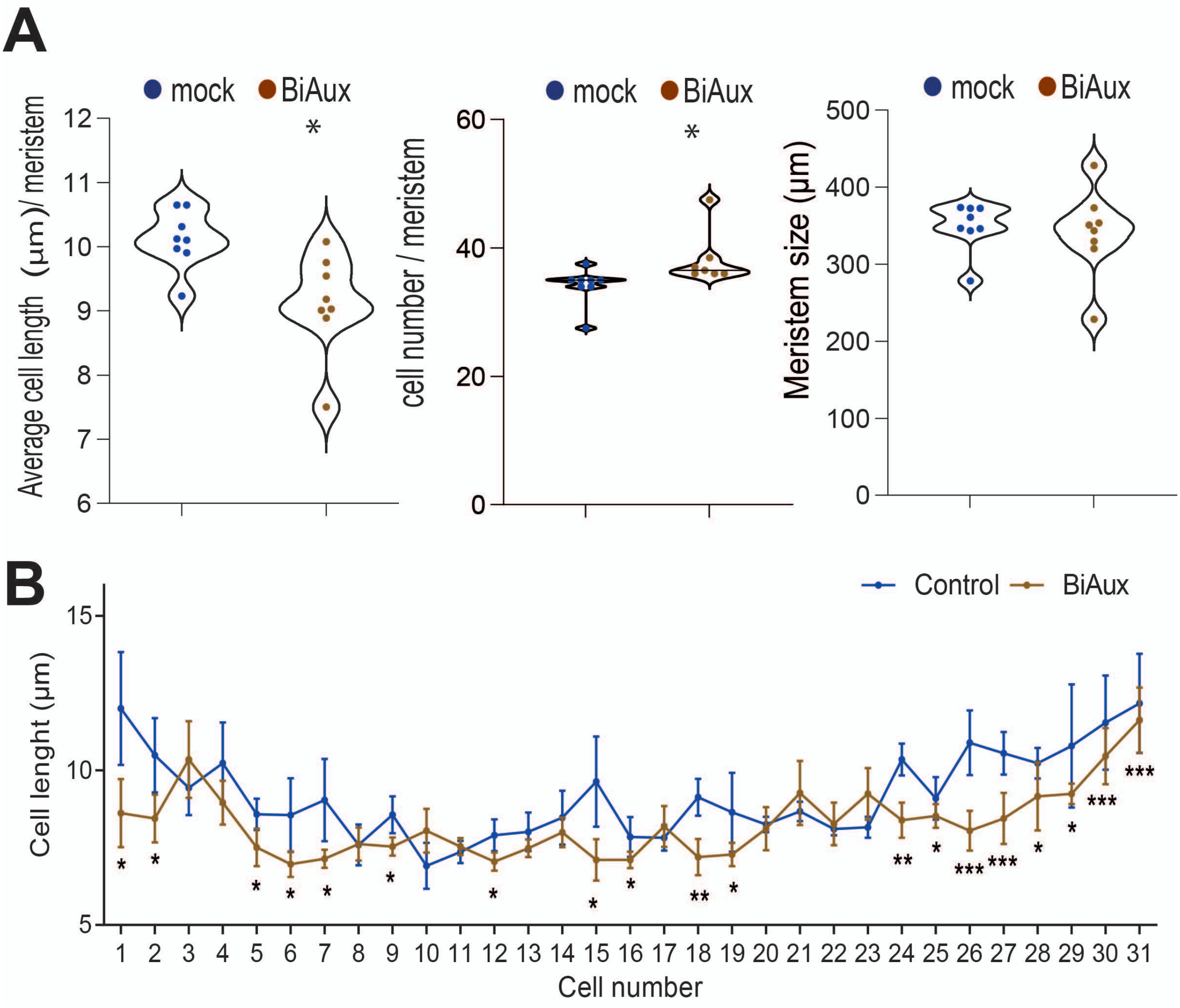

Supplemental Fig. S4: BiAux application does not reduce root meristem size but affects meristematic cell number and size. A) Root meristem of Arabidopsis seedlings grown on medium containing mock or 5 μM of BiAux were stained with propidium iodide and analyzed by confocal microscopy. From left to right, average meristematic cell length (μm), average number of meristematic cortical cell number and meristem size (μm) were quantified (n=10). Asterisks indicate significant differences by t-test. *, $p<0.05$. **B)** Cell length of the first 31 cortical meristematic cells were quantified and represented according to their position in the meristem. n=15 roots. Asterisks indicate significant differences by t-test. *, $p<0.05$; **, $p<0.01$; ***, $p<0.001$. Error bars correspond to stardard deviation.

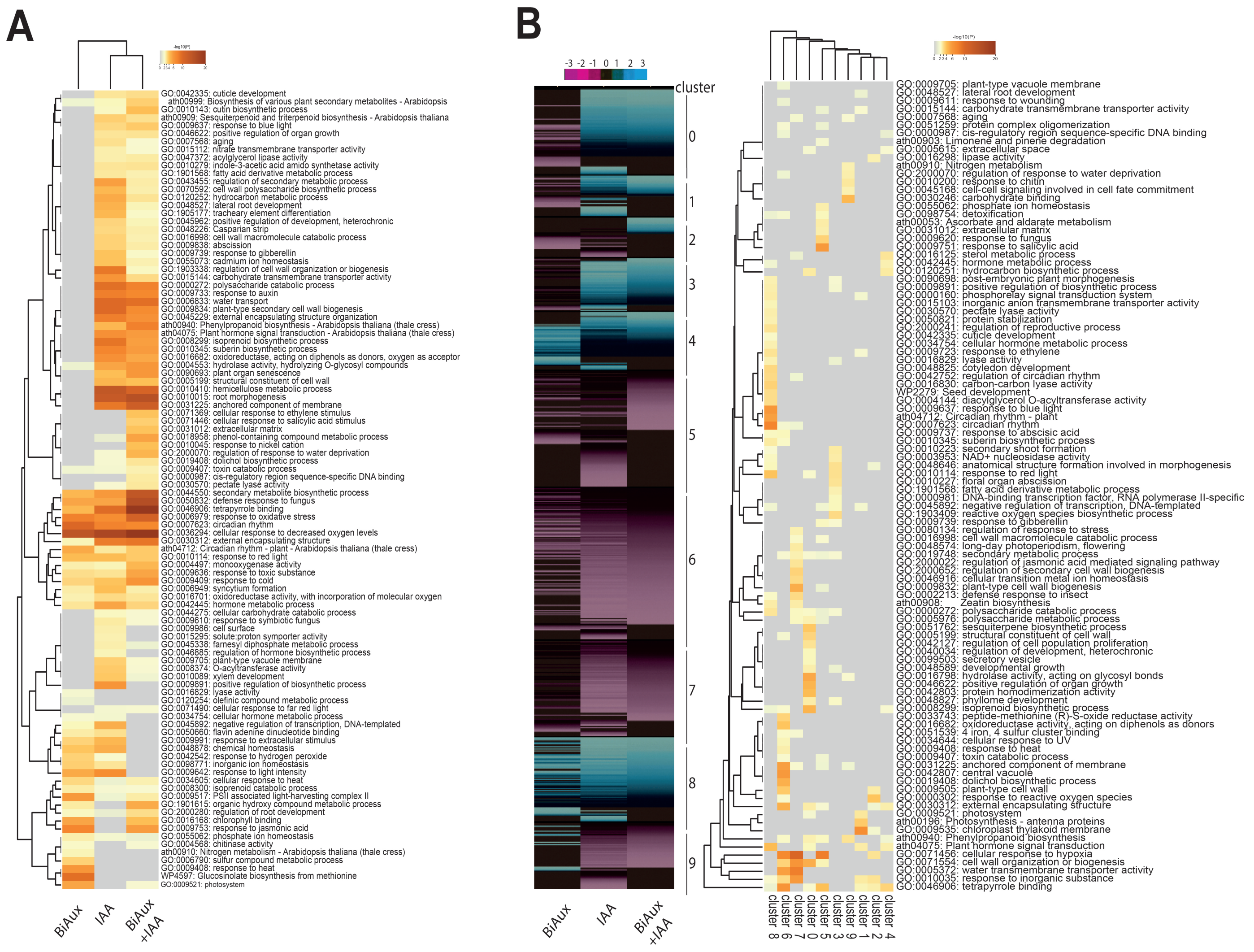

Supplemental Fig. S5: BiAux enhances the expression of IAA-regulated genes. A) Gene ontology classification of genes de-regulated by IAA, BiAux or IAA+BiAux treatments. **B)** Cluster of genes deregulated by BiAux, IAA or BiAux+IAA versus mock in roots. Right panel shows the gene ontology of the different cluster identified. Scale bar corresponds to the expression level respect to mock.

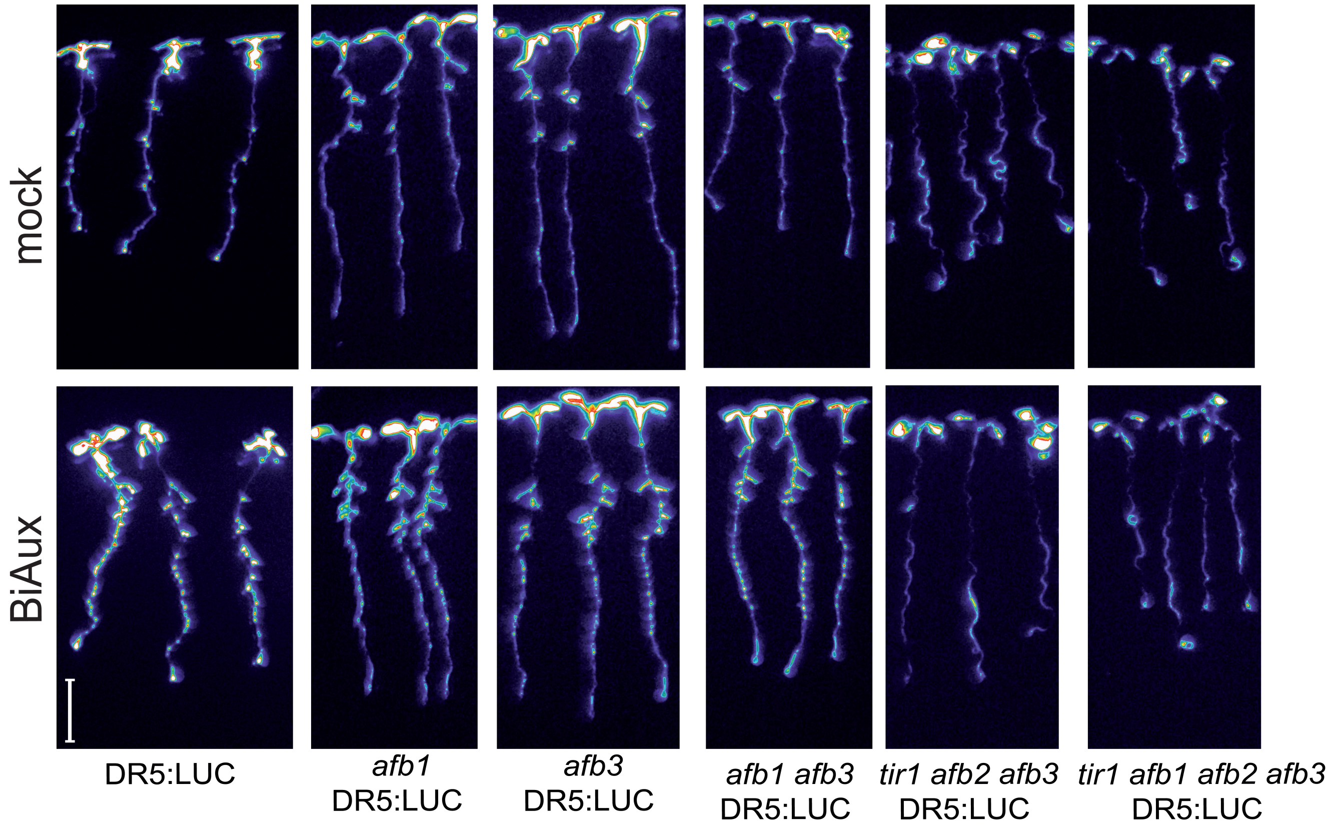

Supplemental Fig. S6: AFB1 and AFB3 do not contribute to BiAux signaling. Representative pictures of luciferase signal in control seedlings, *afb1*, *afb3*, *tir1 afb3, tir1 afb2 afb3 or tir1 afb2 afb1 afb3* mutants germinated for 4 days in 1/2MS and then transferred to a fresh medium containing mock or 5 µM of BiAux during 4 extra days. Notice that only double, triple or quadrupole mutants containing the combination *tir1 afb2* did not respond to BiAux. Scale bar corresponds to 1 cm.

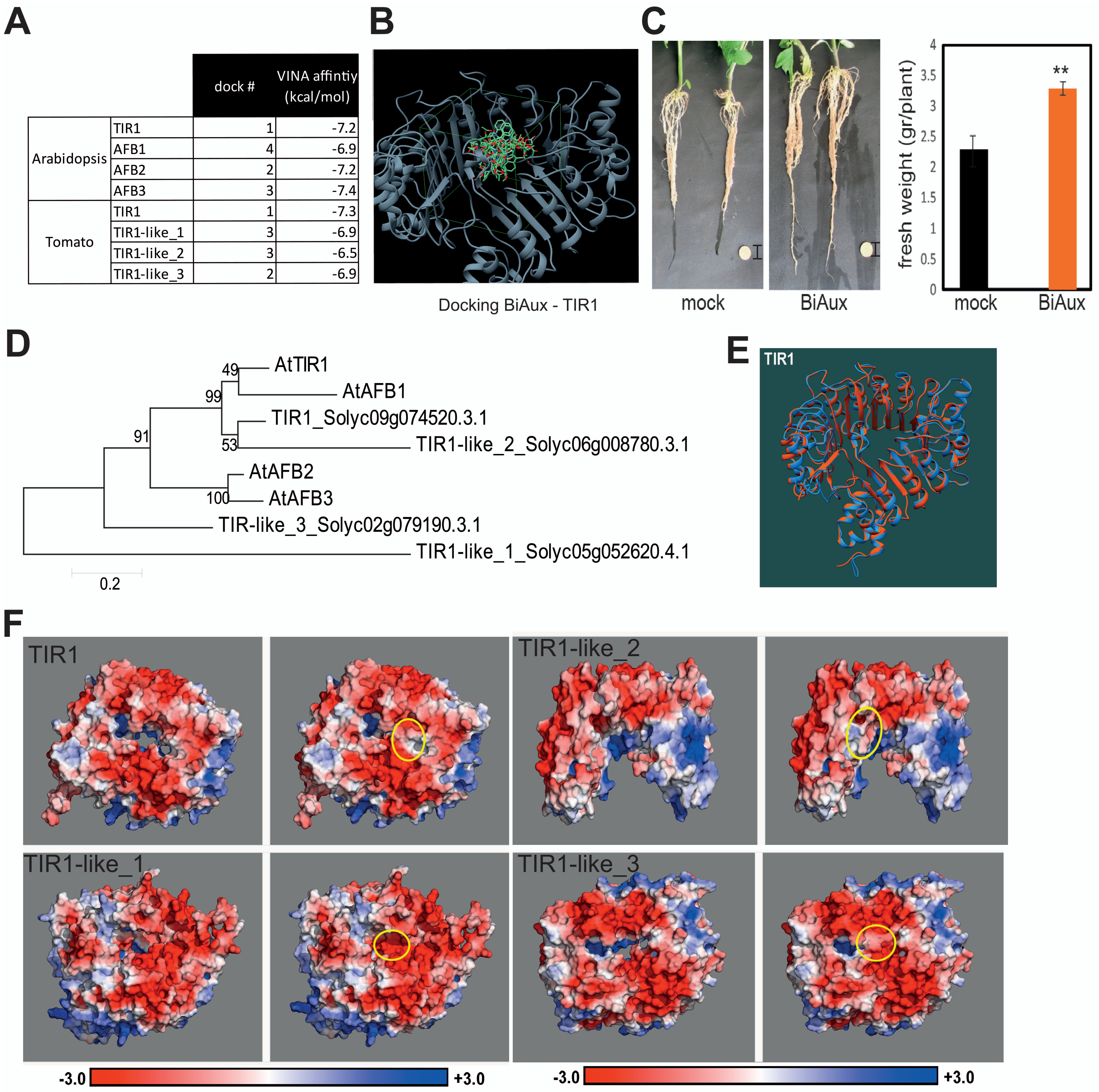

Supplemental Fig. S7: Docking analyses of BiAux binding in tomato TIR1 and TIR1-like proteins. **A**) Number of docking number of analyses and free energy (kcal/mol) estimation between BiAux and Arabidopsis TIR1 and AFBs or tomato TIR1 and TIR1-like proteins. **B)** Multiple docking representation of BiAux in TIR1. Superposition of the crystal structure of the TIR1 (dark blue ribbon) and BiAux (sticks with carbons in green/red) complex.

C) Representative phenotype of roots from tomato plants that were grown in vermiculite for 4 weeks and irrigated with 1/8 of Murashige and Skoog (MS) medium salts every 3 days and 1 µM of BiAux or mock once a week. Graph shows the fresh weight (gr per plant) of tomato roots grown as indicated. n= 10 (1 biological replicate). Asterisks indicate the statistical significance by a t-test **, pval<0.01. Scale bar corresponds to 2.5 cm. Error bars correspond to standard deviation (SD). **D)** Phylogenetic tree of Arabidopsis TIR1 and AFB1-3 and tomato TIR1 and TIR1-like proteins. **E)** Structural superposition of Arabidopsis (blue cartoons) and in tomato (red orange cartoons) TIR1 proteins. Template modeling (TM)-score and root-mean-square deviation (RMSD) between them are 0.993 and 0.407 A (569/581 residues), demonstrating that both proteins in Arabidopsis and tomato are extremely similar. **F)** Poisson-Boltzmann electrostatic potential (PB-EP) mapped onto the molecular surface of tomato TIR1 and TIR1-like proteins viewed at the side of the BiAux-binding site. Left images show the proteins in the absence of BiAux and right images correspond to protein-BiAux complexes, with the surface of BiAux marked with yellow ellipses. The bottom scale bar indicates the range of PB-EP values (in kT/e units) used in these images. Note that in tomato TIR1 and TIR1-like_2, BiAux generates mainly neutral potential, while in TIR1-like_1 and TIR1-like_3 BiAux generates a strong negative potential.

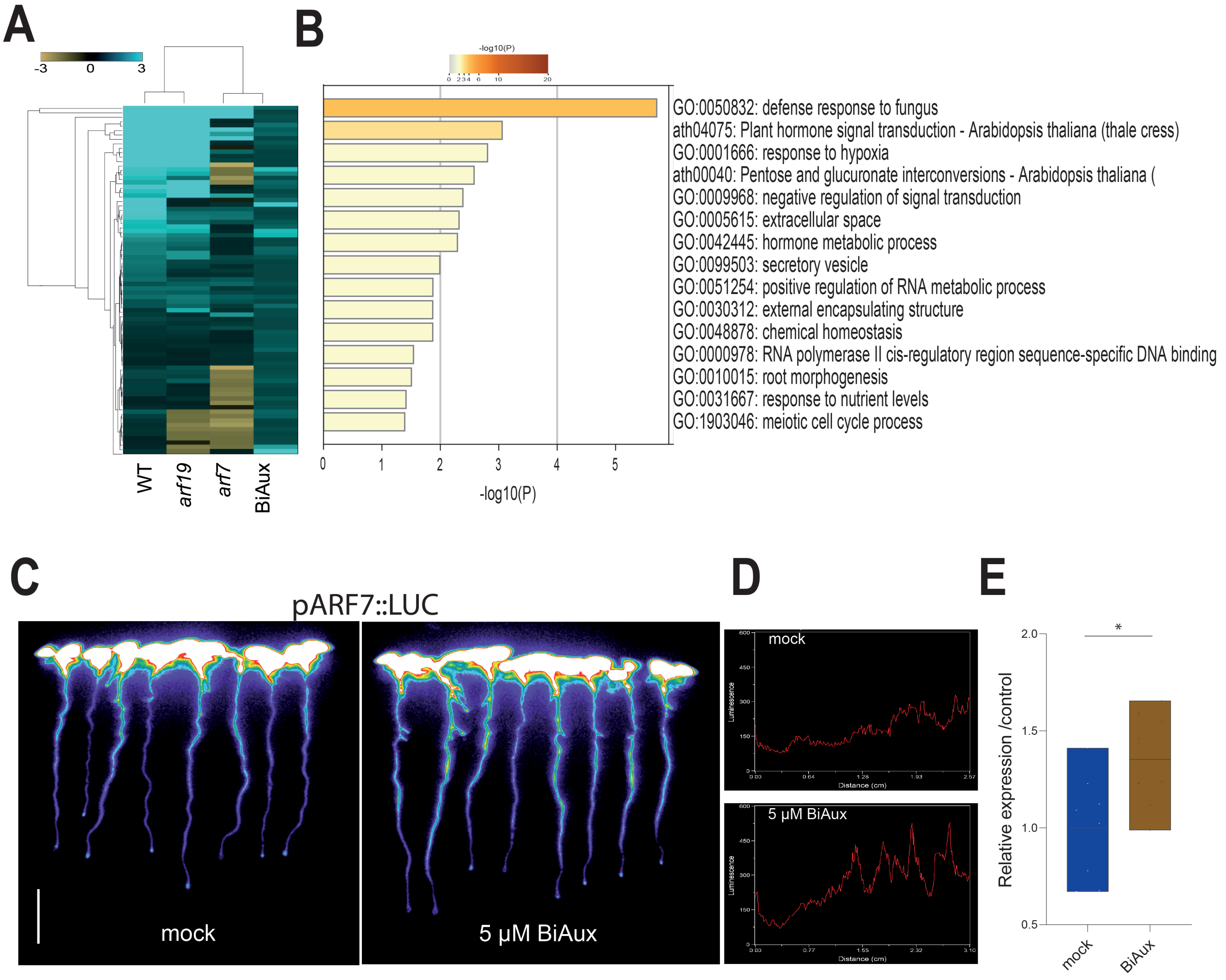

Supplemental Fig. S8: BiAux signaling is mediated by ARF7. A) Hierarchical clustering of genes up-regulated by BiAux that were also up regulated in Arabidopsis seedling by auxin and differentially regulated in *arf7* or *arf9* mutants in response to auxin. **B)** Gene ontology of genes that were up-regulated by BiAux in wild type (WT) seedlings and were up-regulated by auxin in WT or *arf19-1,* but not in *arf7-1* mutant. **C)** LUCIFERASE signal in pARF7::LUC Arabidopsis line that was grown 4 days in 1/2MS and then transferred to fresh medium containing mock or 5 µM of BiAux for another 4 days. Scale bar corresponds to 1 cm. **D)** Signal intensity profile (linescan) of the root showing increased *ARF7* expression in pARF7::Luciferase seedlings treated with mock or to 5 µM of BiAux. The 0 corresponds to the quiescent center position. The measures move shootwards. **E)** Quantification of luciferase signal of roots from the panel (C). Asterisk indicate significant differences by t-test. *, p<0.05. n= 4 roots. . Limits in the boxplot correspond to maximum and minimum values. Line inside of boxplot indicates the main.

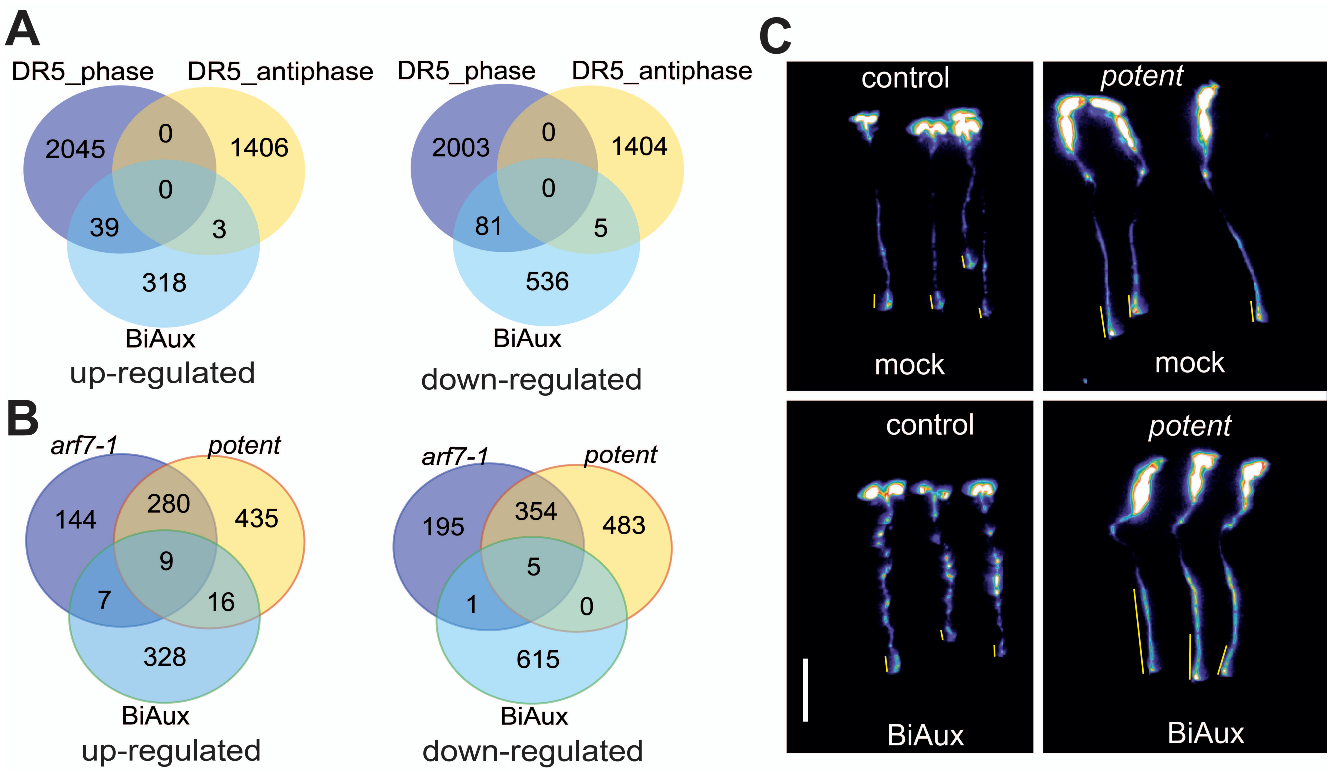

Supplemental Fig. S9: BiAux regulated genes that are preferentially expressed in phase with the DR5::LUC expression in the oscillation zone. A) Venn diagrams showing the common genes between up- or down-regulated genes in BiAux -treated seedlings and genes expressed in DR5-associated phase or antiphase. **B)** Venn diagram of the differentially expressed genes (up- or down-regulated) in WT by the effect of BiAux and deregulated genes (up- or down-regulated) in *arf7-1* or *potent*. **C)** Luciferase assays showing the expression of DR5::LUC marker in control or *potent* seedlings that were grown 4 days in 1/2MS and then transferred to fresh medium containing mock or BiAux during 2 days. Yellow lines indicates the increase in auxin signaling in the meristematic area of DR5::LUC or *potent*/DR5::LUC. Scale bar corresponds to 1 cm.

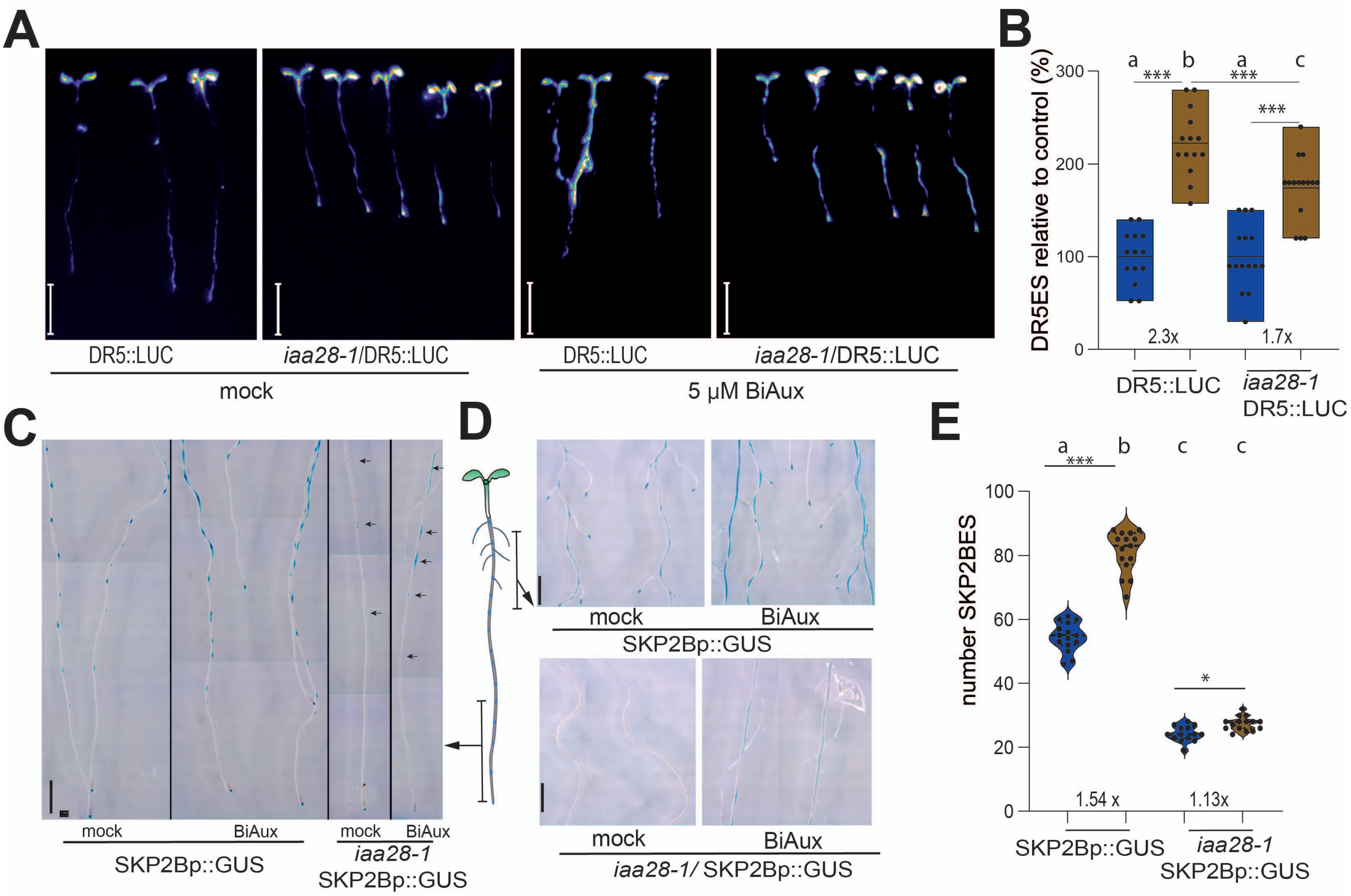

Supplemental Fig. S10: BiAux activity in *iaa28-1* mutant. A) DR5::LUC activity in wild type (Ws ecotype) and iaa28-1 gain-of-function mutant were grown in 1/2MS medium containing mock or 5 µM of BiAux during 6 days. Scale bar corresponds to 1 cm. Images were digitally extracted for comparison. **B)** Relative number of DR5ES measured in the whole DR5::LUC and iaa28/DR5::LUC roots of seedlings grown for 4 days in 1/2MS and transferred to a medium containing mock or 5 µM of BiAux for 3 days. Values represent the percentage relative to the mock average. n≥10 (1 biological replicate). Asterisks indicate significant differences by t-test in a genotype comparing mock and BiAux treatment. ***, pval<0.001. Values at the bottom indicate the fold change increase in both genotypes by the BiAux treatment. **C-D)** GUS staining of SKP2Bp::GUS and iaa28-1/SKP2Bp::GUS roots grown in 1/2MS with mock or 5 µM of BiAux for 12 days, showing the root tip area (C) or a mature region of the root as represented in the cartoon (D). Scale bars correspond to 5 mm. Arrows indicate SKP2BES and several of the root images are composite figures. Note that some of the root images shown are composite figures made of multiple images. **E)** Number of SKP2BES in SKP2Bp::GUS or iaa28-1/ SKP2Bp::GUS grown as in C-D. Different letters indicate statistical differences analyzed by ANOVA and Tukey HSD post-test. Asterisks indicate significant differences by t-test in a genotype comparing mock and BiAux treatment. *, pval<0.05; ***, pval<0.001.

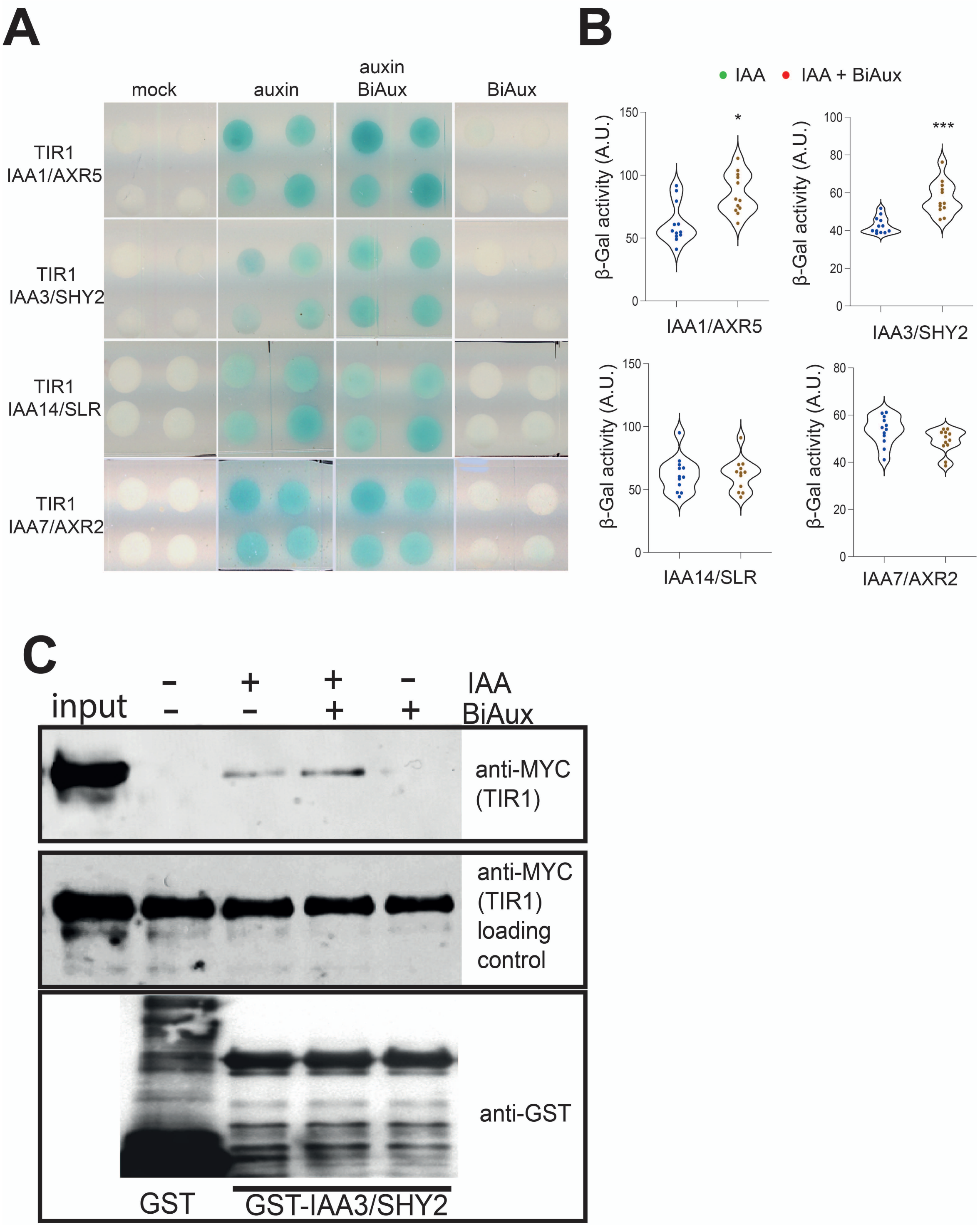

Supplemental Fig. S11: BiAux increases the interaction between TIR1 and specific Aux/IAA proteins. **A)** Yeast-two hybrid interaction between TIR1 and Aux/IAA1, Aux/IAA3, Aux/IAA14 or Aux/IAA7 in presence of auxin (10 μM IAA) with or without 5 μM of BiAux. **B)** Quantification of the interaction shown in A. **C)** TIR1 protein were synthesized *in vitro* and incubated with GST-IAA3 protein in the absence or presence of 10 μM of IAA and 0, or 30 μM of BiAux. After pulldown, the TIR1-myc protein was assessed by western-blot (top panel). Middle panel shows the input of in vitro synthetized TIR1and the lower panel show the GST or GST-IAA3 protein.

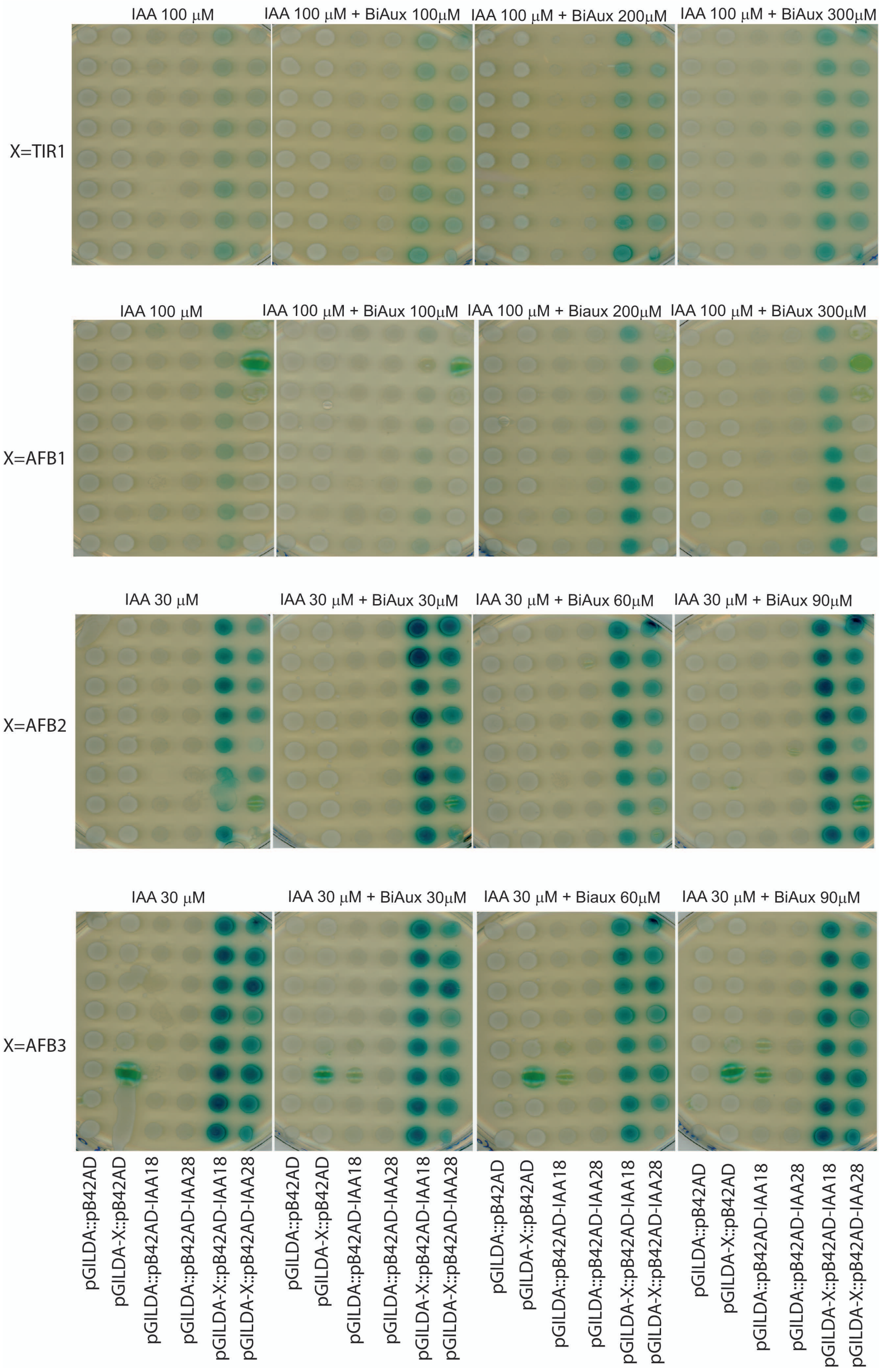

Supplemental Fig. S12. Yeast-two hybrid interaction experiments between TIR1, AFB1, AFB2, or AFB3 with Aux/IAA18 or Aux/IAA28. Yeast harboring GAL4 DBD-TIR1/AFBs and AD-Aux/IAA18 or Aux/IAA28 constructs were generated. Homogenous colonies were spotted on SD-galactose/rafinose inducing medium containing – Ura/–His/–Trp drop out supplement, 80 μg/ml X-Gal, and IAA or BiAux in the indicated concentration. Plates were incubated for 3 days at 30°C and β-galactosidase staining reported the IAA-dependent protein-protein interaction.

Supplemental methods

Plant extraction for mass spectrometry

For each sample, a total of 100 mg of pooled plant roots from seedlings grown with eth root system in presence of light or in darkness were frozen in liquid nitrogen. The plant material was stored at -80 ºC until its use. For the extraction, the plant material was completely transferred into 2 mL screw-cap tubes that contained ceramic beads (MagNA Lyser Green Beads), and 1 mL of a chloroform/methanol/H_2O mixture (20:60:20, v/v) containing 50 µl of a reserpine stock solution (0.2 mg/mL methanol) as internal reference. The plant material was crushed in a MagNA Lyser (Roche, Mannheim, Germany) at 6,500 rpm for 1 min. Next, the samples were centrifuged (10 min, 14,000 rpm) to sediment cell debris, and 200 µl of each supernatant were transferred to 300 µl glass vials and sealed by using screw caps containing PTFE/silicone septa.

BiAux synthesis

Commercially available reagents were used without further purification. Silica gel 40-60 mm, 60 Å was obtained from Acros Organics. Pre-coated silica gel 60 F254 aluminium sheets, Methyl 2-(1*H*-indol-3-yl)acetate, Trifluoroacetic acid (TFA), 2,3-dichloro-5,6-dicyano-1,4-benzoquinone (DDQ), glucose and acetic anhydride were purchased from Merck.

NMR spectra were recorded in $CDCl_3$ or DMSO-d6 (δ=ppm) on a Bruker AV. 250 MHz and a Bruker AV. 400 MHz Spectrometer. Chemical shifts (δ) are reported in parts per million (ppm) relative to $CDCl_3$ (1H: δ 7.27 ppm) and $CDCl_3$ (13C: δ 77.0 ppm) or DMSO-d6 (1H: δ 2.49 ppm)

Scheme 1. General procedure for the synthesis of Biaux.

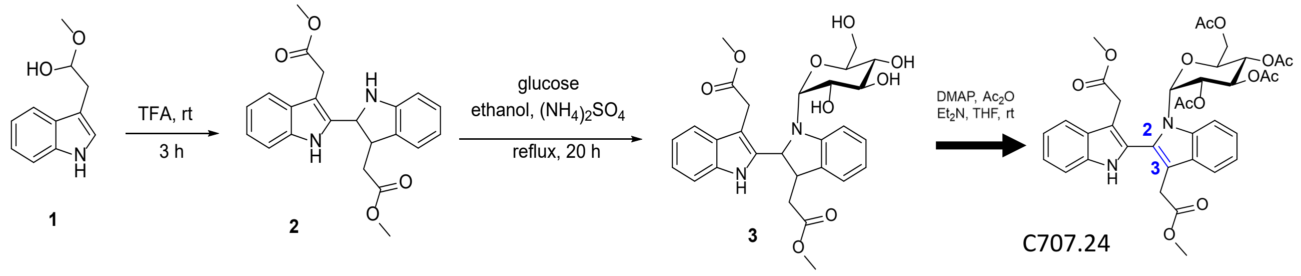

1. Synthesis of methyl 2-(2-(3-(2-methoxy-2-oxoethyl)-1*H*-indol-2-yl)indolin-3-yl)acetate (**2**)

Methyl 2-(1*H*-indol-3-yl)acetate (**1**, 500 mg, 2.64 mmol) was dissolved in 6.6 mL of trifluoroacetic acid and the solution was stirred under Argon atmosphere at room temperature. After 3 h 15 mL of water were added and the pH was adjusted to 4 with NaOH 2N in an ice bath. The product was extracted with dichloromethane (3 x 15 mL). The organic fractions were collected, dried over Na_2SO_4 anhydrous and evaporated under vacuum, affording a light yellow semi-solid (455 mg, 1.20 mmol, 90 % yield). NMR data were consistent with previous reported data. Anal. Calc. for $C_{22}H_{22}N_2O_4$: C, 69.83; H, 5.86; N,7.40. Found: C, 69.72; H, 5.84; N, 7.38. ^{1}H NMR (250 MHz, $CDCl_3$) δ 8.71 (s, 1H), 7.59 (d, *J* = 7.7 Hz, 1H), 7.35 (d, *J* = 7.5 Hz, 1H), 7.25-7.09 (m, 4H), 6.90 (t, *J* = 7.0 Hz, 1H), 6.81 (d, *J* = 7.7 Hz, 1H), 5.07 (d, *J* = 8.2 Hz, 1H), 3.89 (m, 1H), 3.81 (d, *J* = 1.2 Hz, 2H), 3.71 (s, 3H), 3.64 (s, 3H), 2.84 (d, *J* = 6.8 Hz, 2H).

2. Synthesis of methyl 2-(2-(3-(2-methoxy-2-oxoethyl)-1-glucopyranosylindolin-2-yl)-1*H*-indol-3-yl)acetate (**3**)

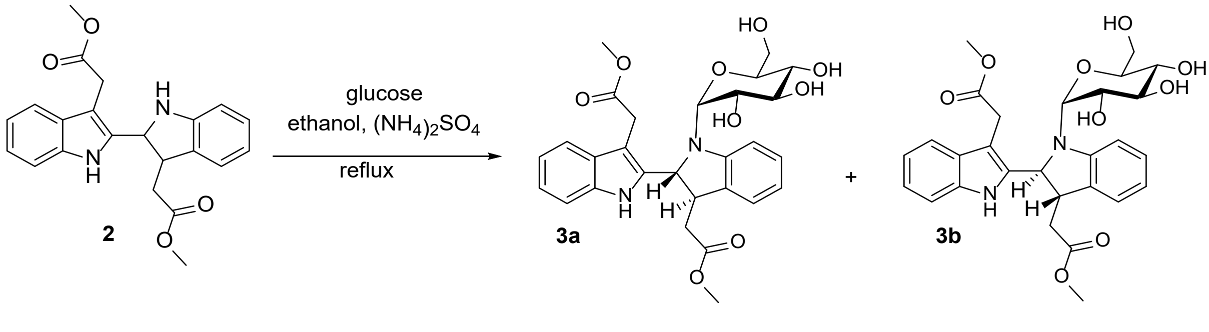

The 2,2´-indolylindoline (450 mg, 1.19 mmol) was dissolved in absolute ethanol (10 mL) and glucose (643 mg, 3.57 mmol) and $(NH_4)_2SO_4$(315 mg, 2.38 mmol) and 3 Å molecular sieves (100 mg) were added. The mixture was refluxed under Argon for 8 h and the reaction progress was monitored by TLC, employing as mobile phase CH_2Cl_2/MeOH (90:10, v,v). The solvent was evaporated under vacuum, and the product was purified through column chromatography, employing silica gel and CH_2Cl_2/MeOH (90:10, v,v) as eluent, to yield a colorless syrup (385 mg, 0.714 mmol, 60 % yield). **3** was collected as a mixture of diasteroisomers **3a** and **3b**. NMR data were consistent with previous reported data.

Anal. Calc. for $C_{28}H_{32}N_2O_9$: C, 62.21; H, 5.97; N, 5.18. Found: C, 61.97; H, 5.94; N, 5.16.

^{1}H NMR (250 MHz, $CDCl_3$) δ 9.09 (s, 1H), 8.96 (s, 1H), 7.54-7.51 (m, 1H), 7.48 – 7.44 (m, 1H), 7.25-7.20 (m, 3H), 7.14-7.05 (m, 7H), 6.90 (dd, J = 14.6, 7.5 Hz, 2H), 6.82 – 6.75 (m, 2H), 5.21 (d, J = 4.9 Hz, 2H), 4.67 (d, J = 8.0 Hz, 1H), 4.47 (d, J = 9.0 Hz, 1H), 3.82 (d, J = 4.0 Hz, 2H), 3.74-3.72 (m, 2H), 3.71-3.69 (m, 6H), 3.66 (s, 3H), 3.64 (s, 6H), 3.60 (s, 6H), 3.56 (s, 3H), 3.45-3.42 (m, 4H), 3.31-3.29 (m, 2H), 3.20-3.18 (m, 4H), 2.74 (t, J = 6.6 Hz, 4H).

3. Synthesis of BiAux (4)

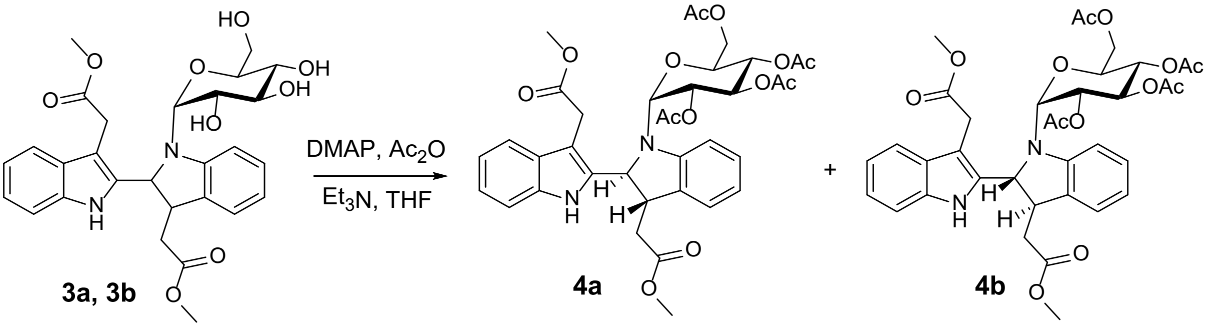

Methyl 2-(2-(3-(2-methoxy-2-oxoethyl)-1-glucopyranosylindolin-2-yl)-1*H*-indol-3-yl)acetate (**3,** 300 mg, 0.554 mmol), dimethlaminopyridine (DMAP, 24 mg, 0.196 mmol) and triethylamine (163 µL,1.16 mmol) were dissolved in 3 mL of freshly distilled THF. Acetic anhydride (245 µL, 2.58 mmol) was added dropwise during 30 min. and the reaction was stirred

at room temperature. After 24 h the solvent was evaporated under vacuum and the product was purified through silica gel-column chromatography, employing a mixture CH_2Cl_2/MeOH (90:5, v,v) as eluent. Biaux was collected as a white solid as a mixture of diasteroisomers **4a** and **4b** (320 mg, 0.454 mmol, 82 % yield).

Anal. Calc. for $C_{36}H_{40}N_2O_{13}$: C, 61.01; H, 5.69; N, 3.95. Found: C, 60.83; H, 5.67; N, 3.93.

^{1}H NMR (250 MHz, $CDCl_3$) δ 8.26 (s, 1H), 7.89 (s, 1H), 7.52 (d, *J* = 1.7, 1H), 7.49 (d, *J* = 2.0, 1H), 7.22 (d, *J* = 1.2, 1H), 7.16-7.09 (m, 4H), 7.06-6.98 (m, 5H), 6.84-6.76 (m, 3H), 6.69 (d, *J* = 7.9 Hz, 1H), 5.39 (t, *J* = 9.4 Hz, 1H), 5.17-5.08 (m, 3H), 5.03-5.01 (m, 1H), 4.96 (d, *J* = 4.9 Hz, 1H), 4.92-4.71 (m, 3H), 4.03 (d, *J* = 2 Hz, 2H), 3.97-3.87 (m, 2H), 3.84-3.73 (m, 4H), 3.65 (s, 3H), 3.63 (s, 3H), 3.59 (s, 3H), 3.56 (s, 3H), 2.65-2.53 (m, 4H), 1.94 (s, 6H), 1.93 (s, 3H), 1.92 (s, 3H), 1.91 (s, 3H), 1,90 (s, 3H), 1.86 (s, 3H), 1.79 (s, 3H).

^{13}C NMR (63 MHz, $CDCl_3$) δ 171.2, 171.2, 170.8, 169.7, 169.2, 168.4, 134.7, 134.4, 129.4, 127.4, 127.1, 126.9, 123.6, 121.5, 121.4, 119.3, 118.7, 118.6, 117.6, 109.9, 85.1, 73.2, 72.7, 71.9, 67.3, 66.8, 60.3, 51.0, 50.7, 45.2, 37.5, 28.8, 19.6, 19.5, 19.3, 19.1.

NMR spectra of Biaux

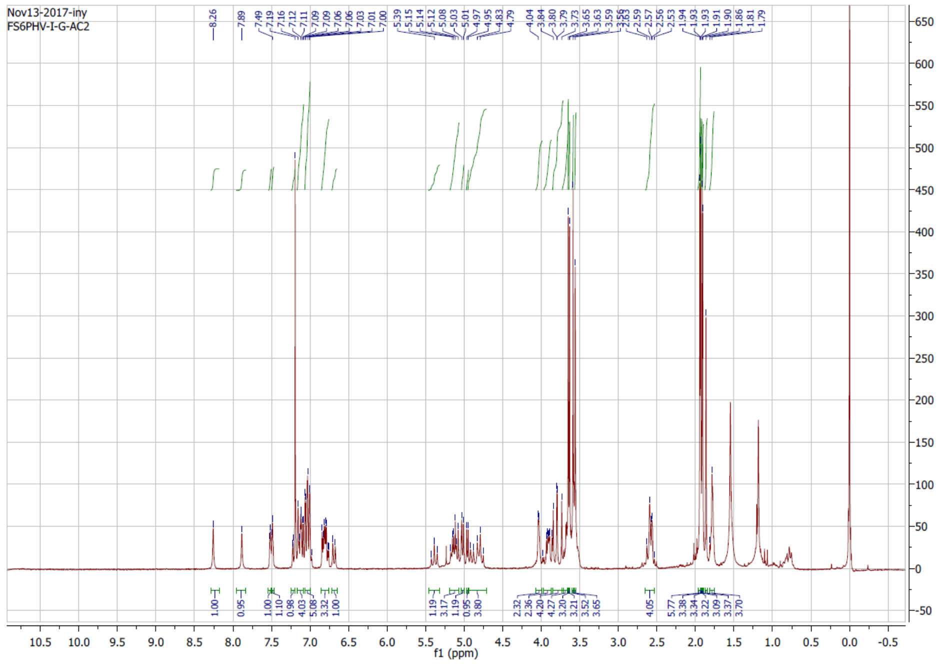

Figure SM1. ^{1}H-NMR spectra of Biaux.

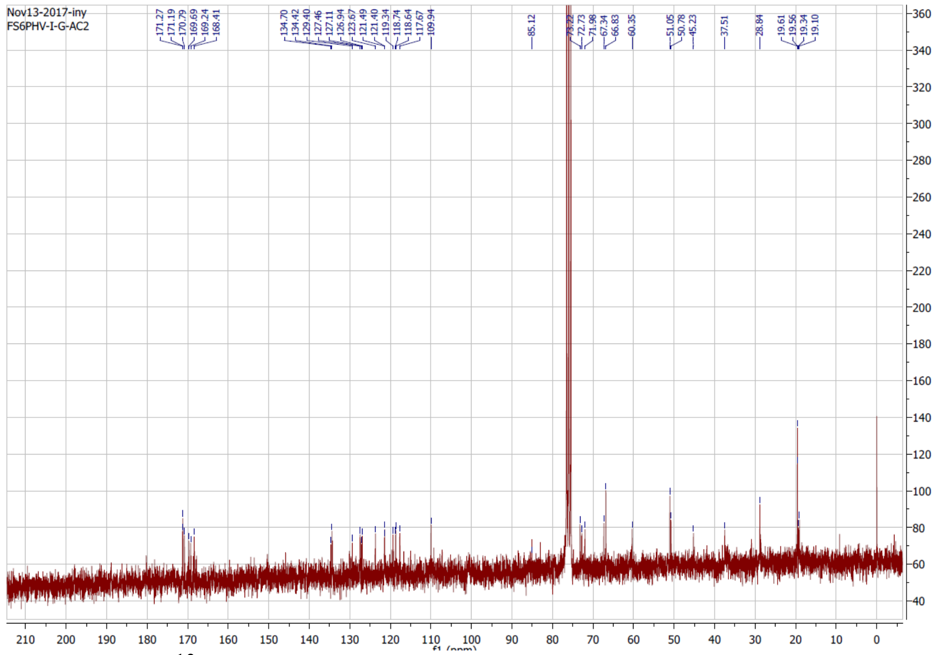

Figure SM2. ^{13}C-NMR spectra of Biaux.

To verify the correct synthesis of BiAux, this was also studied using the MS/MS spectrum (Fig. SM3)

BiAux MS/MS

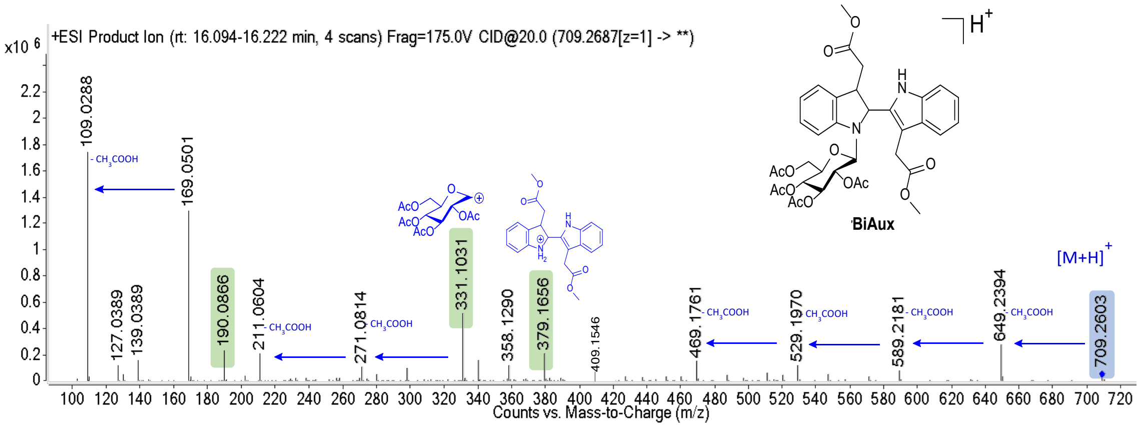

Fig. SM3. MS/MS spectrum for BiAux and fragmentation products.

Confocal laser microscopy

For confocal laser microscopy, we used a Leica SP8 microscopy with the Leica Application Suite (Las AF Lite) X software or a vertical Zeiss LSM 880 with the ZEN 2.3 SP1 software. Roots were stained with propidium iodide (PI) as indicated. To investigate the expression of the different transcriptional or translational fluorescent protein fusions, we used the standard settings for the corresponding green fluorescent protein (GFP), YFP tags.

In Vitro Protein Expression and Pulldown Reactions.

The cDNAs of TIR1 was amplified and cloned into pTNT (Promega) fused to an MYC epitope. Aux/IAA3 cDNAs was amplified and cloned into pGEX-4T3 (Amersham Pharmacia) using EcoRI and XhoI restriction sites. Full length TIR1 protein was obtained by in vitro translation using TNT coupled wheat germ extract system (Promega). GST-tagged Aux/IAA proteins were expressed and purified from Escherichia coli using standard procedures. For pulldown assays, 20 µL of TIR1 protein was incubated for 2 h at 4 °C with µg of GSH-sepharose immobilized

GST-Aux/IAA3 protein in 200 uL of lysis buffer (50 mM Tris p 8.0, 200 mM NaCl, 10% glycerol, 0.1% Tween-20, protease inhibitors) in the presence of 10 μM

IAA. After washing the samples 5 times with 10-fold bed volumes of lysis buffer, beads were resuspended in one bed volume of sample buffer, denaturated, and separated on SDS/PAGE. Products were detected western-blot against MYC (1:7000, Roche) and anti-mouse (1:50.000 Santa Cruz). The GST was detected by western-blot using anti-GST conjugated with HRP (1:30.000) (SantaCruz).

Tomato TIR1 and TIR1 docking analyses

Dockings and molecular structures

3D model structures of tomato TIR1 (UniProtKB: C8C507), TIR1-like_1 (UniProtKB: A0A3Q7F6C2), TIR1-like_2 (UniProtKB: Q6TDU2), amd TIR1-like_3 (UniProtKB: A0A3Q7GSK9) proteins were retrieved from the AlphaFold Protein Structure Database (https://alphafold.ebi.ac.uk/), which since 2022 includes almost all the sequences in UniProt (Varadi et al., 2022). The similarity of these models with the corresponding Arabidopsis models was evaluated with TM-align (https://zhanggroup.org/TM-align/) (Zhang and Skolnick, 2005). This method quantifies the structural similarity between two proteins by computing the TM-score, a unitless parameter defined in the (0 – 1) range, widely employed as a metrics to quantify structural similarity (is that used by the Protein Data Bank). TM-score values smaller than 0.3 mean no similarity, values greater than 0.50 suggest a common fold, and TM-scores approaching 1.0 indicate great overall similarity, with 1.0 meaning perfect match between the structures (Zhang and Skolnick, 2004). Given that the structures of Arabidopsis TIR1 and AFBs proteins were modelled with other methodology, we checked that they were consistent with the AlphaFold models. In fact, TM-scores systematically above 0.95 were found in all cases thus revealing that both methodologies provide essentially the same structures for Arabidopsis sequences. The geometries of BiAux docked to TIR1 and AFBs were obtained with the same AutoDock Vina methodology employed for the equivalent Arabidopsis proteins. Structural superpositions of Arabidopsis and tomato protein models were obtained with the MatchMaker tool of Chimera. The superposition metrics given by root mean square distance (RMSD) values was that provided by MatchMaker.

Phylogenetic analysis

TIR 1nd TIR1-like protein sequences from tomato (*Solanum lycopersicum*) were obtained by blasting Arabidopsis amino acid sequence against the tomato genomic DNA bank (using 6 frames translation). Four different sequences were obtained and the protein sequence was used to generated the protein structure for further docking analyses: Solyc09g074520.3.1; Solyc06g008780.3.1; Solyc02g079190.3.1; and Solyc05g052620.4.1.

The maximum-likelihood phylogenetic tree was based on the alignment of AFB and TIR family protein sequences of *Arabidopsis thaliana* and *Solanum lycopersicum*. The phylogenetic tree was made by MEGA6 software using Bootstrap method with 1000 replications as test of phylogeny and Jones-Taylor-Thornton (JTT) method as analysis model. The length of a branch denotes the genetic distance (number of substitutions per unit time) between the two taxa it connects.

References:

Varadi M, Anyango S, Deshpande M, Nair S, Natassia C, Yordanova G, Yuan D, Stroe O, Wood E, Laydon A, Zıdek A, Green T, Tunyasuvunakool K, Petersen S, Jumper J, Clancy E, Green R, Vora A, Lutfi M, Figurnov M, Cowie A, Hobbs N, Kohli P, Kleywegt G, Birney E, Hassabis D, Velankar S (2022) AlphaFold Protein Structure Database: massively expanding the structural coverage of protein-sequence space with high-accuracy models. Nucleic Acids Research 50: D439-D444.

Zhang Y, Skolnick J (2005) TM-align: a protein structure alignment algorithm based on the TM-score. Nucleic Acids Research 33: 2302-2309.

Zhang Y, Skolnick J (2004) Scoring function for automated assessment of protein structure template quality. PROTEINS: Structure, Function, and Bioinformatics 57: 702-710.

MEGA6: Molecular Evolutionary Genetics Analysis Version 6.0 Tamura K, Stecher G, Peterson D, Filipski A, and Kumar S (2013) Molecular Biology and Evolution 30:2725-2729
