# Supplementary material for: Synthetically derived BiAux modulates auxin co-receptor activity to stimulate lateral root formation
Source: Plant Physiol. 2024 Feb 20;195(2):1694–711. doi: 10.1093/plphys/kiae090 (PMC11142373; doi:10.1093/plphys/kiae090)
Supplement: kiae090_Supplementary_Data [file pp.00900.2023-s07.pdf › pp.00900.2023-s07.pdf]

Plant Physiology®

BiAux modulates auxin co-receptor activity to stimulate lateral root formation

Mary Paz González-García, Ángela Saéz, Monica Lanza, Pilar Hoyos, Estefano ustillo-Avendaño, Luis Pacios, Ana Gradillas, Miguel Moreno-Risueno, Maria Hernaiz, and Juan del Pozo

Review Timeline:

Submission Date:	16-Jun-2023
Editorial Decision:	01-Sep-2023
Revision Received:	29-Nov-2023
Editorial Decision:	30-Dec-2023
Revision Received:	12-Jan-2024
Accepted:	13-Jan-2024

11-Sep-2023

Prof. Juan Carlos del pozo
Centro de Biotecnologia y Genomica de Plantas (CBGP) UPM-INIA/CSIC
biotechnology
Pozuelo de Alarcon, Madrid 28040
Spain

RE: BiAux modulates auxin co-receptors activity to form lateral roots

Dear Dr. del pozo:

We have received reviews of your manuscript, which you may view at the end of this letter. The reviewers have made valuable suggestions that I am sure it will help improving the quality of this manuscript.
On the basis of the advice received and my own evaluation, I believe that, pending revision, this article may ultimately be acceptable for publication in Plant Physiology.

EDITOR INSERT COMMENTS HERE

When you are ready to submit your revision, click the below link and find the red arrow under your Author Tasks heading for this revision submission.

If you want to visit this version of the manuscript, you may click the link below and then click on the folder that reads "Post Decision Manuscripts".

Link Not Available

When uploading the revised version of this article, please be sure to include as complete an answer as possible to the referees' criticisms during the first round of reviews in the "response to reviewers" section.

Plant Physiology considers a revision for up to 60 days after a manuscript has been provisionally accepted; limited extension of this timeframe is at the discretion of the Monitoring Editor. A reminder notice will be sent to you 2 weeks before the deadline, at which time you may choose to contact the editorial office to request an extension; if so, please provide a justification for your request. After the expiration of this deadline, the article would need to be submitted as a resubmission and would be handled as such.

Thank you very much for giving us an opportunity to review this work. I look forward to receiving the next version.

Sincerely,

Plant Physiology Board of Editors

Want to add this revision deadline to your calendar? Click below!

-- Reviewer comments:
Reviewer #1 (Comments for the Author):

34915_Manuscript：
In the manuscript by González-García et al., the authors newly identified a compound that constantly accumulates in illuminated roots, from a non-targeted metabolomic analysis done by the same group at 2016. Since light-grown-roots show more eLRs (data from the paper in 2015, from the same group), they synthesized the BiAux and substantially analyzed its effect to LR

development. They provided convincing phenotypic and molecular evidences to show that application of BiAux increases PBS number and enhances the emergence of LR. In the presence of auxin, BiAux is able to increase the interaction between specific TIR1/AFB2-Aux/IAA combinations and BiAux-induced signaling in LR development requires TIR1 and AFB2. Then the authors further provided the molecular evidences of BiAux in promoting the TIR1/AFB2-IAA18 and TIR1/AFB2-IAA28 interactions, and proposed that BiAux-aided auxin-dependent degradation of IAA18 and IAA28 likely promote the emergence of LR and FC specification respectively.

However, I have a concern about the "PBS". According to my understandings, the PBS defined in the manuscript includes pre-branch sites in the OZ, specified FC and perhaps LRP? I could not find an accurate definition in the M&M. So, this makes another confusing point, the increase of PBS indicated in the manuscript could be due to the change of oscillatory DR5::LUC time period (which I didn't see any evidences provided in the manuscript), or the conversion of pre-branch sites to FC (which is regulated by the IAA28-GATA23 signaling module), or the combination of both. If the authors could carefully clarify/explain the scenario, or add new evidences of DR5::LUC oscillatory period in the presence of BiAux, this manuscript will be more accurate. There are some other minor issues, please find them below:

Comments:
1. Line 17: ...expression and metabolite accumulation, such as flavonoids (Silva-Navas et al., 2016).
> Please add also the one from 2015 (D-Root), the one with LR data.

2. Line 85: ...finally acetylation of the sugar (Fig. 1A and Supplemental Methods)...
>Fig. 1A to Fig. 1B

3. Line 87: instead of 2,3-dichloro-5,6-dicyano-1,4-benzoquinone (DDQ) (Fig. 1A; Supplemental Methods)...
>Fig. 1A to Fig. 1B

4. Line102: ...vasculature (Supplemental Fig. S2C-D). We also...
> Fig. S2C-D to Fig. S2A

5. Line 104: ...nor the SKP2Bp::GUS expression (Supplemental Fig. S2E-G).
> Fig. S2E-G to Fig. S2B-D

6. Line 105: To answer this, we analyzed the effect of BiAux in forming PBS by treating DR5::LUC seedlings with different concentration.
> For your consideration: "To answer this, we compared the effect of BiAux in forming PBS with the effect of IAA, by treating DR5::LUC seedlings with different concentrations of BiAux or IAA."

7. Line 110 and Line 806-808: ...in Supplemental Fig. S3B-C...
>In Fig. S3C, how did you collect the data? Did you cut the roots into pre- and post-transfer parts and count the eLR and PBS? If it is like this, there should be no PBS sites in the shoot-ward pre-transfer region... (the y-axis label in the upper left bar chart, PBS+eLR?)?
Or you counted the eLR+PBS at 4d time point and then counted them again at 7d time point? If it is in this way, please indicate clearer in the figure legend.

8. Line 119: ...meristem but reduced the size meristematic cells...
>...meristem but reduced the length of meristematic cells...

9. Line 140: ...In the case of slr1, BiAux weirdly led to the...
>Does this mean BiAux "cannot" or "can" led to the emergence of LRs in slr1?

10. Line 161: We found that the double treatment deregulated similar genes that IAA alone, but affecting the expression level of some of those IAA-regulated genes.
>For your consideration: "The double treatment deregulated the expression of the genes originally not affected by the BiAux treatment alone, resulting a DEG gene list similar to the one from the IAA treatment alone, but the double treatment altered the expression level of some IAA-regulated genes."

11. Line 220: We found a significant overlap between BiAux-regulated and phase-regulated transcripts (Supplemental Fig. S8A),...
>For your consideration: "We found a significant overlap between BiAux-regulated and in-phase expressed transcripts (Supplemental Fig. S8A),...".

12. Line 235-237: Mechanistically, we found that BiAux enhanced IAA18/POTENT interaction with TIR1, AFB2 and AFB1 in the yeast two hybrid system (Fig 8A-B, Supplemental Fig. S9), likely leading to increase the number of PBS.
>I am confused about the logic in this part, please revise it:
1. potent mutant contains a dominant mutation in conserved DII domain, an auxin insensitive version. In this mutant, in-phase

genes and DR5::LUC are persistently expressed, partially through the repression of ARF7 activities (so in both potent and arf7 loss-of-function mutant, even the oscillation is thought to be blocked, the DR5::LUC marked PBS number is increased). However, here if BiAux enhance the interaction between IAA18 and TIR1 and 2 AFBs, IAA18 proteins will be degraded (and released the ARF7), which will likely to decrease DR5::LUC-marked PBS number. This is contradictory to your statement about PBS.
2. As you mentioned previously that: "...which would be in agreement with previous observations in which LR formation can be activated in potent upon auxin treatment". If I understand correctly, here you meant auxin is able to let the LRs to be emerged/developed (the process after the FC is specified) in potent mutants, then it is a process different from PBS formation. BiAux is able to induce extended DR5::LUC expression above the OZ in potent, meaning that it might promoter emergence/development of LRs. Then the BiAux share or partially share the function of auxin in promoting LR emergence/development in potent. So, what is the correlation between this point and the point you stated in the next sentence?

13. Line 338-339: proposed that BiAux promotes FC specification and LR formation by enhancing the auxin dependent degradation of IAA28 by TIR1 and AFB2.
>Is this mean that the increased number of PBS, induced by BiAux, is contributed by the promotion of FC specification, rather than the change of oscillation itself? I didn't see the time-lapse experiment recording the oscillatory time period of DR5::LUC activities.

14. Line 429: The number of PBS was determined through the quantification of the number of DR5::LUC sites with high expression relative to the adjacent regions along the primary root.
>Can you please indicate more specifically here which region of the root did you use to measure the PBS? In OZ, or from basal meristem to the early DZ? Or along the whole root? If it is the whole root, is it mean the number of PBS also include sites with FC and LRP?

15. Line 738: ...or down-regulated (c) genes in root Arabidopsis seedlings treated with mock or BiAux during 8 days.
> ...or down-regulated (c) genes in Arabidopsis roots treated with mock or BiAux during 8 days.
>The statement of "during 8 days" is different from your description in M&M, line 467-469.

16. Line 770-772: Please remove it.

17. Line 813: b) Cell size of the first 31 cortical meristematic cells...
> b) Cell length of the first 31 cortical meristematic cells...

Fig. 6A, 7A: Scale bar is missing.

Fig. S2C: Scale of the scale bar is missing.

Fig. S3A-B, S6D, S7C, S8C, S10A: Scale bar is missing.

Fig. S11B: The diameter of the coin...

Reviewer #2 (Comments for the Author):

The manuscript by Gonzalez-Garcia presents the identification of a light-inducible metabolite, and the characterization of a reduced variant thereof on lateral root development, named BiAux. While exogenous application of BiAux induces LR development, it has little effect on the primary root. This effect is dependent on specific elements of the canonical auxin signaling pathway, and seems to depend on effects of BiAux on IAA-mediated interactions between TIR1-AFB2 and a subset of Aux/IAAs. This raises a very interesting hypothesis that endogenous metabolites could directly and modify selectively auxin signaling outputs. I appreciate that this research is in the discovery phase, focussing on its initial characterization, and thus that questions related to the endogenous function of BiAux via mutants and evolution of BiAux biosynthesis are beyond the scope of this manuscript.

I do have some suggestions for improvement of the manuscript
Major
- Update introduction with role of adenylate cyclase activity of TIR1 and co (cfr Qi et al 2022), and mention ABP-TMK based signaling that also can impact on transcriptional auxin responses. Briefly, mention AFB1, CNGC14 and Ca^{2+} signaling to highlight functional diversification of TIR1/AFB co-receptors.
- Introduce the ability to selectively activate TIR1/AFB-Aux/IAA pairs via synthetic auxin analogs (Vain et al DOI: 10.1073/pnas.1809037116)
- An unknown molecule with m/z 709 Da was identified endogenous, but a reduced variant of the candidate molecule was characterized in the manuscript. This feature was only mentioned briefly. It remains however unclear to what extent this reduced version has the same bioactivity as the endogenously detected molecule? Moreover, a better chemical characterization of both molecules should be included to allow to better estimate how good BiAux approximates the endogenously detected molecule. I

find it particularly important that the authors elaborate more on the consequences of their choice to work with this reduced version, and what could be the impact on the interpretation of the results of bioactivity of the endogenous molecule.
- Line 105: it is asked if BiAux could release IAA. However, only a very indirect experiment was performed, based on DR5::LUC. IAA release should be measured biochemically
- BiAux was only bioactive at relatively high concentrations. How does this compare to the amount of non-reduced BiAux that was detected endogenously?
- BiAux was proposed to increase LR emergence. Does this involve increased auxin signaling within and around developing LR primordia (eg using DR5::VENUS details of different LRP stages?
- The paragraph line 169-177 seems out of place. It deals briefly with effects on TIR-Aux/IAA interaction, a topic that comes back towards the end of the manuscript. I found this highly confusing, and would suggest to integrate all similar data points (potent and iaa28 data too) into one coherent section.
- The conclusion about a biological function of the neutral electrostatic potential around the binding site in TIR1 and AFB2 is not well supported. Now it is mainly based on the DR5::LUC phenotype of tir1afb2. I would suggest to soften the conclusions associated with such experiments. Or provide additional functional data based on complementation assays and domain swaps, ...
- Line 232: it would be good to compare the effect of BiAux with IAA and IAA + BiAux
- Throughout the manuscript the effects of BiAux are based on Y2H assays. These data would require support via alternative approaches, such as biochemistry and demonstrate the in planta relevance on Aux/IAA stability using LUC fusions to quantify degradation rates (cfr Vain et al).
- While the non-reduced BiAux was identified by comparing illuminated and non-illuminated roots, not much was done link back the importance of BiAux to LR formation in these conditions.

Minor
- Line 23: crystal resolution -> crystal structure
- Line 42: IAA-conjugated -> IAA-conjugate
- Line 56: ARF9 -> ARF19?
- Line 110: none IAA concentrations -> none of the IAA concentrations
- Line 137: none LRs -> no LRs?
- Line 140: What is meant with " In the case of slr1, BiAux weirdly led to the emergence of LRs, but significantly increased the number of PBS stained for SKP2Bp::GUS activity". Do you mean, it led to the formation of LRs (which is unexpected in slr-1), or you meant to write, it did not lead to formation of emerged LRs, but significantly increased the number of PBS stained for for SKP2Bp::GUS activity?
- Line 153: glucosynolate -> glucosinolate?
- Line 161: "double treatment deregulated similar genes, that IAA alone" -> I think it should be than IAA alone, but then the "similar genes" still does not really make sense. Please amend/rephrase
- Line 278: that Arabidopsis genome -> that the Arabidopsis genome
- Line 288: it was showed -> it was shown
- Fig 3D: it is unclear which genotype was quantified, or why not both WT and slr-1?
- Fig 3: Care should be taken to chose the correct statistical test. T-tests is not well suited for LR counts.
- Fig 4D: What is rBiAux?
- Define n as number of roots and number of independent experiments
- Add scale bars to all figures containing plant pictures
- Fig 7D: DR5:LUC should be DR5::LUC
- A differential effect via TIR1 and AFB2 is proposed? It would be of interest to see how deeply this electrostatic effect is conserved? Eg. in tomato, where BiAux was also detected
- The conclusion in line 112 about BiAux requiring the entire molecule is poorly supported by the data presented in the corresponding section, and thus requires softening
- It is interesting to see that the primary root was slightly longer, and that the root meristem cell size and number was changed, without changing the root meristem size. How does the root length then increase. Due to an increased cell production rate? How is the final cell length? Given the proposed link between cell proliferation and elongation as determinants auxin oscillation frequency and amplitude (Van den bergh et al), I was wondering if these parameters would fit, or contradict the aforementioned model?
- As BiAux promotes FC specification, is root clock amplitude or frequency changed?
- In the paragraph 131-134: short term treatments are used. Yet, these "short-term" treatments encompass time-frames of 6h to 120h. This is arguably short-term, but rather long-term. Consequently, this also hampers the interpretation of BiAux doing something at the level of early auxin signaling.

Madrid, 22 November 2023

Answer to reviewers:

BiAux modulates auxin co-receptors activity to form lateral roots

Dear Dr. del Pozo:

We have received reviews of your manuscript, which you may view at the end of this letter. The reviewers have made valuable suggestions that I am sure it will help improving the quality of this manuscript.

On the basis of the advice received and my own evaluation, I believe that, pending revision, this article may ultimately be acceptable for publication in Plant Physiology.

Dear Editor,

First, I want to thank you and the reviewers for the positive and constructive comments to improve our article. In this new version, we have answered almost all their request and concerns, including the reorganization of the data suggested by reviewer 2. We have included the effect of BiAux on the root clock as well as the promotion of IAA28 degradation.

-- Reviewer comments:

Reviewer #1 (Comments for the Author):

34915_Manuscript：

In the manuscript by González-García et al., the authors newly identified a compound that constantly accumulates in illuminated roots, from a non-targeted metabolomic analysis done by the same group at 2016. Since light-grown-roots show more eLRs (data from the paper in 2015, from the same group), they synthesized the BiAux and substantially analyzed its effect to LR development. They provided convincing phenotypic and molecular evidences to show that application of BiAux increases PBS number and enhances the emergence of LR. In the presence of auxin, BiAux is able to increase the interaction between specific TIR1/AFB2-Aux/IAA combinations and BiAux-induced signaling in LR development requires TIR1 and AFB2. Then the authors further provided the molecular evidences of BiAux in promoting the TIR1/AFB2-IAA18 and TIR1/AFB2-IAA28 interactions, and proposed that BiAux-aided auxin-dependent degradation of IAA18 and IAA28 likely promote the emergence of LR and FC specification respectively.

First, we would like to thank the reviewers for the careful and insightful review of the manuscript and the constructive comments and suggestions made to improve the manuscript. I agree with the reviewer that the use of PBS and founder cell in the manuscript should be clarified.

However, I have a concern about the "PBS". According to my understandings, the PBS defined in the manuscript includes pre-branch sites in the OZ, specified FC and perhaps LRP? I could not find an accurate definition in the M&M. So, this makes another confusing point, the increase of PBS indicated in the manuscript could be due to the change of oscillatory DR5::LUC time period (which I didn't see any evidences provided in the manuscript), or the conversion of pre-branch sites to FC (which is regulated by the IAA28-GATA23 signaling module), or the combination of both. If the authors could carefully clarify/explain the scenario, or add new evidences of DR5::LUC oscillatory period in the presence of BiAux, this manuscript will be more accurate.

We completely agree with the reviewer. We did not define correctly in each experiment. To make it more understandable, we have defined the meaning of both DR5::LUC and SKP2Bp::GUS markers. We used DR5::LUC to analyze the oscillation zone and formation of new PBS. We also used it to count expression sites along the root. Thus, in this case, the number of PBS (as defined in Moreno-Risueno 2010, and Van Norman et al., 2013) counted as DR5::LUC expression sites, correspond to PBS + LRP.

On the other hand, we used the SKP2Bp::GUS to analyze and quantify founder cells and LRP. When we counted all the SKP2B expression sites (SKP2BES), this number will correspond to FC and LRP. This information has been included in m&m and in results section.

To answer your question about the effect of BiAux on the root clock, we performed new experiments. We found that BiAux treatment significantly increases the duration of the oscillation period, forming to closer PBS. In addition, a slight increase in the root clock amplitude (DR5::LUC signal) was observed in BiAux-treated roots. Interestingly, all the PBS formed showed a much higher DR5::LUC expression than mock roots, expression that was maintained over the time (see new Fig. 5). These data suggest that in BiAux treated roots almost all PBS will form LRP while in mock treated roots some PBS will be transformed as a reservoir (DR5 off). In addition, data in Fig. 2D, reinforce the idea that BiAux modifies the root clock, since BiAux did not change root growth but increased the number of SKP2B::GUS expression sites.

There are some other minor issues, please find them below:

Comments:

1. Line 17: ...expression and metabolite accumulation, such as flavonoids (Silva-Navas et al., 2016).

> Please add also the one from 2015 (D-Root), the one with LR data.

This has been added

2. Line 85: ...finally acetylation of the sugar (Fig. 1A and Supplemental Methods)...

>Fig. 1A to Fig. 1B

This has been changed

3. Line 87: instead of 2,3-dichloro-5,6-dicyano-1,4-benzoquinone (DDQ) (Fig. 1A; Supplemental Methods)...

>Fig. 1A to Fig. 1B

This has been changed

4. Line102: ...vasculature (Supplemental Fig. S2C-D). We also...

> Fig. S2C-D to Fig. S2A

This has been changed

5. Line 104: ...nor the SKP2Bp::GUS expression (Supplemental Fig. S2E-G).

> Fig. S2E-G to Fig. S2B-D

This has been changed

6. Line 105: To answer this, we analyzed the effect of BiAux in forming PBS by treating DR5::LUC seedlings with different concentration.

> For your consideration: "To answer this, we compared the effect of BiAux in forming PBS with the effect of IAA, by treating DR5::LUC seedlings with different concentrations of BiAux or IAA."

Thanks for the suggestion. It has been modified.

7. Line 110 and Line 806-808: ...in Supplemental Fig. S3B-C...

>In Fig. S3C, how did you collect the data? Did you cut the roots into pre- and post-transfer parts and count the eLR and PBS? If it is like this, there should be no PBS sites in the shoot-ward pre-transfer region... (the y-axis label in the upper left bar chart, PBS+eLR?)? Or you counted the eLR+PBS at 4d time point and then counted them again at 7d time point? If it is in this way, please indicate clearer in the figure legend.

It has been changed in the legend. Seedlings were grown 4 days in 1/2 MS medium and then transferred to 1/2MS medium containing mock, IAA or BiAux. Pre-transfer zone: root portion from shoot-root junction until the line (a portion that has grown in 1/2MS

medium). In this portion, the DR5::LUC markers labels mainly to late stages LRP, but probably also some arrested PBS that can be marked between LRP (Manzano et al., 2012). Post-transfer zone: root portion from line to the root tip. This post-transfer area has been grown in 1/2MS medium containing mock, IAA or BiAux, and in this case DR5::LUC labels new generated PBS+LRP.

8. Line 119: ...meristem but reduced the size meristematic cells...
>...meristem but reduced the length of meristematic cells...
This has been changed.
9. Line 140: ...In the case of slr1, BiAux weirdly led to the...
>Does this mean BiAux "cannot" or "can" led to the emergence of LRs in slr1?
We only found 1 LR in 1 plant out of 12. Thus we should say: "cannot led to the emergence" It has been changed in the text.

10. Line 161: We found that the double treatment deregulated similar genes that IAA alone, but affecting the expression level of some of those IAA-regulated genes.
>For your consideration: "The double treatment deregulated the expression of the genes originally not affected by the BiAux treatment alone, resulting a DEG gene list similar to the one from the IAA treatment alone, but the double treatment altered the expression level of some IAA-regulated genes."
Thank you for the suggestion. It makes it clear. The text has been modified.
11. Line 220: We found a significant overlap between BiAux-regulated and phase-regulated transcripts (Supplemental Fig. S8A),...
>For your consideration: "We found a significant overlap between BiAux-regulated and in-phase expressed transcripts (Supplemental Fig. S8A),...".
It has been changed.
12. Line 235-237: Mechanistically, we found that BiAux enhanced IAA18/POTENT interaction with TIR1, AFB2 and AFB1 in the yeast two hybrid system (Fig 8A-B, Supplemental Fig. S9), likely leading to increase the number of PBS.
>I am confused about the logic in this part, please revise it:
1. potent mutant contains a dominant mutation in conserved DII domain, an auxin insensitive version. In this mutant, in-phase genes and DR5::LUC are persistently expressed, partially through the repression of ARF7 activities (so in both potent and arf7 loss-of-function mutant, even the oscillation is thought to be blocked, the

DR5::LUC marked PBS number is increased). However, here if BiAux enhance the interaction between IAA18 and TIR1 and 2 AFBs, IAA18 proteins will be degraded (and released the ARF7), which will likely to decrease DR5::LUC-marked PBS number. This is contradictory to your statement about PBS.

The reviewer's assumption is correct, POTENT blocks ARF7, and this is necessary to label a PBS. However, these root clock components form part of a circuit, where IAA18 at one point must be degraded. Thus, BiAux, combined with auxin, might accelerate such degradation and prompts the next PBS-activation, reducing the frequency and increasing the number of PBS and later the formation of FC and LRP. The latest has been demonstrated using FC-marker SKP2B::GUS in Fig. 2D. In addition, as suggested by the reviewer 2, the higher cell number in the meristem of BiAux-treated plants might lead to an increase in PBS formation. Thus, one tentative hypothesis is that BiAux is connecting cell proliferation in the meristem and root clock via IAA18-ARF7 to generate PBS, and IAA28-GATA23 module to transit from PBS to Founder Cell. However, additional experiments should be done.

2. As you mentioned previously that: "...which would be in agreement with previous observations in which LR formation can be activated in potent upon auxin treatment". If I understand correctly, here you meant auxin is able to let the LRs to be emerged/developed (the process after the FC is specified) in potent mutants, then it is a process different from PBS formation.

Yes, it is a different process. For LR development, the ARF7 activity is needed, as *arf7 arf19* mutant does not develop any LR (Okushima et al. Plant Cell. 2007 Jan; 19(1): 118–130. doi: 10.1105/tpc.106.047761) while for PBS specification, ARF7 activity has to be blocked by IAA18 (Perianez-Rodriguez et al., Sci. Adv. 2021; 7 : eabd4722, doi: 10.1126/sciadv.abd472). Later, BiAux favors IAA28 degradation to prompt the specification of FC by releasing ARF transcription factors that activate GATA23, which is necessary to form founder cells from PBS. This degradation, together with the increase of *ARF7* expression, enhances the formation of LRP. This has been suggested by the higher number of SKP2B::GUS expression sites, a FC and LRP marker, after BiAux treatment (Fig 2D).

BiAux is able to induce extended DR5::LUC expression above the OZ in *potent*, meaning that it might promoter emergence/development of LRs.

We showed that, in the *potent* mutant, in which IAA18 is not degradable, BiAux increased the DR5::LUC expression and probably the PBS specification. We think this

is due to the inability to degrade IAA18 protein and the probable induction of *IAA18* by BiAux. However, conversely to auxin effect, we did not observe a massive LR formation in *potent* after BiAux treatment (see figure above).

Then the BiAux share or partially share the function of auxin in promoting LR emergence/development in potent. So, what is the correlation between this point and the point you stated in the next sentence?

In our analyses, when we added BiAux to *potent* for 3 days, we did not observed the massive emergence of LR as was found with 0.25 µM of IAA (Perianez-Rodriguez et al., Sci. Adv. 2021; 7 : eabd4722). We only found a larger DR5::LUC oscillation zone (SFig 8C), suggesting that BiAux enhances POTENT-ARF7 activity in the OZ. Furthermore, based on the experiments presented in Fig 2D, we think that BiAux also promoted LRP formation.

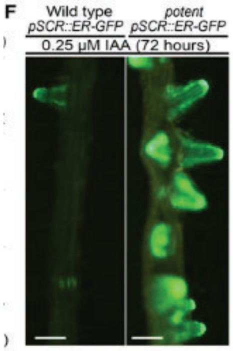

Fig. 1 The IAA18/POTENT factor regulates the root clock oscillations and LR priming.
(F) Altered positioning of LRs in iaa18/potent mutant. (G) Quantification of LR formation in iaa18/potent, in iaa18/potent allele (IAA18P102L) expressed under its regulatory regions, and in an alternative mutant allele (crane2). (A to D) Seven days post imbibition (dpi).
From (Perianez-Rodriguez et al., Sci. Adv. 2021; 7 : eabd4722)

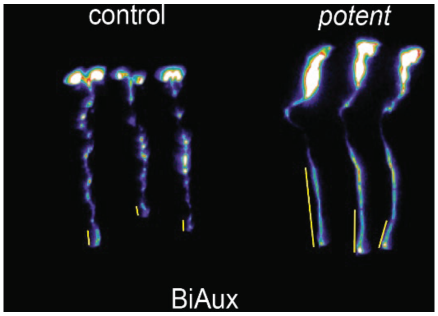

Supplemental Fig S8. BiAux regulated genes that are preferentially expressed in phase with the root the oscillation zone. C) Luciferase assays showing theexpression of DR5::LUC marker in control or *potent* seedlings that were grown 4 days in 1/2MS and thentransferred to fresh medium containing mock or BiAux during 2 days. Yellow lines indicates the increasein auxin signaling in the meristematic area of DR5::LUC or *potent*/ DR5::LUC.

13. Line 338-339: proposed that BiAux promotes FC specification and LR formation by enhancing the auxin dependent degradation of IAA28 by TIR1 and AFB2.

We have now showed that BiAux enhanced IAA28 protein degradation.

>Is this mean that the increased number of PBS, induced by BiAux, is contributed by the promotion of FC specification, rather than the change of oscillation itself? I didn't see the time-lapse experiment recording the oscillatory time period of DR5::LUC activities.

We have now carried out a time-lapse during 24 hours. We found a slight increase in the root clock amplitude in BiAux-treated roots. However, we found that BiAux

significantly increase the duration of the period, forming to close PBS. In addition, all the PBS formed showed a higher DR5::LUC expression than mock roots, suggesting that all PBS will form LRP later (see new Fig. 5). In addition, data in Fig. 2D, reinforce the idea that BiAux modifies the root clock, since BiAux did not change root growth but increased the number of SKP2B::GUS expression sites.

14. Line 429: The number of PBS was determined through the quantification of the number of DR5::LUC sites with high expression relative to the adjacent regions along the primary root.
>Can you please indicate more specifically here which region of the root did you use to measure the PBS? In OZ, or from basal meristem to the early DZ? Or along the whole root? If it is the whole root, is it mean the number of PBS also include sites with FC and LRP?
Thank you for bringing out this point. The measurement was done along the root and therefore, DR5::LUC marker labels PBS+FC+ LRP. Thus, in agreement with the reviewer, they should not be considered strictly PBS, but we decided to follow the definition made by Moreno-Risueno et al., 2010; Van Norman et al., 2013; i.e. DR5::LUC expression sites along the root. In this case, these PBS correspond to cells primed during the oscillations and LRP.

15. Line 738: ...or down-regulated (c) genes in root Arabidopsis seedlings treated with mock or BiAux during 8 days.
> ...or down-regulated (c) genes in Arabidopsis roots treated with mock or BiAux during 8 days.
>The statement of "during 8 days" is different from your description in M&M, line 467-469.
Thank you for this observation. It was a mistake, it corresponds to 4 day in 1/2MS and then transferred to 3 days in a medium containing mock or BiAux. It has been changed.

16. Line 770-772: Please remove it.
It has been removed.

17. Line 813: b) Cell size of the first 31 cortical meristematic cells...
> b) Cell length of the first 31 cortical meristematic cells...

It has been changed

Fig. 6A, 7A: Scale bar is missing.

Fig. S2C: Scale of the scale bar is missing.

Fig. S3A-B, S6D, S7C, S8C, S10A: Scale bar is missing.

Fig. S11B: The diameter of the coin...

We included the scale bars in all figures.

Reviewer #2 (Comments for the Author):

The manuscript by Gonzalez-Garcia presents the identification of a light-inducible metabolite, and the characterization of a reduced variant thereof on lateral root development, named BiAux. While exogenous application of BiAux induces LR development, it has little effect on the primary root. This effect is dependent on specific elements of the canonical auxin signaling pathway, and seems to depend on effects of BiAux on IAA-mediated interactions between TIR1-AFB2 and a subset of Aux/IAAs. This raises a very interesting hypothesis that endogenous metabolites could directly and modify selectively auxin signaling outputs. I appreciate that this research is in the discovery phase, focussing on its initial characterization, and thus that questions related to the endogenous function of BiAux via mutants and evolution of BiAux biosynthesis are beyond the scope of this manuscript.

I do have some suggestions for improvement of the manuscript

Major

- Update introduction with role of adenylate cyclase activity of TIR1 and co (cfr Qi et al 2022), and mention ABP-TMK based signaling that also can impact on transcriptional auxin responses. Briefly, mention AFB1, CNGC14 and Ca^{2+} signaling to highlight functional diversification of TIR1/AFB co-receptors.

Thank you for the suggestion. Definitely, it will improve the manuscript. This has been included in the introduction.

- Introduce the ability to selectively activate TIR1/AFB-Aux/IAA pairs via synthetic auxin analogs (Vain et al DOI: 10.1073/pnas.1809037116).

Thank you for the suggestion. This and other work using antagonist have been introduced.

- An unknown molecule with m/z 709 Da was identified endogenous, but a reduced

variant of the candidate molecule was characterized in the manuscript. This feature was only mentioned briefly. It remains however unclear to what extent this reduced version has the same bioactivity as the endogenously detected molecule? Moreover, a better chemical characterization of both molecules should be included to allow to better estimate how good BiAux approximates the endogenously detected molecule. I find it particularly important that the authors elaborate more on the consequences of their choice to work with this reduced version, and what could be the impact on the interpretation of the results of bioactivity of the endogenous molecule.

Thank you for raising this point. In the first instance, we synthesized the 707.24 metabolite as described in the original manuscript. Later we synthesized BiAux using an easier, eco-friendly and robust procedure. We already carried out different analyses to demonstrate that both 707.24 and BiAux had similar bioactivity (now included in SFig S1C-E). In addition, both compounds increased the root system in tomato, suggesting that both have similar bioactivity in plants.

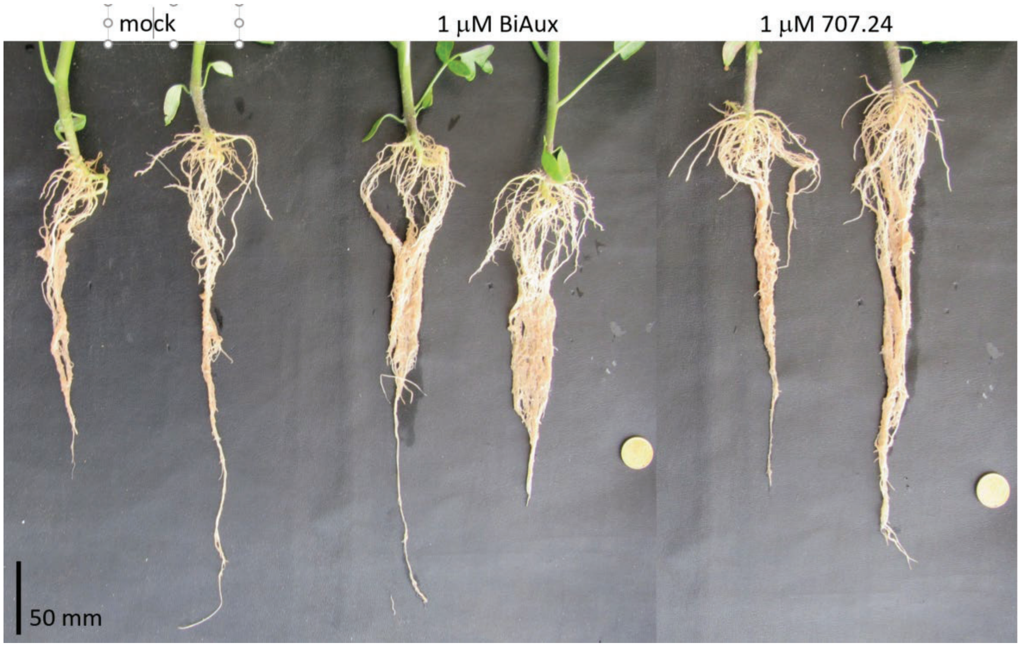

Fig R1_1: Eight weeks tomato plants grown on perlite. They were irrigated wit 0.1x MS solution containing mock or 1 µM of BiAux or 1 µM of C707.24 once a week.

- Line 105: it is asked if BiAux could release IAA. However, only a very indirect experiment was performed, based on DR5::LUC. IAA release should be measured biochemically

We measured the IAA levels in BiAux-treated plants. We could not find any significant difference in IAA accumulation in BiAux treated plants using the whole root. However, we cannot discard that accumulation of IAA occurs in a small group of cells. We think that, although indirect as the reviewer suggested, DR5::LUC induction is a good marker to analyze small increments in auxin concentration. Thus, if IAA would be released from BiAux we should expect an extended DR5::LUC signal along the root. The fact that BiAux only increases DR5::LUC expression in spots associated with LRP, while IAA-treatment increase DR5::LUC expression along the main root . On the other hand, if only 10% of BiAux is transformed into IAA (0.5 µM of IAA), a root growth inhibition should be observed, and this is not the case, even if we used 20 µM of BiAux. Together, all these pieces of information strongly suggest that IAA is not released from BiAux.

In addition, We treated SKP2Bp::GUS, which labels Founder and LRP, with 75 nM IAA, 5 µM BiAux or 75 nM IAA + 5 µM BiAux for 9 hours. As you might see, IAA induces SKP2B along the pericycle and in LRP, while BiAux increases the expression only in founder cells and LRP. Treatment with high concentration of IAA (1 µM) strongly induces SKP2B in pericycle cells and cortex (Fig_R1_2). Thus, IAA in low or high levels does not mimic BiAux effect.

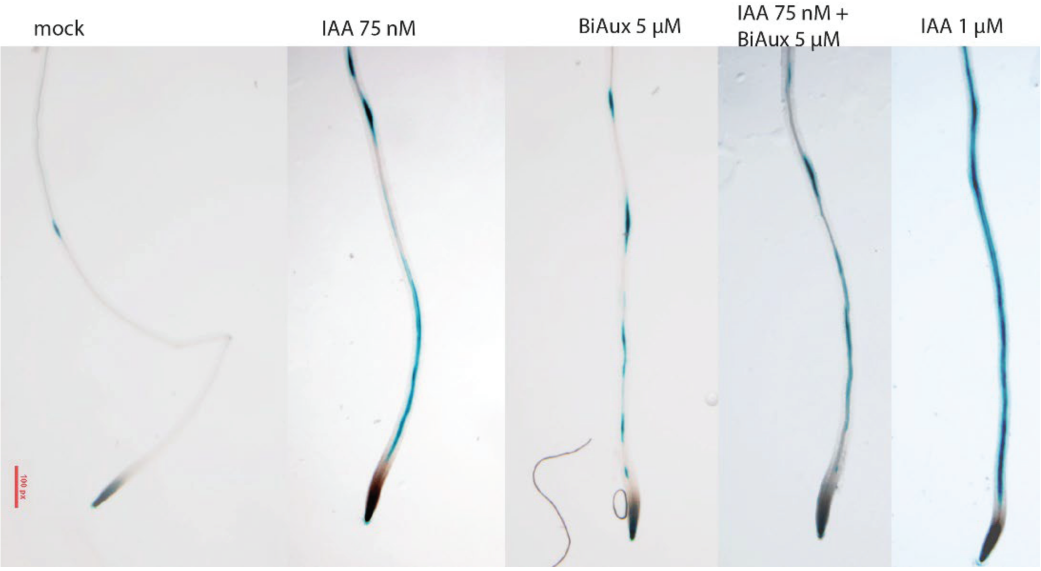

Fig. R1_2: Five day-old SKP2B::GUS seedlings were treated with 75 nM IAA, 5 µM BiAux or 75 nM IAA + 5 µM BiAux or 1 µM IAA for 9 hours and then stained for GUS activity.

- BiAux was only bioactive at relatively high concentrations. How does this compare to the amount of non-reduced BiAux that was detected endogenously?

It is possible that the endogenous molecule accumulated in few cells, probably those that are involved in PBS and FC/LRP formation. That might also explain the low level observed in the metabolomics analyses. We have also done a BiAux concentration assay using the DR5::LUC reporter line, finding that 1 µM induces the formation of DR5-expression sites, although the increase can be seen at 0.25 µM (Fig.R1_3). However, we decided to use 5 µM because we observed much better the different phenotypes described in the paper in wt or mutants seedlings. This assay was not included as SFig to avoid overloading the manuscript.

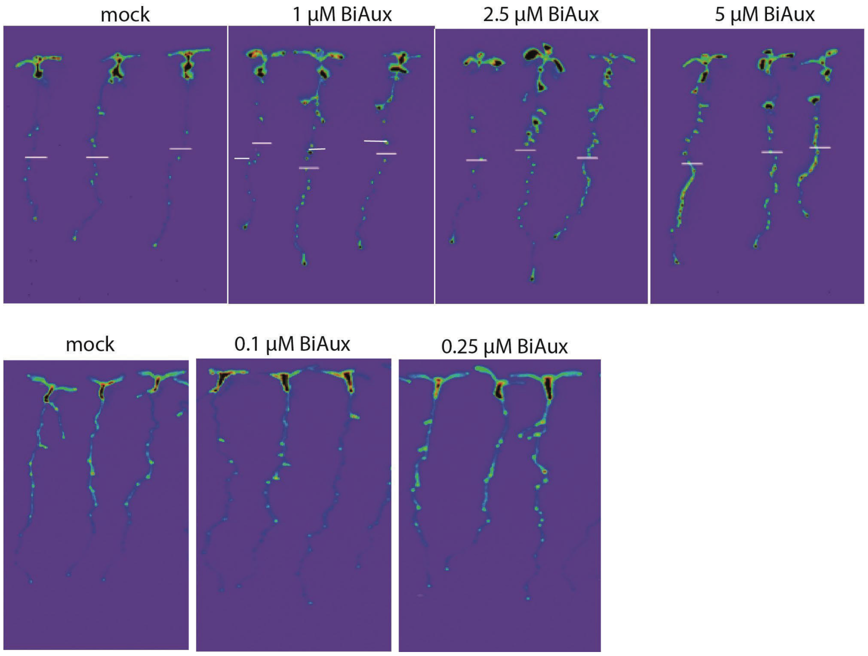

Fig. R1_3: Effect of different concentrations of BiAux. Four day-old DR5::LUC seedlings were transferred to a fresh medium containing mock or different concentration of BiAux. Lower panel corresponds to a second experiment using 0.1 or 0.25 uM of BiAux. Luminescence signal was recorded in the NightOwl. Notice that BiAux effect is dose-dependent.

- BiAux was proposed to increase LR emergence. Does this involve increased auxin signaling within and around developing LR primordia (eg using DR5::VENUS details of different LRP stages?

Using the SKP2B::GUS reporter, we observed higher intensity of GUS staining that covers a larger area surrounding the LRP (Fig. R1_4), suggesting that BiAux triggers initial stages of LRP, probably at the founder cell level, but also increased the expression of LR-markers in later stages, probably to potentiate the emergence. Similar pattern was observed for DR5::GUS.

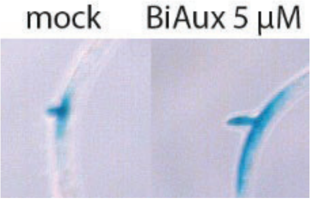

Fig R1_4: expression pattern in emerged lateral root of SKP2B::GUS treated with mock or 5 uM of BiAux for 3 days.

- The paragraph line 169-177 seems out of place. It deals briefly with effects on TIR-Aux/IAA interaction, a topic that comes back towards the end of the manuscript. I found this highly confusing, and would suggest to integrate all similar data points (potent and iaa28 data too) into one coherent section.

We appreciate this suggestion. We have changed and reorganized the results section accordingly.

- The conclusion about a biological function of the neutral electrostatic potential around the binding site in TIR1 and AFB2 is not well supported. Now it is mainly based on the DR5::LUC phenotype of tir1afb2. I would suggest to soften the conclusions associated with such experiments. Or provide additional functional data based on complementation assays and domain swaps, ...

We agree with the reviewer. As we do not have additional data regarding this electrostatic potential we decide to soft the conclusions.

- Line 232: it would be good to compare the effect of BiAux with IAA and IAA + BiAux

We did not present this data since the expression of DR5::LUC was too high and did not help to elucidate the BiAux function.

- Throughout the manuscript the effects of BiAux are based on Y2H assays. These data would require support via alternative approaches, such as biochemistry and demonstrate the in planta relevance on Aux/IAA stability using LUC fusions to quantify degradation rates (cfr Vain et al).

We have tried to analyzed the stability of IAA18-LUC (pIAA18::IAA18-LUC) and we we not able to obtain a conclusive results, since they were slightly different in two independent experiments. However, we show (Fig 9) that BiAux alone (since roots contains endogenous IAA) promoted IAA28-VENUS degradation. This effect was stronger when BiAux and IAA were added together.

- While the non-reduced BiAux was identified by comparing illuminated and non-illuminated roots, not much was done link back the importance of BiAux to LR formation in these conditions.

We previously demonstrated that light is a stress that influence in LR formation and root growth is not a natural condition for roots (Silva-Navas et al., Plant Journal. 2015 Oct;84(1):244-55. doi: 10.1111/tpj.12998 and González-Garcia et al Plant Commun 2023 May 8;4(3):100514. doi: 10.1016/j.xplc.2022.100514) for this we not include any data in the previous manuscript in this direction. However, we have included the effect of BiAux in light- and dark-grown roots, showing that it has a stronger effect in dark.-grown roots, especially in the formation of new PBS (Supplementary Fig 2E).

Minor

- Line 23: crystal resolution -> crystal structure done
- Line 42: IAA-conjugated -> IAA-conjugate done
- Line 56: ARF9 -> ARF19? Thanks, it is ARF19. Changed
- Line 110: none IAA concentrations -> none of the IAA concentrations done
- Line 137: none LRs -> no LRs? Done, thank you.
- Line 140: What is meant with " In the case of slr1, BiAux weirdly led to the emergence of LRs, but significantly increased the number of PBS stained for SKP2Bp::GUS activity". Do you mean, it led to the formation of LRs (which is unexpected in slr-1), or you meant to write, it did not lead to formation of emerged LRs, but significantly increased the number of PBS stained for for SKP2Bp::GUS activity?

I meant: it did not lead to formation of emerged LRs, but significantly increased the number of PBS stained for for SKP2Bp::GUS activity. This has been changed in the text.

- Line 153: glucosynolate -> glucosinolate? changed
- Line 161: "double treatment deregulated similar genes, that IAA alone" -> I think it should be than IAA alone, but then the "similar genes" still does not really make sense. Please amend/rephrase

The sentence has been rephrase for clarity.

- Line 278: that Arabidopsis genome -> that the Arabidopsis genome done
- Line 288: it was showed -> it was shown done
- Fig 3D: it is unclear which genotype was quantified, or why not both WT and slr-1?

It has been corrected in the figure for clarity, since WT corresponded to SKP2Bp::GUS and *slr* is *slr1*/SKP2Bp::GUS

- Fig 3: Care should be taken to chose the correct statistical test. T-tests is not well suited for LR counts. We have rea-analyzed using annova test.
- Fig 4D: What is rBiAux?

Sorry, it is a mistake from previous version where we used that name for reduced BiAux.

- Define n as number of roots and number of independent experiments We have done through the different experiments.
- Add scale bars to all figures containing plant pictures

This has been done. Thank you for pointing it out.

- Fig 7D: DR5:LUC should be DR5::LUC

It has been changed

- A differential effect via TIR1 and AFB2 is proposed? It would be of interest to see how deeply this electrostatic effect is conserved? Eg. in tomato, where BiAux was also detected

By TIR1 blast with the tomato genome, we have identified TIR1-like. Using docking analyses we found that BiAux also binds with different low energy to these proteins and generated differential electrostatic changes (see Supplemental Fig S7).

In addition, we have done docking studies in Physcomitrella and Marchantia. We found that two Physcomitrella TIR1-like proteins binds efficiently BiAux and other two does not. BiAux has effect on Physcomitrella growth. However, in Marchantia, where there I sonly one TIR1-like protein, the binding energy is very low, and BiAux does not seem to have any effect on Marchantia. These data has not been included because are out of the scope of this paper and will be part of a future study.

- The conclusion in line 112 about BiAux requiring the entire molecule is poorly supported by the data presented in the corresponding section, and thus requires softening

We have softened the claim and conclusion.

- It is interesting to see that the primary root was slightly longer, and that the root

meristem cell size and number was changed, without changing the root meristem size. How does the root length then increase. Due to an increased cell production rate? How is the final cell length? Given the proposed link between cell proliferation and elongation as determinants auxin oscillation frequency and amplitude (Van den bergh et al), I was wondering if these parameters would fit, or contradict the aforementioned model?

We checked the cell length in the differentiated zone and not significant differences were found in seedlings treated with BiAux compared with mock. Thus, we think that the slightly root length increase is the consequence of more cell proliferation in the meristem. At this point, we do not have enough data to reliably validate or contradict the proposed model. However, since BiAux mainly increased PBS and LRP, the fact that also modifies cell division in the meristem, generating more cells in a similar area, suggests a possible connection between both processes. It is possible that by having more cell passing through the oscillation zone (OZ) favor the specification of more PBS. This seems to fit in the model. However, it seems that BiAux also influences auxin oscillation periodicity and amplitude in the meristem. Thus, it would be possible that BiAux increases cell division rate in the meristem and, in coordination with auxin, modifies components of the root clock in the OZ to trigger the PBS formation. Although we do not have sufficient data, it is tentative to speculate that BiAux might connects both, cell proliferation, auxin pulse and root clock to specify PBS. Thus, BiAux function might fit in this model. However, additional experiments to answer this exciting question will be needed! Thank you for bringing this idea up.

- As BiAux promotes FC specification, is root clock amplitude or frequency changed?

In the new time lapse experiment that we performed we reveal that the BiAux has an important role in the root clock. We found a slight increase in the root clock amplitude in BiAux-treated roots. However, we found that BiAux significantly increase the duration of the period, forming to close PBS. In addition, all the PBS formed showed a higher DR5::LUC expression than mock roots, suggesting that all PBS will form LRP later (see new Fig. 5). In addition, data in Fig. 2D, reinforce the idea that BiAux modifies the root clock, since BiAux did not change root growth but increased the number of SKP2B::GUS expression sites.

- In the paragraph 131-134: short term treatments are used. Yet, these "short-term"

treatments encompass time-frames of 6h to 120h. This is arguably short-term, but rather long-term. Consequently, this also hampers the interpretation of BiAux doing something at the level of early auxin signaling.

I agree with the reviewer, and we have modified the text. However, using this markers, significant changes can be observed at the same treatment-time than auxin, and therefore it can be speculate that they acts in a similar time-window to trigger some responses. As BiAux seems to modify only LR formation, and does not affect cell elongation, we think it does not participate in the fast auxin response. In addition, we confirmed that BiAux treatment did not activate calcium peaks as fast response.

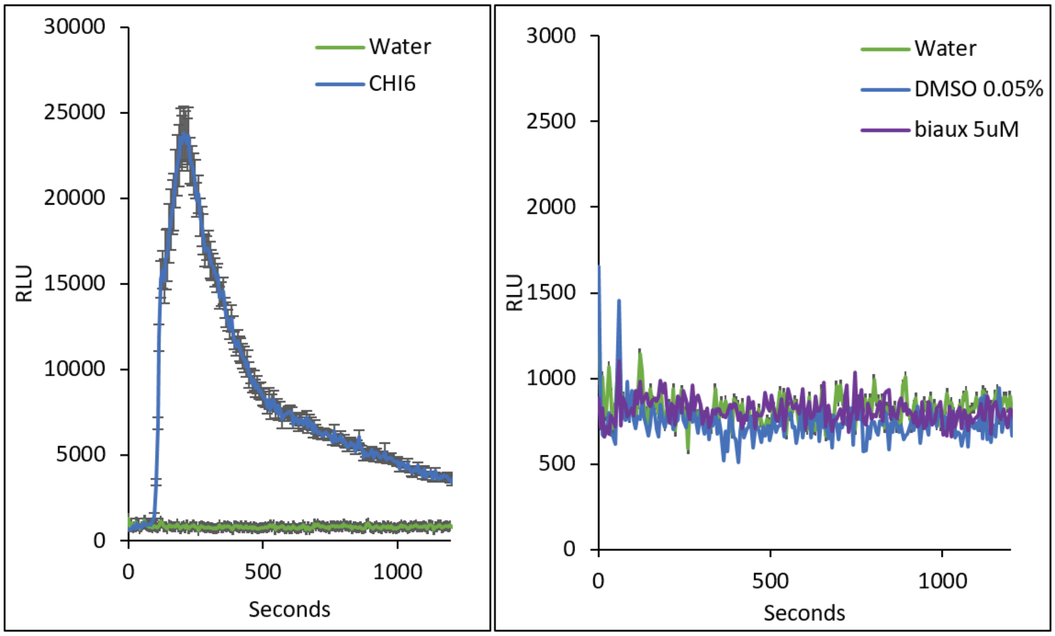

Figure R1_: A. thaliana lines Col-0[AEQ], carrying the calcium reporter aequorin protein, were grown in 96-well plates (with one seedling per well), in half-strength liquid MS medium for 8 days. Medium was removed and added 100 µl of coelenterazine 10 µM in water and incubated o/n. Cytoplasmic Ca2+ influxes were evaluated using a Varioskan Lux Reader luminometer (ThermoFisher Scientific, https://www.thermofisher.com) upon treatment with 50µl of CHI6 50 µM as a positive control or 5uM of BiAux. Measures were taken up to 1000 seconds.

23-Dec-2023

Prof. Juan Carlos del Pozo
Centro de Biotecnologia y Genomica de Plantas (CBGP) UPM-INIA/CSIC
biotechnology
Pozuelo de Alarcon, Madrid 28040
Spain

RE: BiAux modulates auxin co-receptors activity to form lateral roots

Dear Prof. del Pozo, dear Juan:

We are pleased to accept your manuscript for publication in the Plant Physiology. This acceptance is contingent on revision based on the comments of our reviewers. In particular, please consider some annotations from reviewer 1 and minor changes requested by reviewer2.

Please highlight all changes and include a detailed annotation to changes to the text, with line numbers, and noting your responses to the comments.

To submit your revised manuscript, click:

Link Not Available

If you cannot return the revised manuscript within 8 weeks of receipt, please let us know. Otherwise, we will assume that you have elected not to revise the manuscript and are withdrawing it.

Thank you for allowing us to review your work. We look forward to hearing from you soon.

Sincerely,

Plant Physiology Board of Editors

-- Reviewer comments:
Reviewer #1 (Comments for the Author):

Please find the comments in the attaced pdf. file

Reviewer #2 (Comments for the Author):

I applaud the authors for a job well done. All my comments have been addressed.
I just found some type-o's that you might want to take care off:

Title: co-receptors activity -> co-receptor activity. And maybe replace "to form lateral root" -> to stimulate lateral root formation (or development)
Line 151: five old day DR5::LUC -> five day old DR5::LUC
Line 153" Kymographs analyses -> Kymograph analyses
Line 157: more and closed PBS -> more and closer PBS were formed. I would probably rephrase it a bit more, it reads a bit awkward.
Line 181: non LRs -> no LRs
Following Fukaki et al. I would suggest to always write solitary root-1 and slr-1. As the gene is SOLITARY ROOT, but the it is the first mutant allele
Line 300: Aux/IAA1 and Aux/IAA3 should be IAA1/AXR5 and IAA3/SHY2
Line 301: IAA14/SLR1 should be IAA14/SLR

Dear Editor and reviewer,

Thank you for the evaluation and all the suggestions that you have proposed to improve the manuscript. In general, we have accepted all the grammatical corrections because they definitely improve the manuscript.

Reviewer 2: Thank you for your kind comments.

Title: co-receptors activity -> co-receptor activity. And maybe replace "to form lateral root" -> to stimulate lateral root formation (or development) We have modified the title attending your suggestions.
Line 151: five old day DR5::LUC -> five day old DR5::LUC It has been changed.
Line 153" Kymographs analyses -> Kymograph analyses It has been changed.
Line 157: more and closed PBS -> more and closer PBS were formed. I would probably rephrase it a bit more, it reads a bit awkward. We have modified the sentence. I hope now it ease to red.
Line 181: non LRs -> no LRs It has been changed.
Following Fukaki et al. I would suggest to always write solitary root-1 and slr-1. As the gene is SOLITARY ROOT, but the it is the first mutant allele It has been changed.
Line 300: Aux/IAA1 and Aux/IAA3 should be IAA1/AXR5 and IAA3/SHY2 It has been changed.
Line 301: IAA14/SLR1 should be IAA14/SLR It has been changed.

Reviewer 1: Thank you for the enormous work you have done. We have used now the term DR5ES. It is more adequate for static pictures. Below, you will find the answer (red) to specific questions and our view of some subjects.

The revised manuscript has provided the time course analysis to clear the role of BiAux in regulating oscillation and PBS formation. I still have some minor issues listed below,

1. Yes, according to the previous publication, PBS is considered as the static spots with DR5::LUC expression after the OZ. But DR5::LUC expression may not completely reflect PBS spacing, if the experiment was not done in a time lapse way. Because sometimes DR5:LUC expression sites (DR5ES) will be vanished shortly when they pass the OZ (as you also showed in this manuscript). Besides, the DR5 signals in a LR primordium might be also reduced to the level below the detection if this primordium ceased to grow. I would suggest to use DR5ES in the results and figures corresponding to the experiments recorded by photos (and I saw you did in this way in some places).
2. We agree with the referee; in photo, DR5::LUC is a static measurement and sometimes are difficult to assign as PBS. We decided to use PBS because it was named before with this term. Now, following your recommendation we use DR5 expression site (DR5ES) for quantification on static photos, and PBS for the time-lapse analyses.
3. In some of your figures, number of DR5::LUC expression sites were indicated as number of PBS+eLRs, this is confusing (e.g. Fig S3C). It looks like PBS includes the sites with LRFCs and LRP, but not eLRs? According to my understanding, PBS refers to the regions/sites/places that are capable of forming LRP or LRs, so PBS spacing could be reflected by the number of LRFCs+LRP+LRs(eLRs). But I am not sure whether PBS refers to the "fresh" sites with newly formed LRFCs in the early DZ only; or once the sites have been formed, they can be called PBS all the time, no matter where they are (for example they could locate in the most shootward region and possess eLRs). I suppose PBS refers to the second scenario, or not...? Anyway, I recommend to change "PBS+eLRs" to "DR5ES", which is "correct" in any cases...

In the case of Fig S3C we included the emerged LR and PBS to quantify the effect of BiAux or IAA in the pre-transfer zone, since is known that IAA could reactivate "dormant" PBS and also promote the emergence of LRs. Thus, we analyzed both simultaneously to analyze all the BiAux effects. However, we agree with the reviewer and we will call them DR5ES.

4. For those experiments did by transferring seedlings to the BiAux plates (for another several days) in Fig.2C, Fig.4A, Fig.6B and Fig.S10B, please indicate the number of SKP2ES or DR5ES refers to the number counted in the post-transfer root part or the whole root.
   Thanks for the suggestion. This has been indicated. They correspond to the whole root.

5. Does your counting of "relative PBS" include eLRs, as those also marked by DR5::LUC?

   No, in those experiments did not included eLRs (for that reason was not indicated in legend). eLRs are counted if it is indicated.

6. For the PBS formation and LRFC specification these 2 processes, I am not sure if PBS formation induces FC specification. Or alternatively these 2 processes go together, in other words, FC specification is the process or is involved in the process to converge the broadly expressed DR5::LUC region in OZ to a static DR5::LUC point in early DZ. So, for the comments in Line 292-293, please make a decision according to your own view.

   According to our view, PBS formation does not automatically induce FC specification, since some PBS can be arrested or switch-off before transformed to LRFC. That is the reason we used also SKP2B::GUS marker, to follow those that moves to LRFC. However, it is possible that FC also express the DR5 marker, due to auxin signal activation. In the sentence 292-293, we want to indicate that BiAux promotes the transition from PBS to FC, likely because maintains higher level of auxin activity (like by active degradation of specific Aux/IAAs), and the PBS are not switched-off.

Comments:

As I said, the majority of the corrections have been included now in the new version.

Abstract
The root clock periodically specifies lateral root (LR) prebranch sites (PBS), where a group of pericycle founder cells is are primed to become LR founder cells (FCs) and eventually give rise to lateral root primordia (LRP) or LRs. This clock-driven organ formation process is tightly controlled by through modulation of auxin...; ...the understanding of how specific auxin c- receptors co-receptors are assembled remains unclear.

Introduction
Line 6-9:
The root clock in Arabidopsis integrates an oscillating gene expression and auxin signaling to, approximately every 6 hours, specify a new prebranch site (PBS), which marks the region competent for the future emergence of a lateral root primordium.

- I saw in your abstract, PBS is defined as prebranch sites (plural form), please keep it consistent. We used PBS for plural and prebranch site for a single one.

2. Line 11-12:
   ...one is in phase with the expression of the auxin-response reporter *DR5::LUCIFERASE* (*DR5::LUC*) and the other one is in antiphase.
3. Line 13-17:

   The AUXIN RESPONSE FACTOR 7 (ARF7) and INDOLE-3-ACETIC ACID 18 (IAA18)/POTENT transcription factors control this oscillatory circuit to determine prebranch sites (PBS) (Perianez-Rodriguez et al., 2021). Furthermore, based on the epistatic effect of *iaa28-1* mutation, a mutation in the DII domain causing auxin insensitivity, on IAA18/ARF7 mediated PBS spacing, it has been proposed that IAA28 signaling might control the amplitude of the oscillations in the oscillation zone (OZ) by altering auxin responses necessary for the formation of PBS (Perianez-Rodriguez et al., 2021). IAA28 also controls founder cell (FC) specification through the IAA28-GATA23 module.

4. Line 18-23:
   Later, and upwards from the basal meristem, FCs at the PBS are activated to initiate nuclei migration and anticlinal cell division, forming an incipient lateral root primordium (LRP). This initiation process requires the activity of IAA14/SOLITARY-ROOT (SLR)-mediated auxin signaling (Guseman et al., 2015). As the primordium develops, it eventually breaks through the overlaying tissues, as an emerged LR (eLR).

- LRP is usually defined as a plural form. We have used as a plural term.

5. Line 29:
   ...subsequently degraded through the...
6. Line 32:
   During land plant evolution, the TIR1/AFBs and Aux/IAA gene family has expanded, with 6 and 29 members respectively in Arabidopsis.
7. Line 38-42:
   ...trigger auxin responses. Auxin-trigged transcriptional responses mainly rely on the canonical $SCF^{TIR/AFB}$-Aux/IAA-ARF signaling in the nucleus; while those rapid responses are usually regulated by non-transcriptional pathways, for instance the cytosolic $SCF^{TIR/AFB}$- CNGC14 (the plasma-membrane-localized Ca^{2+} channel) pathway to mediate calcium flux and the cell surface AUXIN-BINDING PROTEIN 1 (ABP1)-TRANSMEMBRANE KINASE 1 (TMK1) signaling to induce ROP GTPase activation, cell wall acidification and protein phosphorylation.
8. Line 46:
   ...but not for triggering calcium transient peaks required for...
9. Line 48-49:
   ..., ABP1-LIKE PROTEIN 1 (ABL1) and ABL2, have been shown to interact with..., showing overlapping and distinct functions with ABP1.

10. Line 50:
Despite these advances made in auxin perception, some questions are still open...

11. Line 53:
...serves as a building block for a multitude of...and is considered as one of the most...

12. Line 57:

...or sugars and well as by methylation...

13. Line 59:

    ...that needs an esterase to...

14. Line 60-62:

    ...or altering the root system architecture (?).... Different agonists and antagonists have been...

15. Line 71:

    ...number of PBS and eLRs without...

- You have defined eLR before.

16. Line 73:

    ...auxin, and a set of specific genes...

17. Line 78-80:

    ...different electrostatic potential on TIR1 and AFB2 from those in on (?) AFB1 or AFB3. In addition, genetic functional analyses show that... In agreement with these observations...

18. Line 84:

    ...BiAux enhances the interaction between auxin and specific auxin co-receptors to activate downstream signaling and exert a fine-tune...

➢ Results

1. Line 88:

    BiAux regulates LR formation

2. Line 92-93:

    Among several peaks with increased levels (Silva-Navas et al., 2016), we focused on the one corresponding to the metabolite of LC-ESI(+) MS m/z of 707.2459. Because it was...

3. Line 98-101:

    (http://www.chemspider.com/), through which we found that only 4 out of them matched with already known molecules. Among these 4 candidates, we noticed a compound...and this compound was previously...

4. Line 102:
   As auxin regulates root growth and LR formation, the processes...
5. Line 110:
   ...(Supplemental Fig. S1B and Supplemental Methods). We therefore termed this product as BiAux.
6. Line 112:
   ...the dimer, were detected.
7. Line 115:
   ...was confirmed by LC-ESI-MS analyses and the result showed two peaks that correspond to diasteroisomers 4a and 4b (Supplemental Fig. S1B).
8. Line 116:
   [Please make a new paragraph here, otherwise it is a bit overloaded for the readers] Then, we tested whether the C707.24 and BiAux share any similar biological activities.
9. Line 118:
   ...maker line that labels LRFCs, lateral root primordia (LRP) and eLRs, ...

- LRP are usually refer to the ones that do not grow out from the overlaying tissues.

10. Line 125:

Furthermore, BiAux treatment specifically increased SKP2B expression mainly in FCs and LRP...
- I saw expanded SKP2B expression in Fig. S1C at more shootward region, so it might be safer to use mainly, instead of only.

We have considered this suggestion. It is true that the GUS staining can diffuse depending on the amount of expression.

Line 127-130:
...a marker used to reflect root oscillatory activities, were cultivated in light-grown root (LGR) or...than the BiAux-treated LGR seedlings.
- DR5::LUC may not capable of marking some of the arrested LRP.

> Yes, In this case it would be difficult to know if in LGR there are more arrested LRP that do not express DR5::LUC. Thus we have included this sentence: "although we cannot discard that root illumination favor LRP arrest that do not express DR5."

11. Line 133-134:
    ...we found that BiAux increased the number of DR5::LUC expression sites (DR5ES) at 0.25 µM, and this effect increased in a dose dependent manner...
12. Line 137:
    ...concentrations mimicked the BiAux-treated phenotype in terms of root length or the spacing of DR5ES.
13. Line 138-140:
14. Although we cannot rule out the possibility that BiAux can be modified inside of a specific

    subset of plants cells, our data suggest that the entire BiAux molecule might be specifically required to promote the formation PBS and eLRs (since you defined LRs as eLRs in this manuscript), possibly by modulating the root clock activity.

15. Line 143:
    ...significant longer root system (total root length of the primary root plus eLRs) (Fig. 2A- B).
16. Line 145-147:
    ...cells. Therefore, the meristem size between the BiAux- and mock-treated roots was similar...developed a significantly higher number of SKP2BES and eLRs than the number in non-treated seedlings...
17. Line 149-150:
    As BiAux specifically increased the number of DR5ES, we decided to analyze whether it affects the root clock, which regulates PBS formation...
18. Line 153-155:
    Kymographs analyses showed that in both mock- and BiAux-treated roots, DR5::LUC expression oscillated about 4 times per every 24 hours (Fig. 3A-C).
19. Line 155-157:
    However, in BiAux-treated seedlings, LUC activities in the oscillations were generally higher than the activities detected in mock-treated seedlings (Fig. 3A-B), suggesting an increased amplitude of the oscillations. As a result of these altered LUC activities in the OZ, more and closed PBS were formed in BiAux-treated roots (Fig. 3A, D).
20. Line 161:
    Taken together, these data indicate that BiAux increased the amplitude of the oscillations. As a result, more pericycle cells might be primed.
21. Line 170-171:
    ...significantly higher than in the mock treatment after 72 hours (Fig. 3F), ...favors FC specification in PBS.
22. Line 173:

...suggesting that it promotes the development of previously specified but arrested FCs and/or LRP.
- There are a portion of arrested LRP along the main roots. BiAux may also promote the development of those one. We agree with the reviewer.

24. Line 174:
    ..., these data suggest that BiAux promotes FC specification and LR development.
25. Line 176:
    First, we found that, compared to the mock treatment, BiAux treatment increased the number of DR5ES in wild type roots to a similar level observed in IAA-treated roots up to 48 hours after the treatment, and to a higher level than the IAA treatment at 120 hours.
26. Line 178-182:
    ...auxin-response mutants defective in LR formation, ...(*axr1-12*), which has fewer eLRs, the quadruple auxin receptor mutant..., but in those developed ones, no eLRs are observed, and *solitary root 1* (*slr1*), which does not produce LRP...
27. Line 188:
    ...auxin signaling related to PBS formation, ...
28. Line 192-193:
    ...the expression of 360 genes and reduced the expression of 622 genes. Gene ontology (GO) analyses...
29. Line 198-201:
    We found that although BiAux and IAA deregulated a significant set of common genes, there was still a group of genes solely respond to the BiAux treatment. Interestingly, GO category of root development was found by analyzing the up-regulated gene list form this group, suggesting that Arabidopsis root may differentially respond to the BiAux treatment from the IAA treatment. This may explain the distinct phenotypes observed between the BiAux- and IAA-treated roots (Fig. S3).

30. Line 205:
    ...decreased) their expression levels.

- Only several genes' expression level has been changed? Or most of them?

31. Line 206-209:
    ..., clustering analyses identified two clusters of genes (number 4 and 8), the upregulation of which was enhanced in the BiAux+IAA treatment, compared to the level in the IAA treatment alone (Supplemental Fig. S5B). In both clusters, an enrichment in genes related to hormone signaling and isoprenoid biosynthesis was found, suggesting that BiAux may boost the endogenous IAA response.
32. Line 214-215:
    Based on the phenotype of BiAux-treated plants and the fact that BiAux contains and auxin structure, it would be possible that BiAux affects to auxin signaling by modifying the activity of the auxin...
33. Line 218-219:
    ...number of DR5ES in wild type and the single mutants...a significant reduction of in the number of DR5ES in response to the BiAux treatment in the double...order mutants that include this...
34. Line 223:

...and estimate the binding... 35. Line 225:

...near the binding sites of IAA and... (IP6 or InsP6?)

- I saw in your figure legend, you used IP6. We decided to use IP6

36. Line 232:

    Therefore, we hypothesized that BiAux stabilizes TIR1/AFB-Aux/IAA interaction to increase

    auxin perception...

37. Line 242:

    ...tomato TIR1 and slightly higher? for the TIR1-like proteins compared with the

    Arabidopsis... Thanks for this annotation!

38. Line 256:

    ..., while their expression in...

39. Line 258:

    ...of these BiAux-upregulated and ARF7-dependent genes show...

40. Line 262:

    ...was required to increase the number of DR5ES in response to...

41. Line 267:

DR5::LUC expression oscillates in phase...

42. Line 274-284:

...sequestering ARF7 (Perianez-Rodriguez et al., 2021). DR5::LUC signals in the oscillation zone and the number of PBS in *potent* mutant, an Aux/IAA DII domain mutant, and loss-of- function *arf7-1* mutant are enhanced (Perianez-Rodriguez et al., 2021). The overlap between BiAux-regulated genes with *potent*- and...Interestingly, among these genes...which is also involved in LR formation (Zhang et al., 2021). We found that BiAux treatment...the oscillation zone in *potent* mutant, suggesting that...in this mutant (Supplemental Fig. S9C). This would be...

- Here I move the sentence in line 278-279 in front.

43. Line 286-288:

...the *iaa28-1* mutation, a mutation in the DII domain, generates...wild type and *iaa28-1* mutants increased the number of DR5ES in response to...

44. Line 290:

..., we used the SKP2Bp::GUS marker to analyze the formation of LRFCs in *iaa28-1* roots in response to the BiAux treatment...

45. Line 292-293:

This gain-of-function mutation strongly reduced the number of SKP2BES, suggesting that the transition from PBS to FC promoted by BiAux depends on IAA28 degradation. [I am not sure if you can draw this conclusion] We agree that with this data we cannot fully draw this conclusion. We have soften the statement: “suggesting that the formation of LRFCs promoted by BiAux depends on IAA28 degradation.”

- - I saw more SKP2BES in BiAux-treated *iaa28-1* roots in both C and D: in C, you have a continuous root region stained with blue in the most shootward part (the right pic, BiAux treated), and you did not put arrows to point them out. BiAux seems also increased the number of SKP2BES in BiAux-treated *iaa28-1* roots. Yes, you are right. This has been corrected and indicated in the new version.

- Is the PBS formation earlier than FC specification? Because in OZ, DR5::LUC signals cover a broader area, then these signals converged to one point/site. This observation is interesting, and we think that the DR5 expression takes place in many cells in the OZ, but only those that reach a threshold are primed to become PBS. Later, these PBS eventually become LRFC; and we think that this process might be also depended on the auxin-response intensity. In this sense, BiAux seems to maintain higher level of the auxin-signaling and therefore more LRFCs are specified and LR are formed. I am not sure how this process (PBS formation) is accomplished? FC specification might go together with PBS formation. The

use of “the transition from PBS to FC” makes me feel like you believe that PBS formation is achieved earlier than FC specification. We think that FC process occurs after PBS.

- - If FC specification goes together with PBS formation, it would be reasonable to see an increased relative number in both DR5ES and SKP2BES in BiAux-treated iaa28-1 roots (compare to the mock). But the statistic of SKP2BES is missing, so it is hard to judge.
- - If you have the statistics on hand, it would be good to add, otherwise please consider to make another way to conclude.

  We have now included the number of SKP2BES (Fig S10E). As you might see, the increase in LRFC in wild type plants in about 1.5-fold while in iaa28 mutant is only 1.13-fold. Annova test analyses showed that none differences between mock and BiAux treatment in iaa28 mutant. However, a t-test shows significant differences (pval=0.0189). According to our data, PBS formation seems to be less affected than LRFC formation in *iaa28* mutant in response to BiAux. Thus, taken together, we think that PBS priming occurs in the OZ and the LRFC formation takes place later during development.

46. Line 303-304:
    ...to analyze if it regulates IAA18/POTENT and IAA28, two proteins involved in PBS and LR formation and their stability is...
47. Line 310: We analyzed the role of...

➢ Discussion

1. Line 327:

   ...the number of PBS and LRP and promotes the emergence of...

2. Line 390:

   ...for specifying PBS...

3. Line 396:

   ...leads to the formation of massive PBS in the pericycle...

4. Line 398-399:

   ...the DR5::LUC expression in the OZ, ... active degradation of IAA18/POTENT in roots upon BiAux and/or IAA treatment?, although...need to be done to clarify this point.

5. Line 421:

   ... induce PBS formation, FC activation and LR emergence in...

6. Line 423:

   ... that will transit through the OZ.

7. Line 426:

   ...combined with the modulation of the oscillation amplitude, promote the...

➢ Materials and Methods: 1. Line 436-439:

This line was also used to quantify the total number of PBS, as defined in (Moreno-Risueno et al., 2010; Van Norman et al., 2013), i.e. detectable DR5::LUC expression sites along the root. In this case, total number of PBS corresponds to the sites with primed pericycle cells during the oscillations (did you also count the sites in OZ?), LRFCs, LRP and eLRs?

- Did you include eLRs in your counting? I am a bit confused, or you only count the number of LRP+LRFCs?? If it is not indicated, we only include PBS and LRP.

2. Line 453:
   ...they were mounted them on glass slides and covered with...
3. Line 521-523:
   The number of DR5::LUC expression sites (DR5ES) with high expression relative to the adjacent regions along the primary root was used to approximately reflect PBS spacing. The values were represented as relative (percentage) to the number of DR5ES in the...

- For the measurements using photos, it would be more accurate to say DR5ES? We have used the term DR5ES

4. Line 534-536: Is this the method for Fig. S8C-E? If yes, please re-write it, it is unclear. For example:

1. a) You used pARF7::LUC, instead of DR5::LUC.
2. b) What is the growth zone?
3. c) ...to reflect the number of oscillation zone (OZ), or perhaps you meant oscillation, line535-536? Is ARF7 suitable for reflecting PBS spacing?
4. d) How many samples and biological repeats did you use for this experiment?

   We have re-written this section. In the case of ARF7, we only wanted to measure the expression levels rather than oscillations. The growth zone correspond to a root portion from the root meristem to about 3 cm shootwards.

Figures and Figure Legend:

Fig. 1.

1. Line 642: Light grown roots accumulate higher level of BiAux.

Fig. 2

1. Line 652: B) Primary root length and total root length (root length of the primary root plus eLRs)

of SKP2Bp::GUS seedlings grown...

- If you agree to use primary root length, please also change the y-axis label in B, left panel. 2. Data in C refers to the number SKP2BES in the post-transfer zone or whole root? In the whole root. Now is indicated in the legengd.

Fig. 3

1. Line 661-662: Note that the DR5::LUC expression is maintained in all PBS over time in BiAux

   treated roots, while its expression is not maintained in all PBS (asterisks) over time in mock-

   treated roots.

2. Line 666: Number of PBS specified in mock- or BiAux-treated roots per 24 h?
3. In C, y-axis, number of oscillations.
4. In D, y-axis, number of PBS/24h
5. In E, y-axis, percentage of PBS with maintained DR5 expression

Fig. 4

1. Line 674: A) Relative number of DR5ES in seedlings grown 4 days...
2. Data in A refers to the number of DR5ES in the post-transfer zone or whole root? In the whole root, since BiAux could reactivate dormant PBS or LRFC.
3. Line 676-678: Values represent the percentage relative to the mock average. Asterisks indicate

   significant differences by t-test in a genotype comparing mock and BiAux treatment. *, $p<0.05$; **, $p<0.01$; ***, $p<0.001$ by t-test. $n\geq15$ (1 biological replicate).

- The significance dose not match the one shown in A. Thank you for the call. This has been changed accordingly.

4. Data in D refers to the number of SKP2BES in the whole root? How old are those roots? It is indicated in the legend. They are 12 day –old and they were treated from germination with mock or BiAux..
5. A panel, y-axis label, relative number of DR5ES (%)
6. Line 683: ...1/2MS with mock or with mock or 1 or 5...
7. Line 689: Black arrows indicate SKP2BES in BiAux-treated *slr* roots.

Fig. 5

1. Color scale for B and C It has been included.

Fig. 6

1. Line 699:

B) Relative number of DR5ES...

2. B panel, y-axis label, relative number of DR5ES (%)
3. Data in D refers to the number DR5ES in the post-transfer zone or whole root? How old are those roots? In the whole root. It has been indicated.
4. Line 706: ...from the crystal structures of 2P1P (PDB id.) are...
5. In main text, you used "InsP6", here you used "IP6", it is better to use one consistently. We will use IP6

Fig. 7

1. Scale bar in A and scale of the scale bar in A legend. It has been included
2. B panel, y-axis label, relative number of DR5ES (%)
3. Line 718: Relative number of DR5ES...
4. Did you transfer the seedlings to the mock or BiAux plates, or just germinated the seeds on the mock or BiAux plates, how old were the seedlings did you use for the experiments? Please add the info. This has been indicated in the figure legend.

Supplemental Fig. S1

1. The figure legend you put in the main text is different from the one in the pdf file (the one with the figures), and apparently the one in the **main text** is the good one. The bellowing suggestions were made based on the one from **main text**. Yes, we duplicated Fig S2 legend by mistake. I apology for the inconvenience.
2. Line 750: I didn't see the "orange ellipse" in Fig. S1B, instead I saw a cyan and a yellow one.
3. Line 755: Fig. S1D: eLR or LR (when the primordium breaks through the main root epidermis) is usually no longer considered as a primordium anymore. I would suggest just to use "number of SKP2BES". If you agree, please do NOT forget to change the label in the figure, y-axis. I appreciate the suggestion. It has been modified.

Supplemental Fig. S2

1. The figure legend you put in the main text is different from the one in the pdf file (the one with the figures), and apparently the one in the **pdf file** is the good one. The bellowing suggestions were made based on the one from **pdf file**.
2. BiAux, but not the intermediates used for its synthesis, increases LR formation.
3. C) Number of SKP2Bp::GUS expression sites (SKP2BES) or emerged lateral root (eLR)...
4. E) Number of DR5::LUC expression sites (DR5ES) in Arabidopsis...
5. How old are those roots in E? It is now indicated in the legend /(4 days in MS1/2 and then 3 days in mock or BiAux containing medium)
6. Asterisks indicate...
7. In E, "pre-transfer" instead of "pret-transfer".
8. Please indicate the scale of the scale bars presented B and D in the legend.

Supplemental Fig. S3

1. The bellowing suggestions were made based on the one from **main text**.
2. Line779:...5µMofBiAuxor5,10,25,50100,500or1000nMofIAAforextra?days.C)

   Root growth and number of DR5ES in DR5::LUC roots grown...

3. If you agree to use DR5ES, please change the labels for y-axis in C, “number of DR5ES

   pre/post-transfer”.

4. Scale bars in A and B, and please indicate the scale of the bars in the legend.
5. Please indicate in the legend, the significance in C was done by....

Supplemental Fig. S4

1. The figure legend you put in the main text is different from the one in the pdf file (the one with the figures), and apparently the one in the **main text** is the good one. The bellowing suggestions were made based on the one from **main text**.
2. Line 787-788: From left to right, average meristematic cell length (unit?), average number of meristematic cortical cell number and meristem size (µm) were quantified...
3. In A, the most left panel, the label of y-axis: “average cell length” instead of “cell lenght average”, and also the unit is missing here.
4. Line 790: Asterisks indicate...

Supplemental Fig. S5

1. B panel, the color scale is missing for the left one, the green and red heatmap.

Supplemental Fig. S7

1. The bellowing suggestions were made based on the one from **main text**.
2. Line 803: Number of docking analyses and free energy (kal/mol) estimation between BiAux

   and Arabidopsis TIR1...

3. Line 809: Scale of the scale bar (coin)
4. Line 812: ...and in tomato... Template modeling (TM)-score and root-mean-square deviation

   (RMSD) between them are 0.993 and 0.407 A (569/581 residues), demonstrating that...

Supplemental Fig. S8

1. The bellowing suggestions were made based on the one from **main text**.
2. Color scale for B
3. For D and E, how many samples and biological repeats did you include? Four roots were quantified.
4. The description from Line 534-536 in materials and methods, is it for this experiment? This has been changed in M&M

Supplemental Fig. S9

1. The bellowing suggestions were made based on the one from **main text**.
2. Line 833: BiAux regulated genes that are preferentially expressed in phase with the DR5::LUC expression in the OZ
3. Line 835: ...in BiAux treated roots...phase or antiphase in the OZ.
4. Line 836-837: **B**) Venn diagrams of the differentially expressed genes (up- or down-regulated) in wild type roots treated by BiAux, and...in *arf7-1* or *potent* roots.
5. Line 840-841: ...containing mock or BiAux for 2 days. Yellow lines indicate the root area above the root meristematic zone with increased DR5::LUC signals.

Supplemental Fig. S10

1. The bellowing suggestions were made based on the one from **main text**.
2. Line 845: ...number of DR5ES in DR5::LUC...
3. Data in B refers to the number of DR5ES in the post-transfer zone or whole root? It corresponds to the whole root.
4. B panel, y-axis label, relative number of DR5ES (%)

Supplemental Fig. S12

1. You used “Biaux” at some places in the figure.

13-Jan-2024
Prof. Juan Carlos del Pozo
Centro de Biotecnologia y Genomica de Plantas (CBGP) UPM-INIA/CSIC
biotechnology
Pozuelo de Alarcon, Madrid 28040
Spain

MSID: PP2023-RA-00900R2
MS TITLE: BiAux modulates auxin co-receptor activity to stimulate lateral root formation

Dear Dr. Juan del Pozo:

I am pleased to inform you that your revised manuscript "BiAux modulates auxin co-receptor activity to stimulate lateral root formation" has been accepted for publication in Plant Physiology. Your manuscript will be checked for consistency with journal formatting and image standards by one of our Science Editors, who will contact you shortly. They may have a number of queries and suggestions for you. Please keep in mind that they work to increase the online visibility of your article and its attraction for readers from the widest of backgrounds. You will have the opportunity to review any revisions, and I hope you will work constructively with them to get the most out of your research. You will also have the opportunity to review all changes when you receive the author proofs for your article.

Once validated for publication, your article will be sent to a Science Editor for a final review of your manuscript and source files. After your final files are uploaded, your article will appear in the next available issue and the online Preview version of your article will be posted after your signed Copyright and Checklist forms and final source files are received.

Please be sure to include the following in your manuscript file:
Harvard style citations
List of all author contributions and funding information at the beginning of manuscript after the titles and author list.
List all supplemental materials associated with the article, with titles, at the end of the ms, before the references and acknowledgments section.
Complete Figure Legends for all figures appearing in the article listed before the Literature Cited.

Thank you and congratulations on your Plant Physiology paper!

Sincerely,

Plant Physiology
Board of Editors

=====
FIRST AUTHOR PROFILES
=====

We recognize the hard work that goes into being First Author of a Plant Physiology paper by publishing a "First Author Feature" for each First Author alongside your paper.

If the first author(s) would like to submit information for a feature on Facebook and Twitter, fill out the form by clicking on the link below:

Plant Physiology First Author Feature Form: https://forms.office.com/r/ZBCGZfhm8F
If you cannot access the form through the link above, please contact Rachel at rbelsky@aspb.org.

To upload an image of yourself to accompany your profile, please put your photo in the correct journal folder at the link below. You must label your image: Author First Name Last Name Article Title.

First Author Profile Image Dropbox: https://www.dropbox.com/scl/fo/xz029p7qjr2jml2rp2kmq/h?dl=0&rlkey=6fkxcaqmxx35ap152b2osy1fz

=====
IMPORTANT REMINDER: PEER REVIEW REPORTS
=====

If you opted to publish a peer review report along with your article during the original submission process, it will be prepared by the editorial staff and publicly posted with your manuscript, inside the zip file that contains any other supplemental material. As a reminder, the peer review report is a public record of all comments from editors and reviewers, as well as your prior responses, as you received them in the decision letters for each draft of your manuscript. If you agreed to publish this report and have changed your mind, or are not sure if you selected this option, please contact the editorial office as soon as possible before signing the license agreement from our publisher.

-- Reviewer comments:
